# Supplementary material for: Genotyping-by-sequencing and SNP-arrays are complementary for detecting quantitative trait loci by tagging different haplotypes in association studies
Source: BMC Plant Biol. 2019 Jul 16;19:318. doi: 10.1186/s12870-019-1926-4 (PMC6636005; doi:10.1186/s12870-019-1926-4)
Supplement: Supplementary file 1 — Figure S1. Different approaches used to impute missing data of the GBS. We considered the direct reads from GBS (GBS1) and four approaches for imputation (GBS2 to GBS5). GBS2 approach consisted in one imputation step from the direct read by Cornell University, using TASSEL software, but missing data was still present. GBS3 approach consisted in a genotype imputation of the whole missing data of the direct read by Beagle v3. In GBS4, genotype imputation by Beagle was performed on Cornell imputed data after replacing the heterozygous genotypes into missing data. GBS5, consisted in homozygous genotypes of GBS2 completed by values imputed in GBS3. (DOCX 14 kb) [file 12870_2019_1926_MOESM1_ESM.docx]

**Figure S1: Different approaches used to impute missing data of the GBS.**

We considered the direct reads from GBS (GBS_1_) and four approaches for imputation (GBS_2_ to GBS_5_). GBS_2_ approach consisted in one imputation step from the direct read by Cornell University, using *TASSEL* software, but missing data was still present. GBS_3_ approach consisted in a genotype imputation of the whole missing data of the direct read by *Beagle v3*. In GBS_4_, genotype imputation by Beagle was performed on Cornell imputed data after replacing the heterozygous genotypes into missing data. GBS_5_, consisted in homozygous genotypes of GBS_2_ completed by values imputed in GBS_3_.
