# Supplementary material for: Genotyping-by-sequencing and SNP-arrays are complementary for detecting quantitative trait loci by tagging different haplotypes in association studies
Source: BMC Plant Biol. 2019 Jul 16;19:318. doi: 10.1186/s12870-019-1926-4 (PMC6636005; doi:10.1186/s12870-019-1926-4)
Supplement: Supplementary file 2 — Figure S2. Comparison of genotyping data between 50K and 600K arrays, and GBS. (a) Distribution of minor allele frequency per SNP before filtering (monomorphic SNPs removed). (b) Distribution of SNP missing data proportion for the 50K array, 600K array, GBS direct reads (GBS1) and GBS after imputation by Cornell Institute (GBS2, note that the scale of the x-axes is different). (c) Relatedness distribution (Identity-By-State, IBS) after QC filtering with MAF≥1% (IBS using GBS1 was not estimated because of the low calling rate). (DOCX 71 kb) [file 12870_2019_1926_MOESM2_ESM.docx]

**Figure S2: Comparison of genotyping data between 50K and 600K arrays, and GBS.**

(a**)** Distribution of minor allele frequency per SNP before filtering (monomorphic SNPs removed). (b**)** Distribution of SNP missing data proportion for the 50K array, 600K array, GBS direct reads (GBS_1_) and GBS after imputation by Cornell Institute (GBS_2_, note that the scale of the x-axes is different). (**c**) Relatedness distribution (Identity-By-State, IBS) after QC filtering with MAF≥1% (IBS using GBS_1_ was not estimated because of the low calling rate).
