# Supplementary material for: Genotyping-by-sequencing and SNP-arrays are complementary for detecting quantitative trait loci by tagging different haplotypes in association studies
Source: BMC Plant Biol. 2019 Jul 16;19:318. doi: 10.1186/s12870-019-1926-4 (PMC6636005; doi:10.1186/s12870-019-1926-4)
Supplement: Supplementary file 7 — Figure S7. QTL limits obtained by the LD_adj approach projected on heatmaps representing the level of LD between associated SNPs for each trait (DTA: male flowering time, plantHT: plant height and GY: grain yield) and each chromosome. Upper and lower triangles on the heatmaps represent the r2 and r2K values between associated SNPs, respectively. Linkage disequilibrium between loci was colored according to values from weak LD (yellow) to high LD (red). The significant markers were ordered according to their physical positions on the chromosome and were represented by ticks on the four sides of the heatmaps. Limits of QTLs were displayed by gray dotted lines. QTL numbers were indicated in gray on the top and the right of each heatmap. (PDF 7373 kb) [file 12870_2019_1926_MOESM7_ESM.pdf]

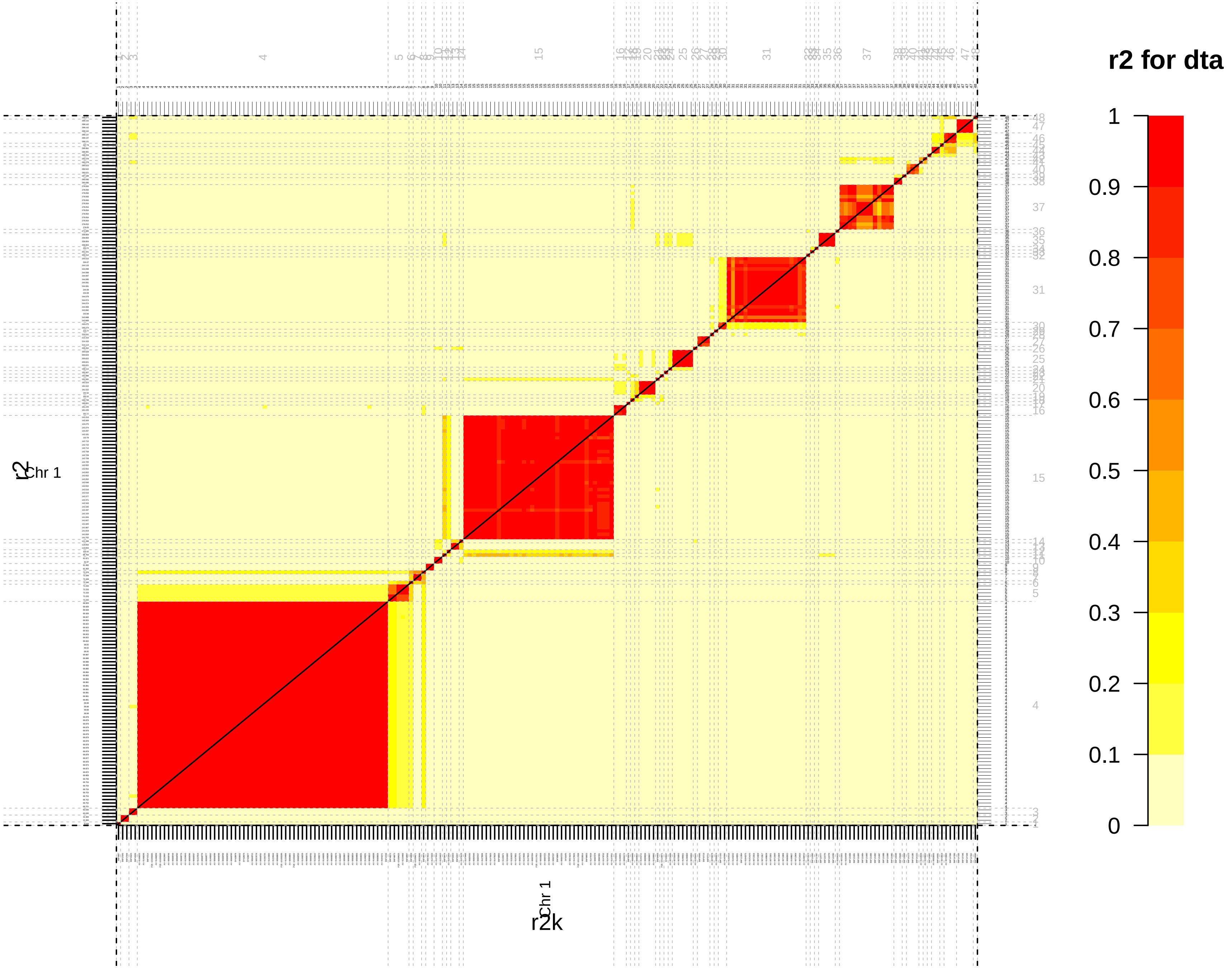
r2 for dta
1
0.9
0.8
0.7
0.6
0.5
0.4
0.3
0.2
0.1
0
Chr 1
r2
Chr 1
r2k

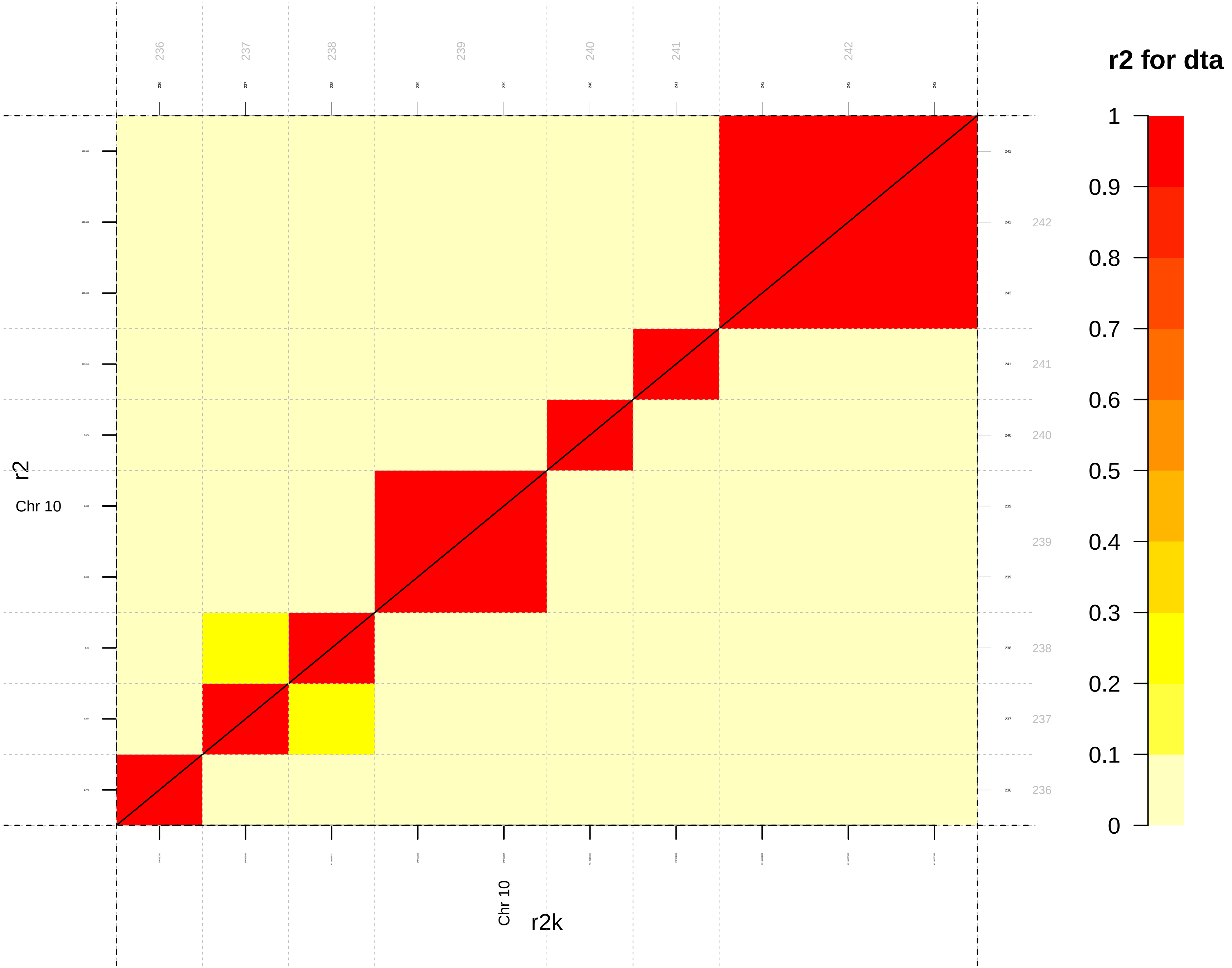

r2 for dta
1
0.9
0.8
0.7
0.6
0.5
0.4
0.3
0.2
0.1
0
236
237
238
239
240
241
242
r2
Chr 10
r2k

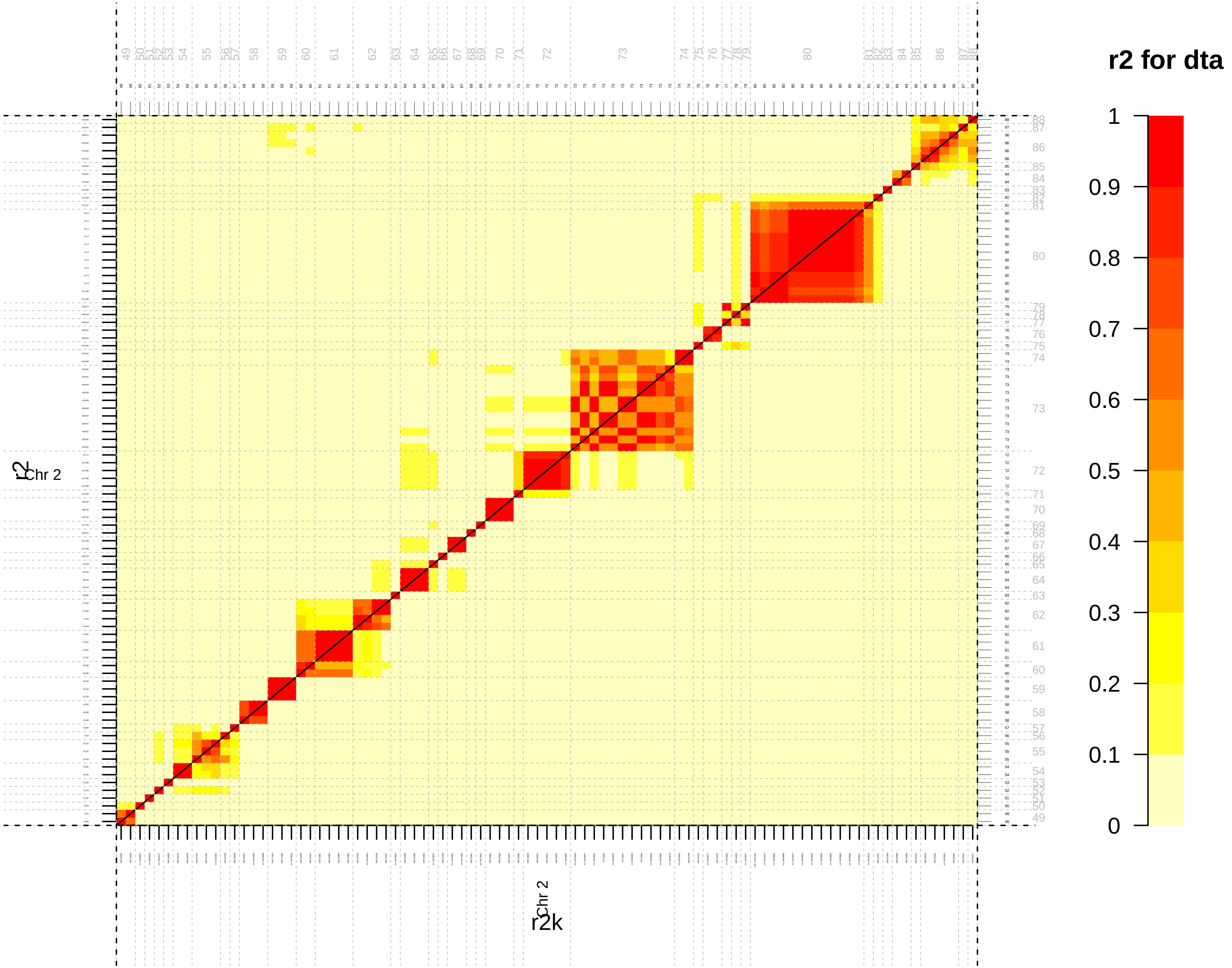
r2 for dta
1
0.9
0.8
0.7
0.6
0.5
0.4
0.3
0.2
0.1
0
Chr 2
r2
Chr 2
r2k

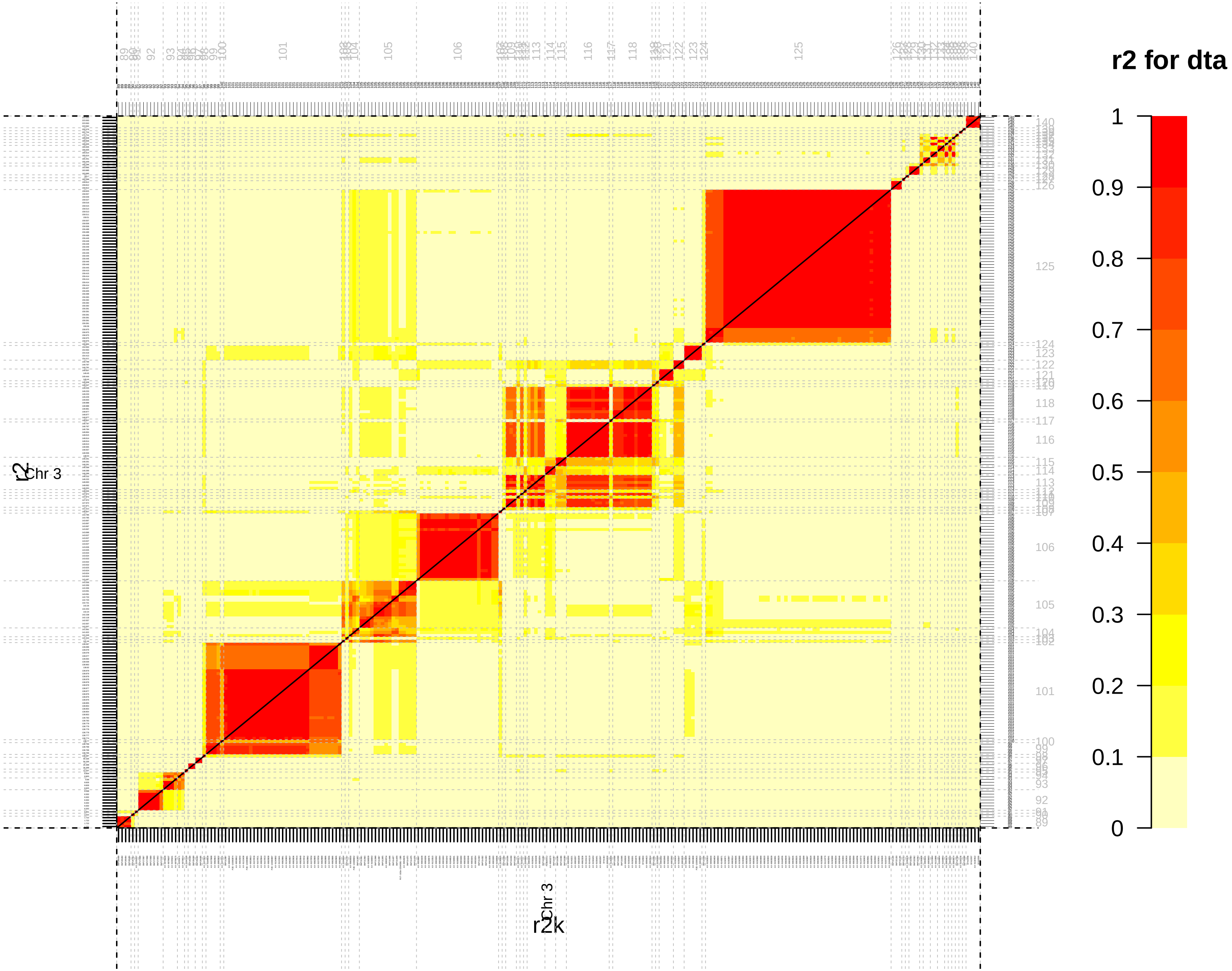
r2 for dta
1
0.9
0.8
0.7
0.6
0.5
0.4
0.3
0.2
0.1
0
Chr 3
r2
Chr 3
r2k

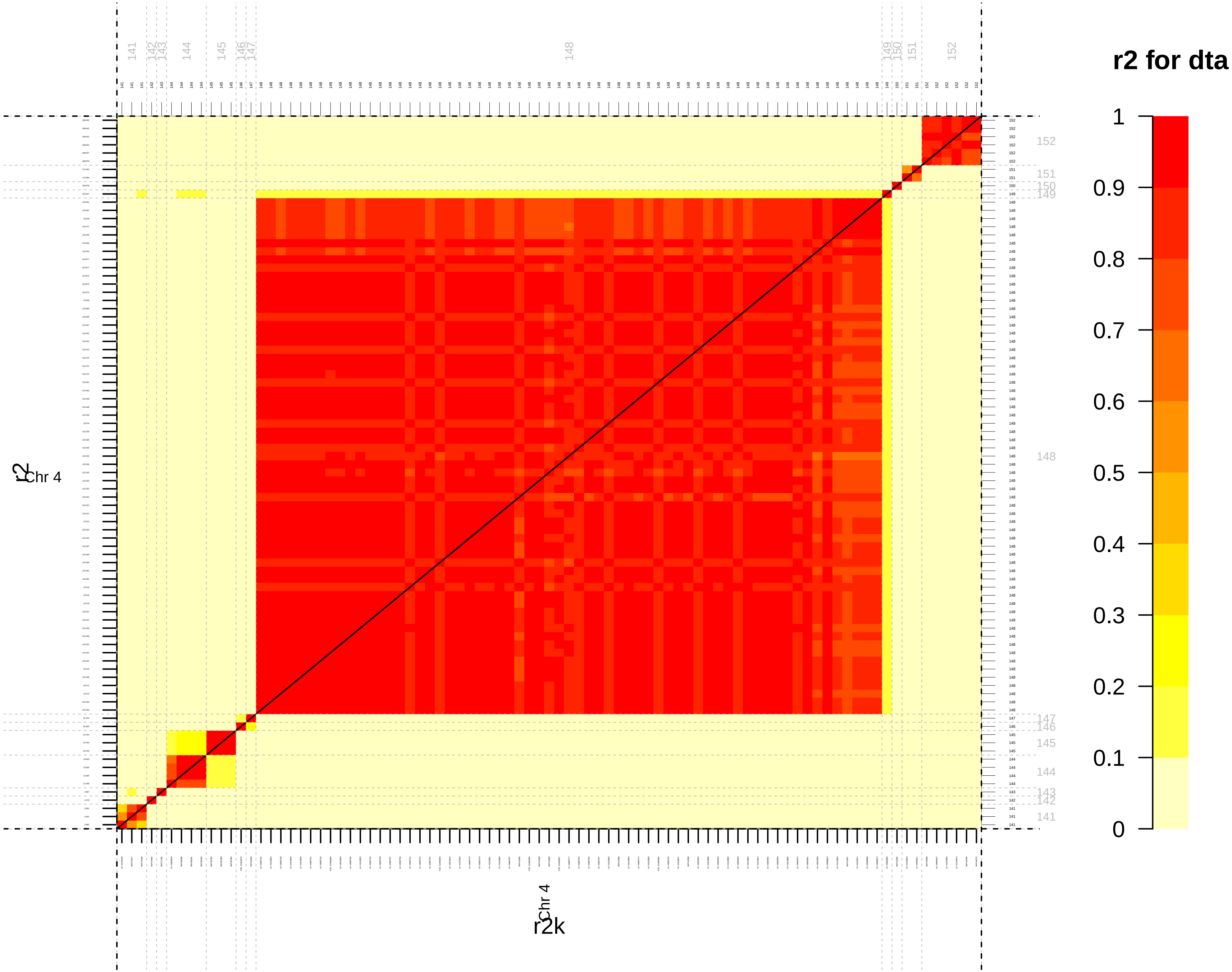
r2 for dta
1
0.9
0.8
0.7
0.6
0.5
0.4
0.3
0.2
0.1
0
141
142
143
144
145
146
147
148
149
150
151
152
Chr 4
r2
Chr 4
r2k

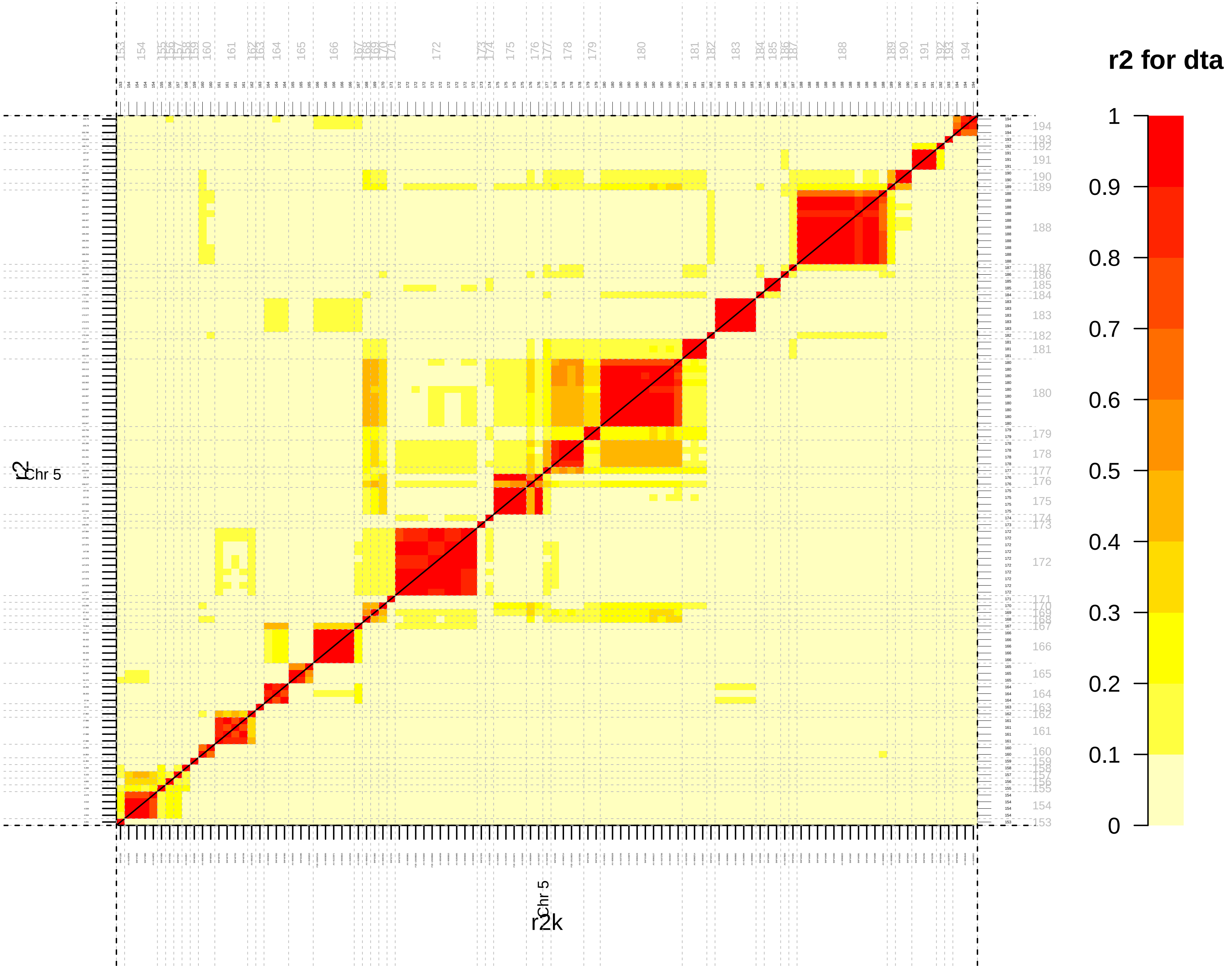
r2 for dta
1
0.9
0.8
0.7
0.6
0.5
0.4
0.3
0.2
0.1
0
r2
Chr 5
Chr 5
r2k

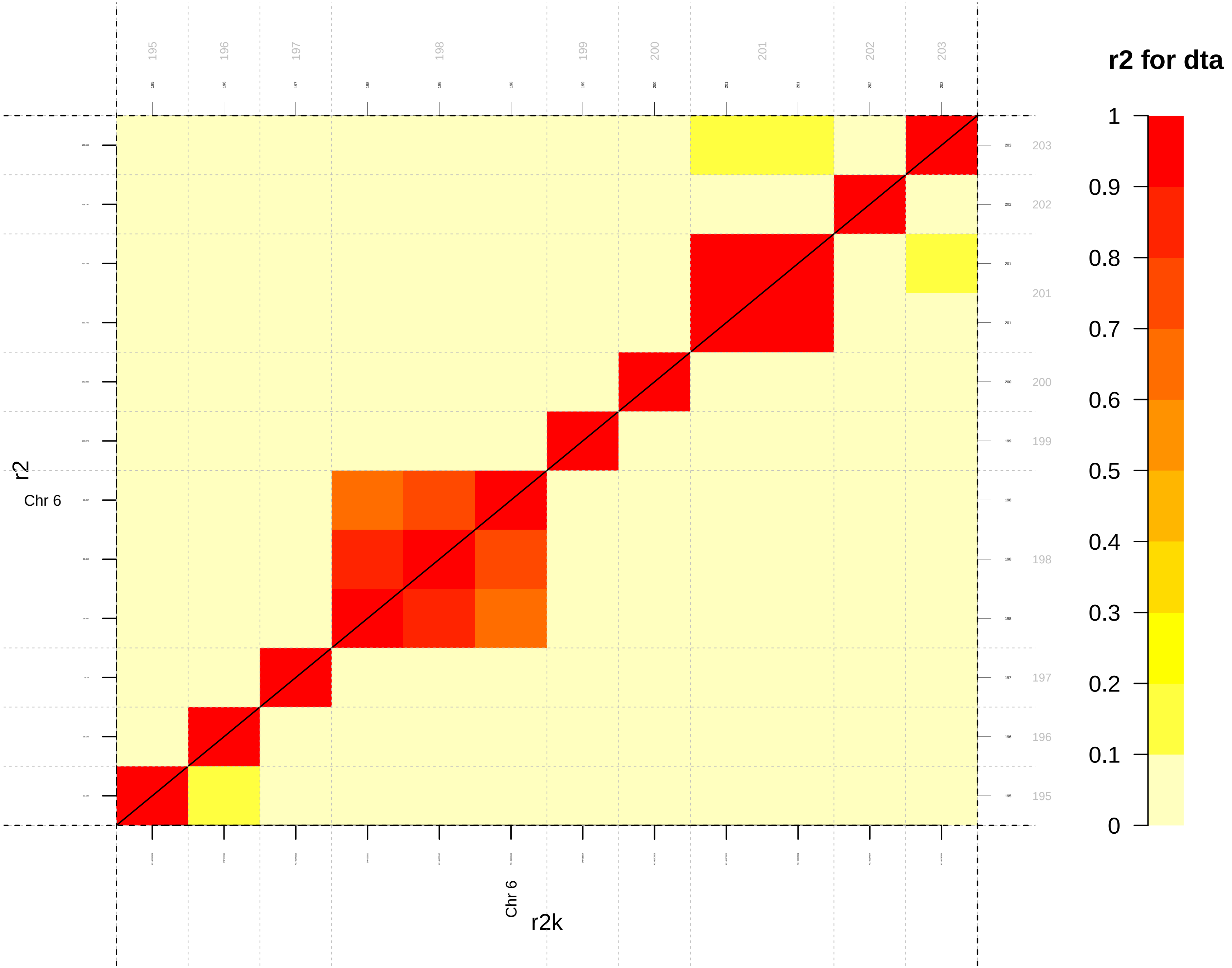
r2 for dta
1
0.9
0.8
0.7
0.6
0.5
0.4
0.3
0.2
0.1
0
195
196
197
198
199
200
201
202
203
r2
Chr 6
r2k
Chr 6

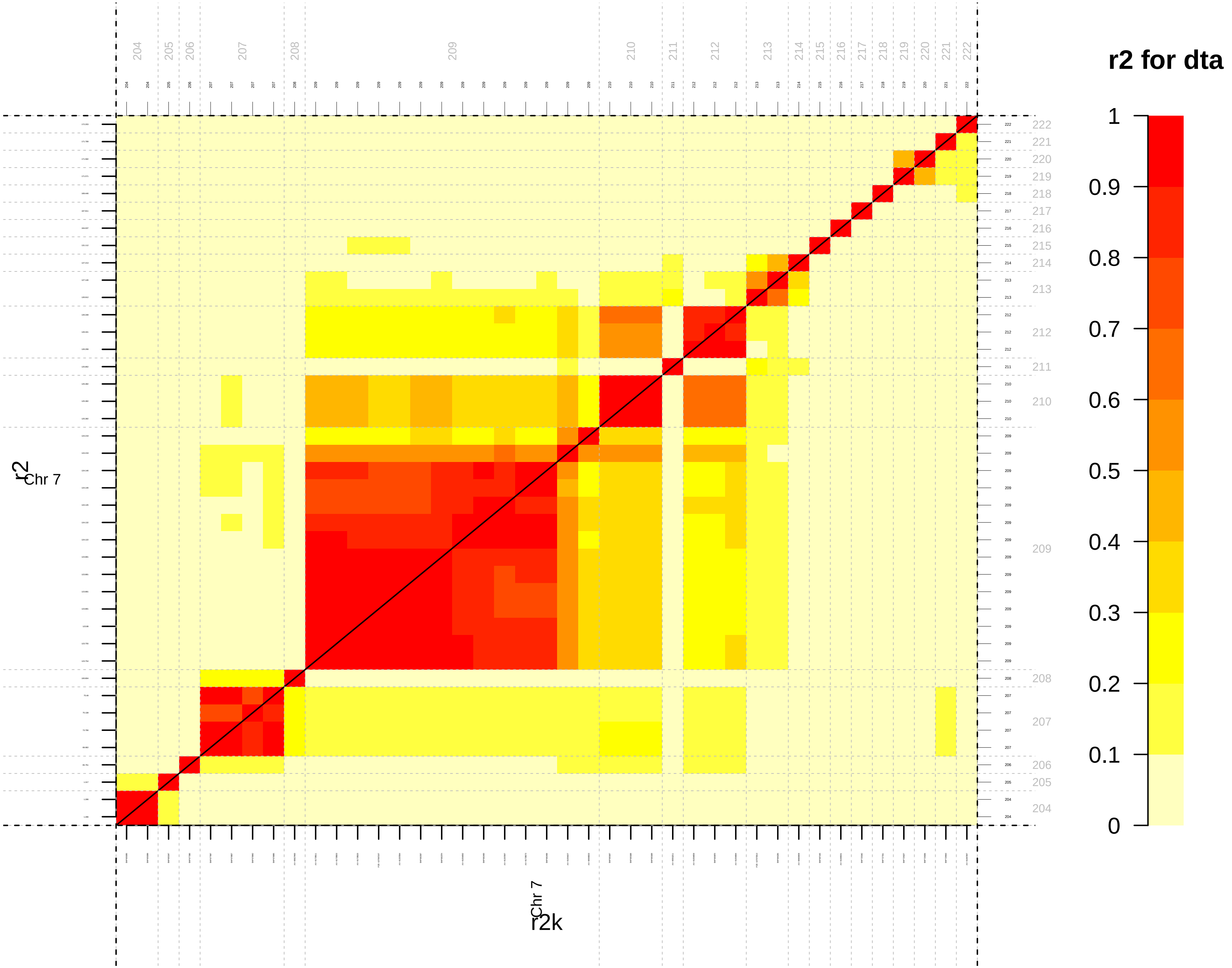
r2 for dta
1
0.9
0.8
0.7
0.6
0.5
0.4
0.3
0.2
0.1
0
204
205
206
207
208
209
210
211
212
213
214
215
216
217
218
219
220
221
222
r2
Chr 7
r2k

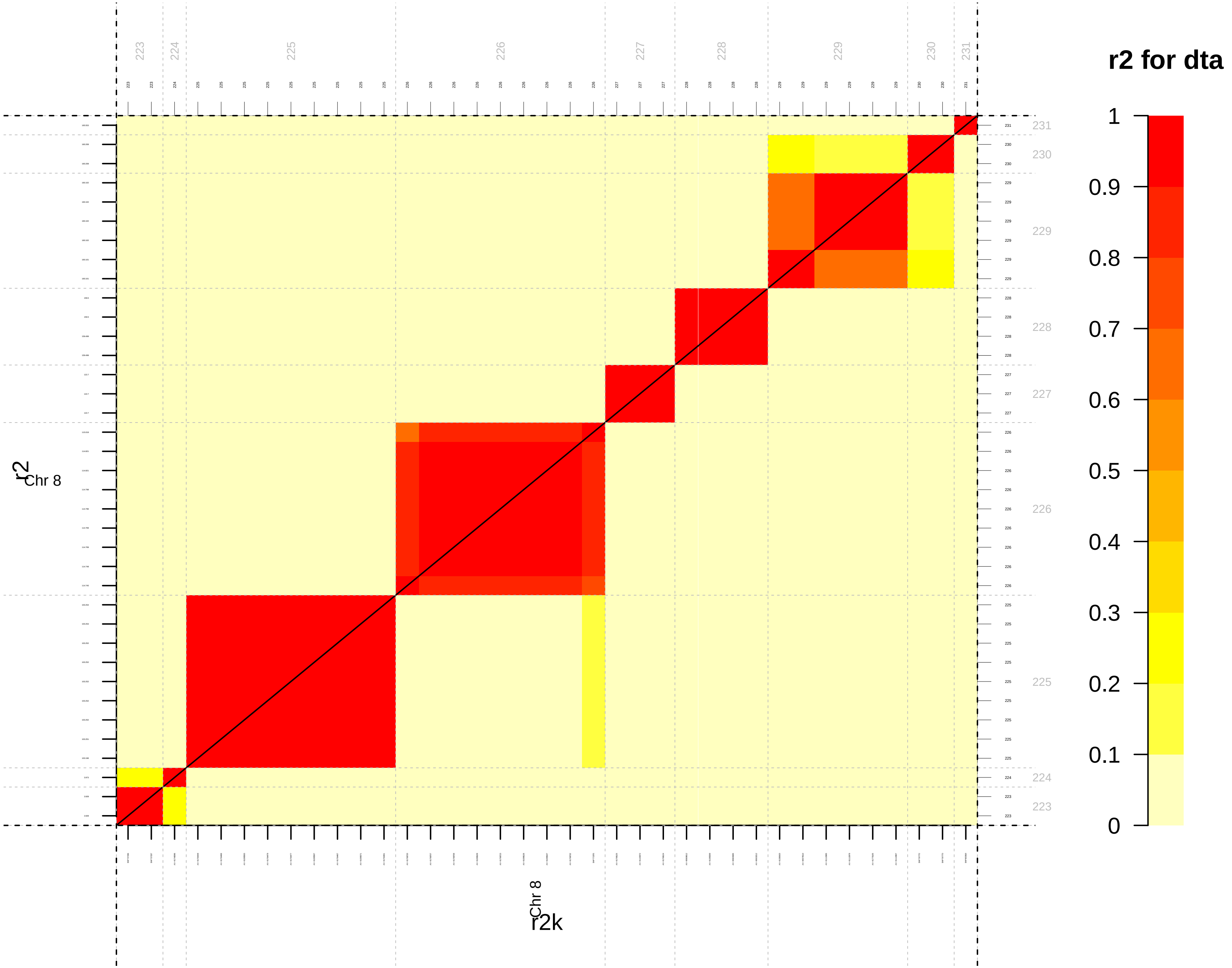
r2 for dta
1
0.9
0.8
0.7
0.6
0.5
0.4
0.3
0.2
0.1
0
223
224
225
226
227
228
229
230
231
r2
Chr 8
r2k
Chr 8

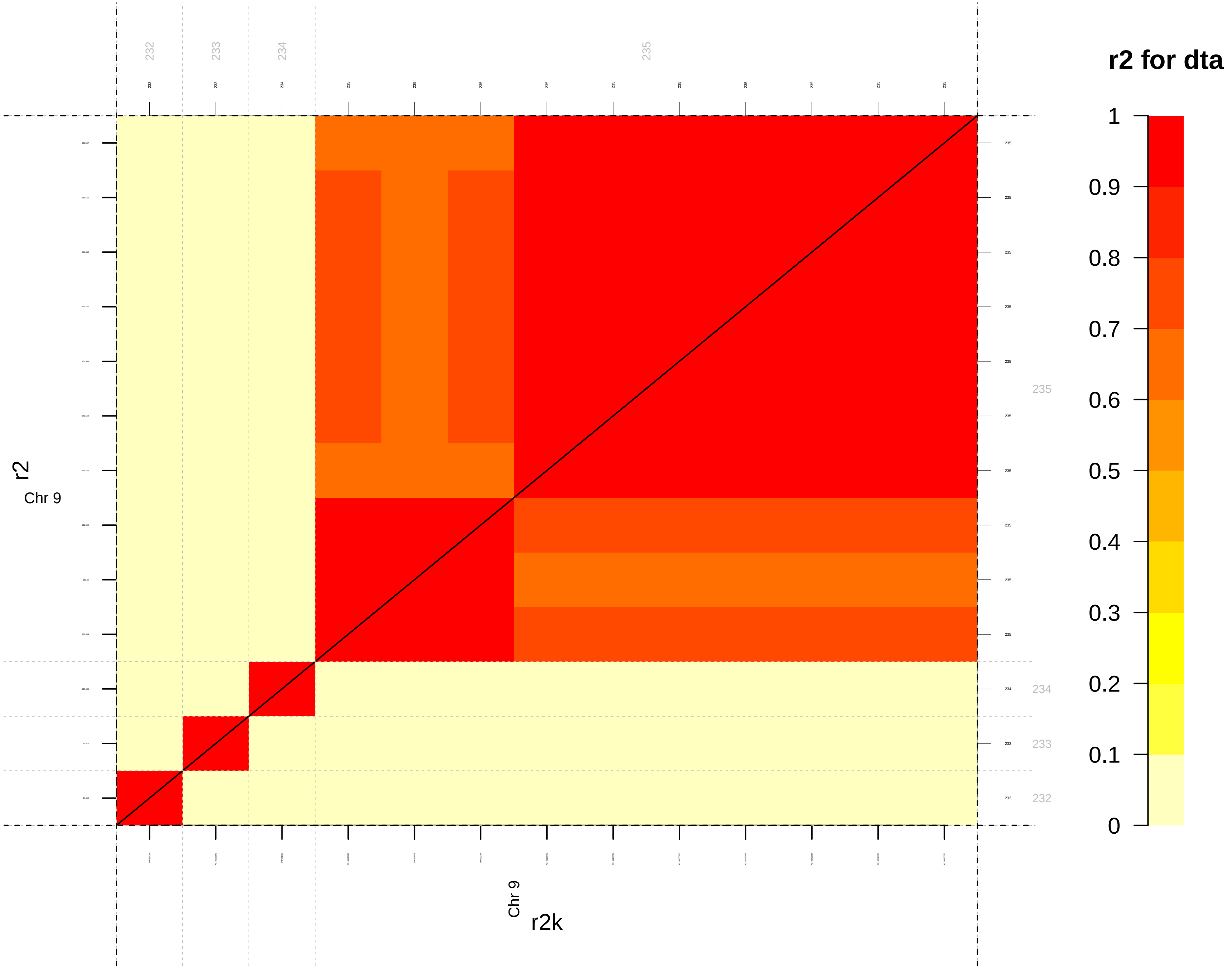

r2 for dta
1
0.9
0.8
0.7
0.6
0.5
0.4
0.3
0.2
0.1
0
232
233
234
235
r2
Chr 9
Chr 9
r2k

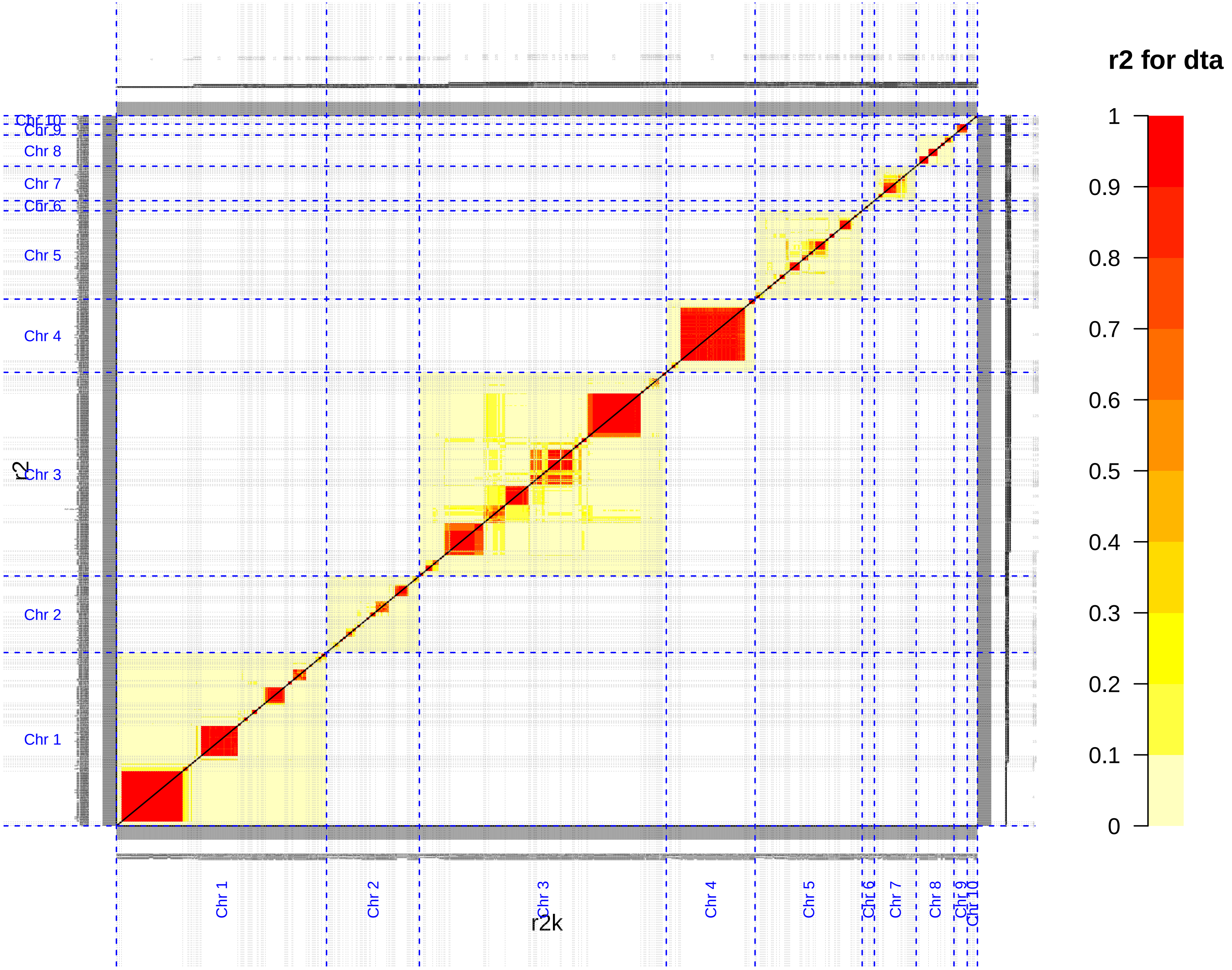

r2 for dta
1
0.9
0.8
0.7
0.6
0.5
0.4
0.3
0.2
0.1
0
r2
r2k
Chr 1
Chr 2
Chr 3
Chr 4
Chr 5
Chr 6
Chr 7
Chr 8
Chr 9
Chr 10

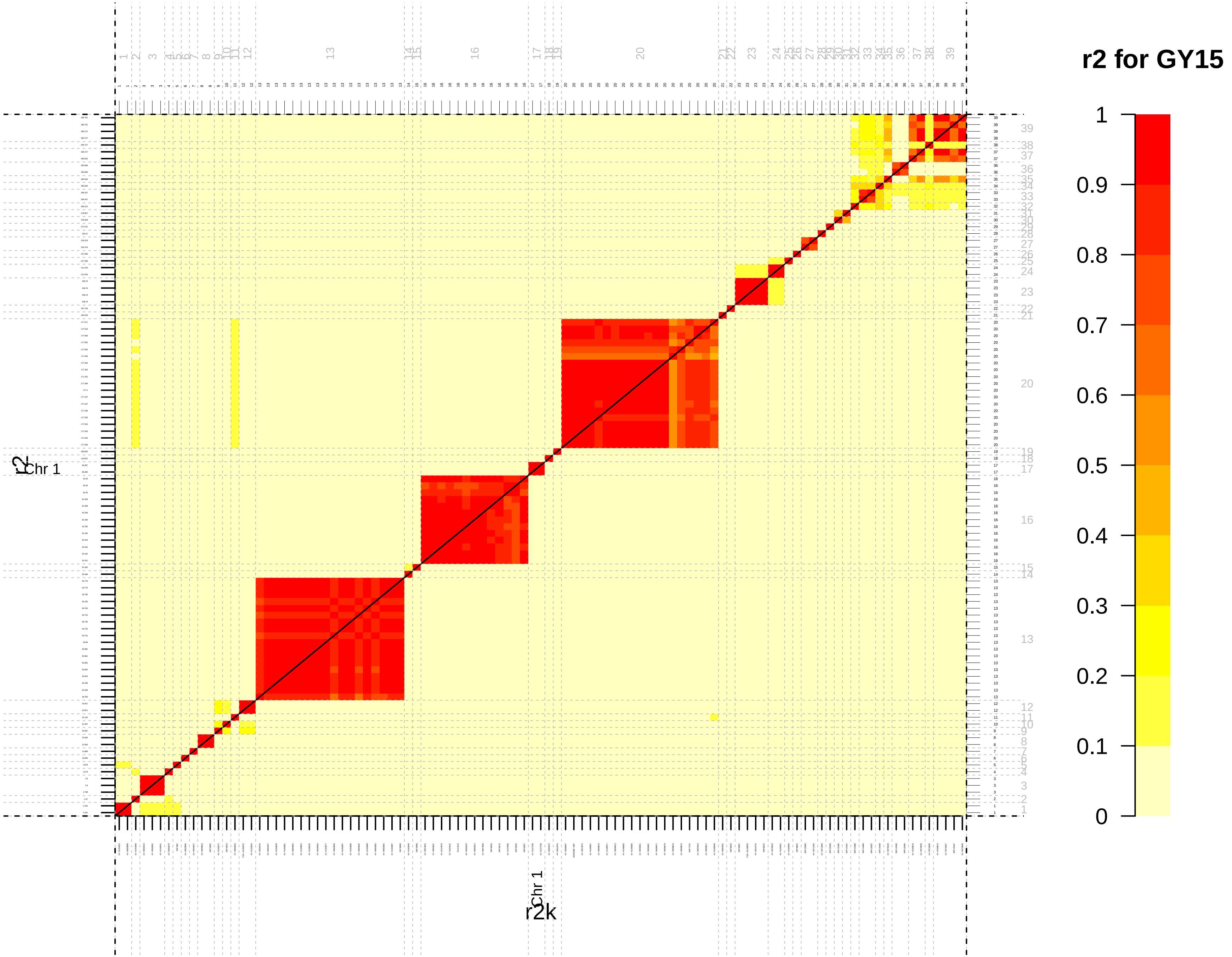
r2 for GY15
1
0.9
0.8
0.7
0.6
0.5
0.4
0.3
0.2
0.1
0
Chr 1
r2
r2k

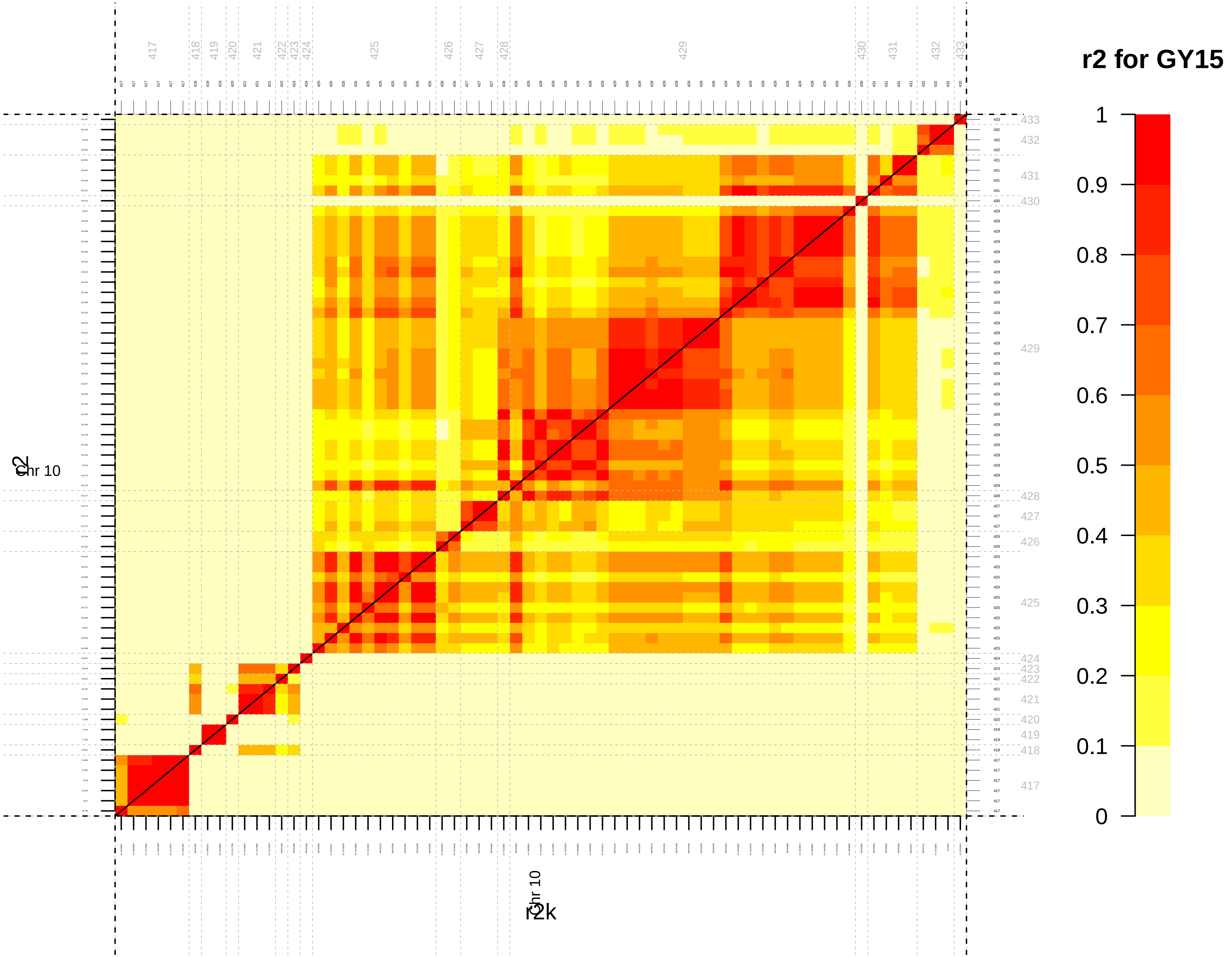
r2 for GY15
1
0.9
0.8
0.7
0.6
0.5
0.4
0.3
0.2
0.1
0
417
418
419
420
421
422
423
424
425
426
427
428
429
430
431
432
433
Chr 10
r2
Chr 10
r2k

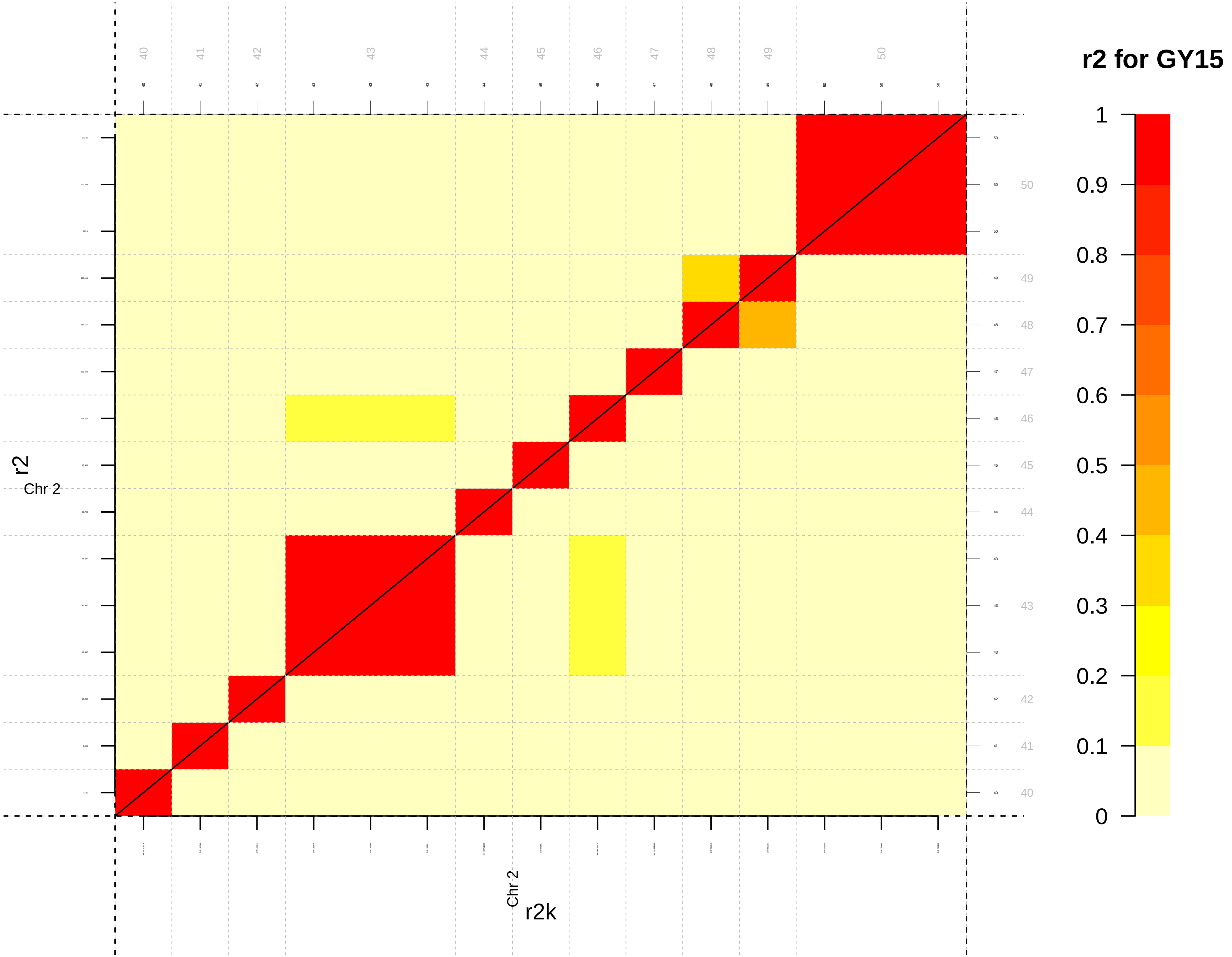

r2 for GY15
r2
Chr 2
r2k
Chr 2
40
41
42
43
44
45
46
47
48
49
50
1
0.9
0.8
0.7
0.6
0.5
0.4
0.3
0.2
0.1
0

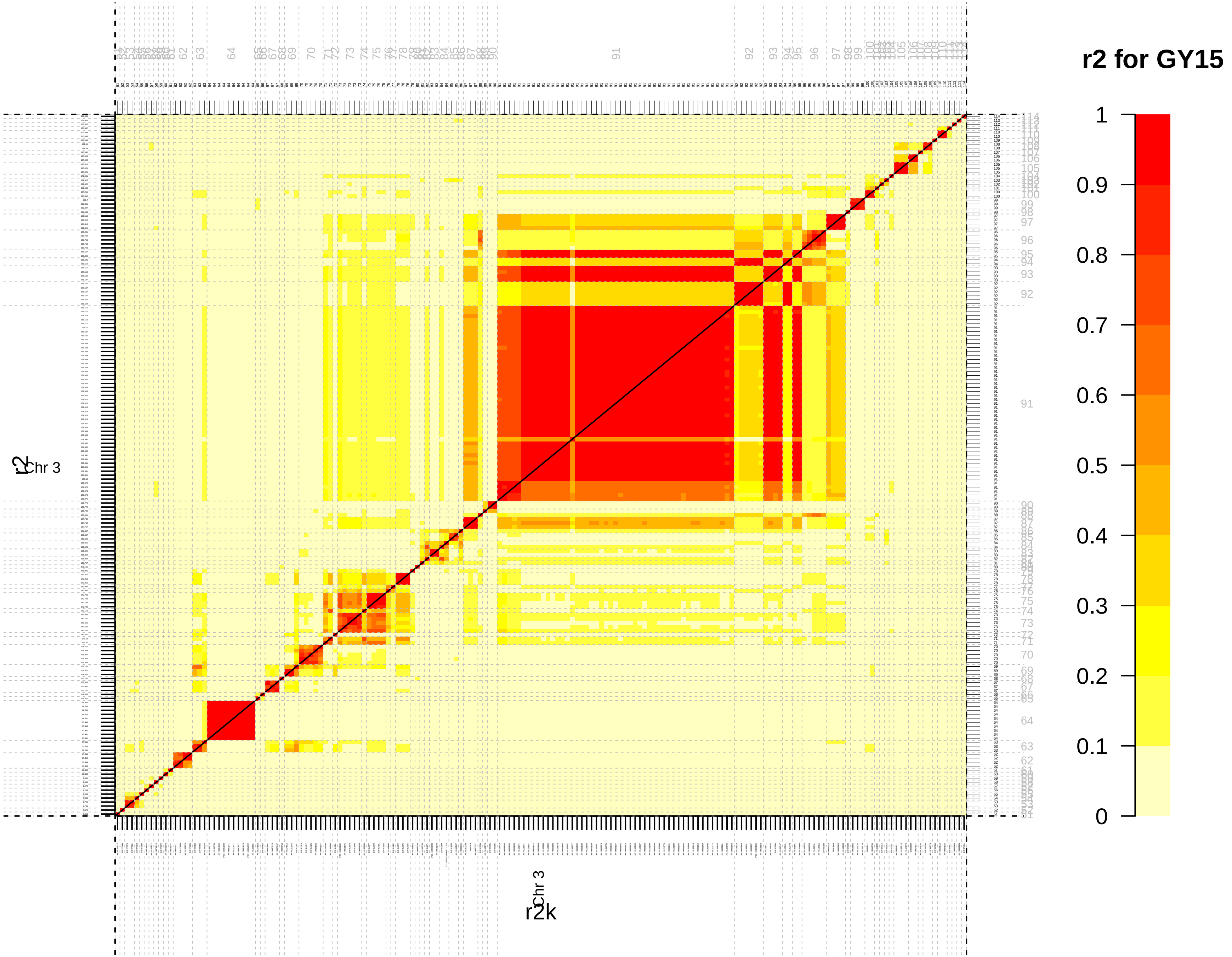
r2 for GY15
1
0.9
0.8
0.7
0.6
0.5
0.4
0.3
0.2
0.1
0
Chr 3
r2
Chr 3
r2k

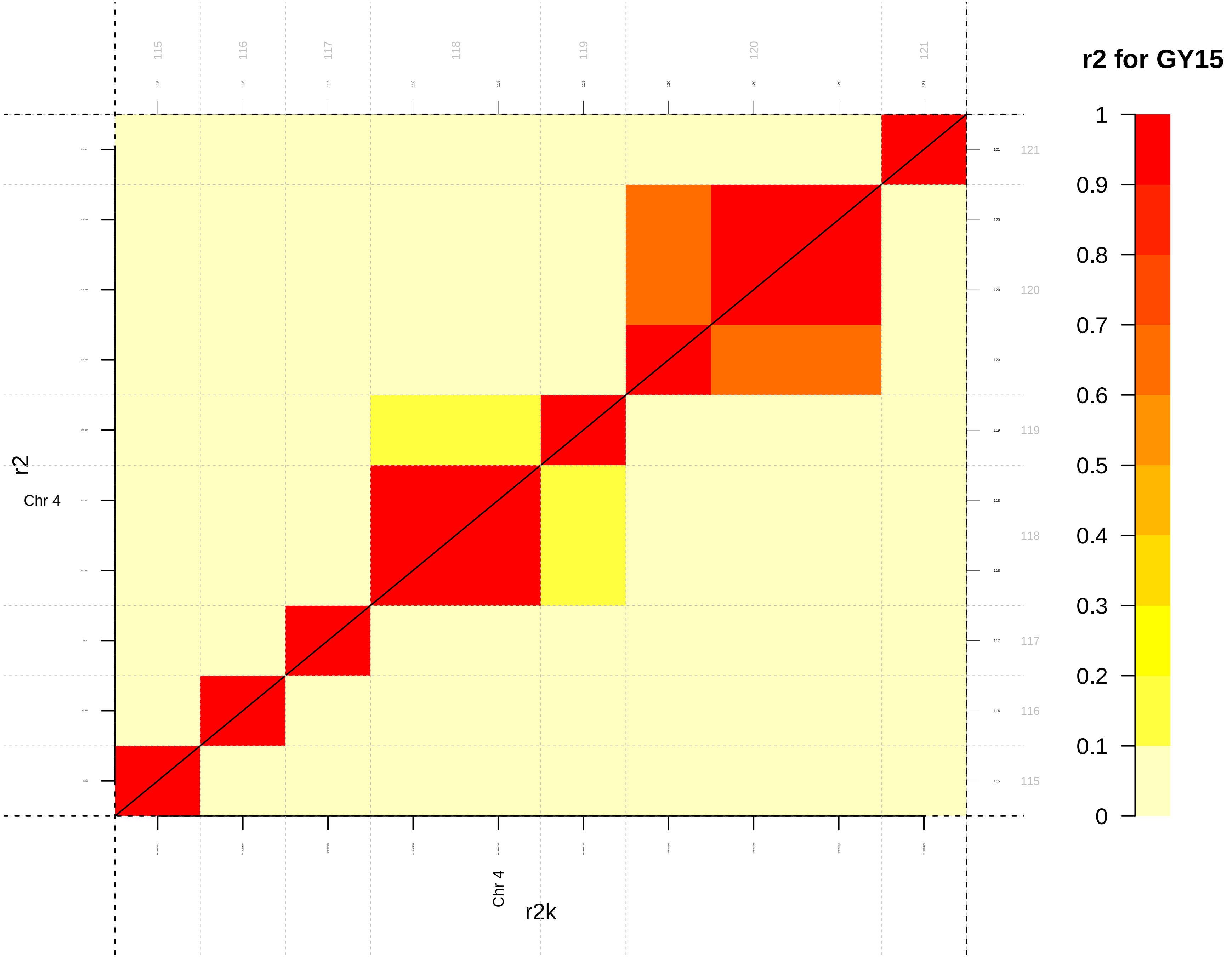

r2 for GY15
1
0.9
0.8
0.7
0.6
0.5
0.4
0.3
0.2
0.1
0
115
116
117
118
119
120
121
r2
Chr 4
r2k

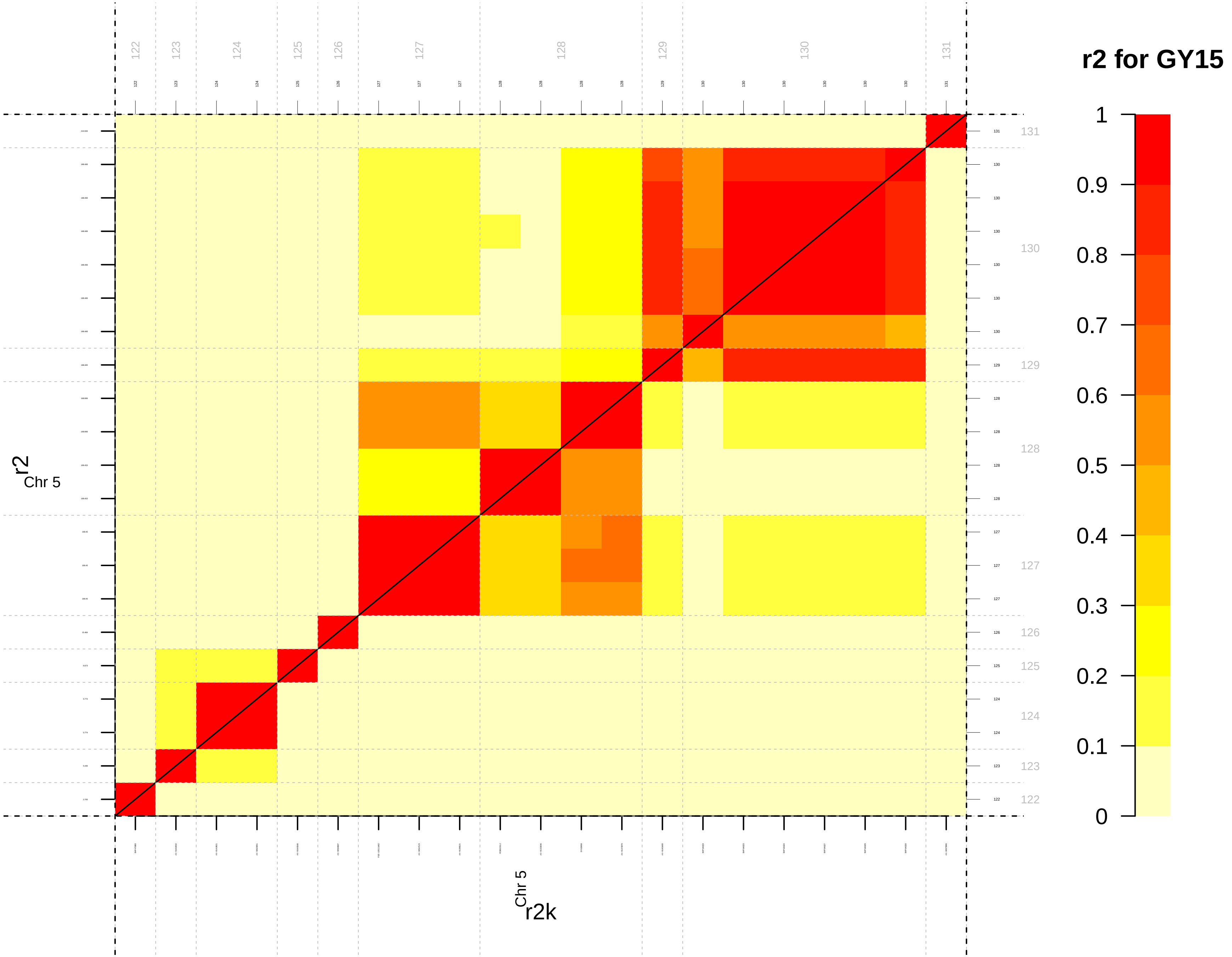

r2 for GY15
r2
Chr 5
r2k
Chr 5
122
123
124
125
126
127
128
129
130
131
1
0.9
0.8
0.7
0.6
0.5
0.4
0.3
0.2
0.1
0

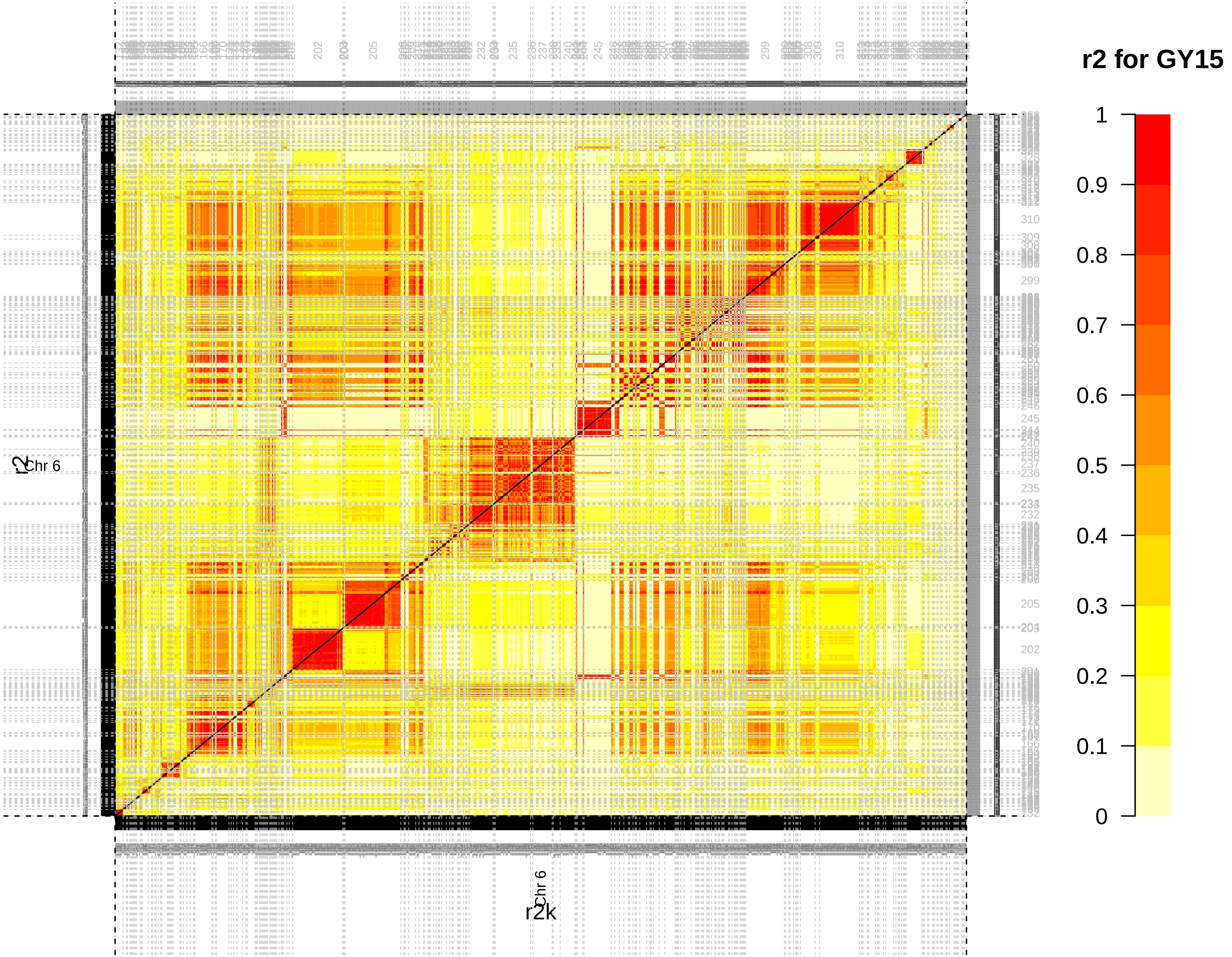
r2 for GY15
1
0.9
0.8
0.7
0.6
0.5
0.4
0.3
0.2
0.1
0
Chr 6
r2
Chr 6
r2k

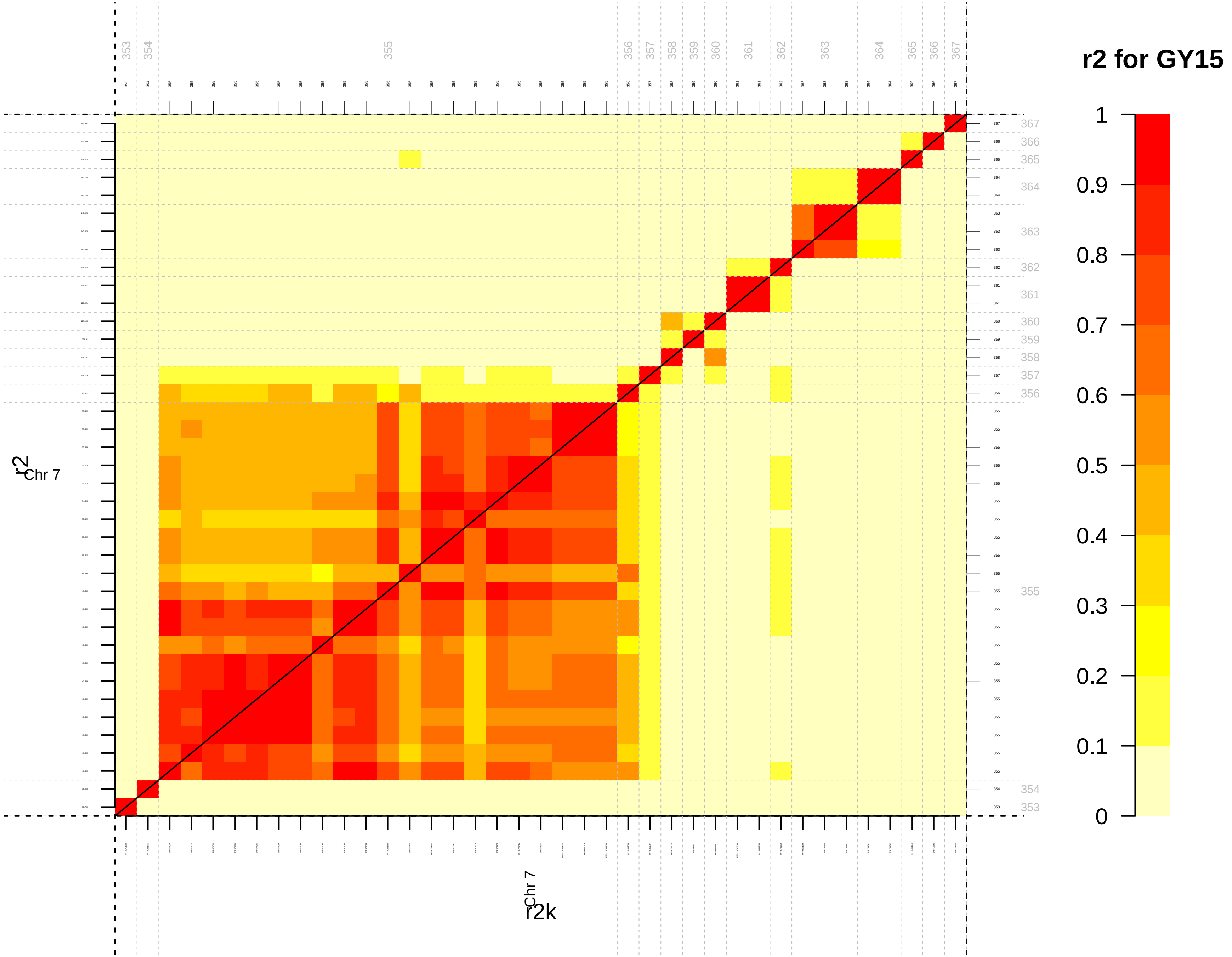

r2 for GY15
1
0.9
0.8
0.7
0.6
0.5
0.4
0.3
0.2
0.1
0
r2
Chr 7
r2k
Chr 7
353
354
355
356
357
358
359
360
361
362
363
364
365
366
367

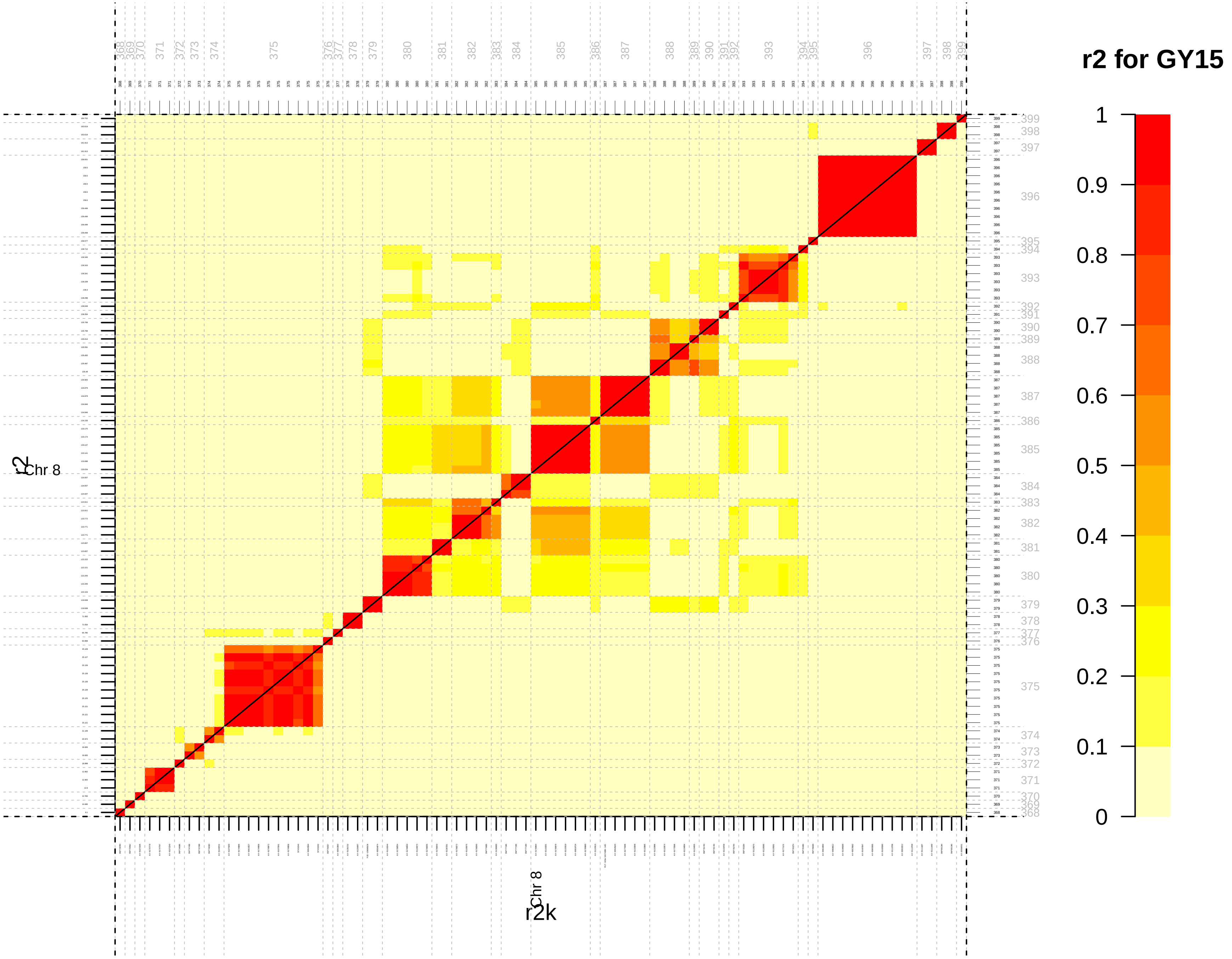
r2 for GY15
1
0.9
0.8
0.7
0.6
0.5
0.4
0.3
0.2
0.1
0
368
369
370
371
372
373
374
375
376
377
378
379
380
381
382
383
384
385
386
387
388
389
390
391
392
393
394
395
396
397
398
399
r2
Chr 8
r2k
Chr 8

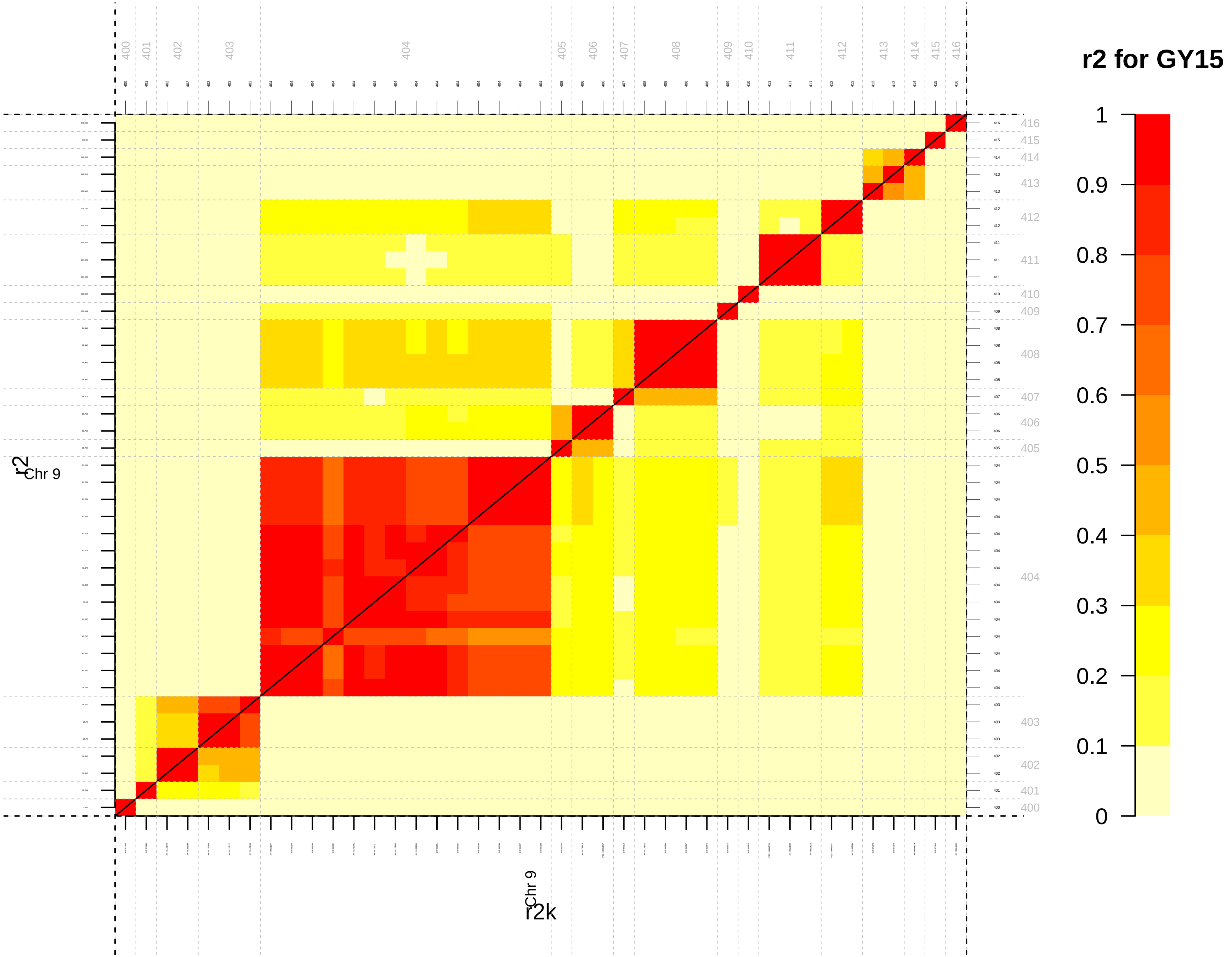

r2 for GY15
1
0.9
0.8
0.7
0.6
0.5
0.4
0.3
0.2
0.1
0
400 401 402 403 404 405 406 407 408 409 410 411 412 413 414 415 416
Chr 9
r2
Chr 9
r2k

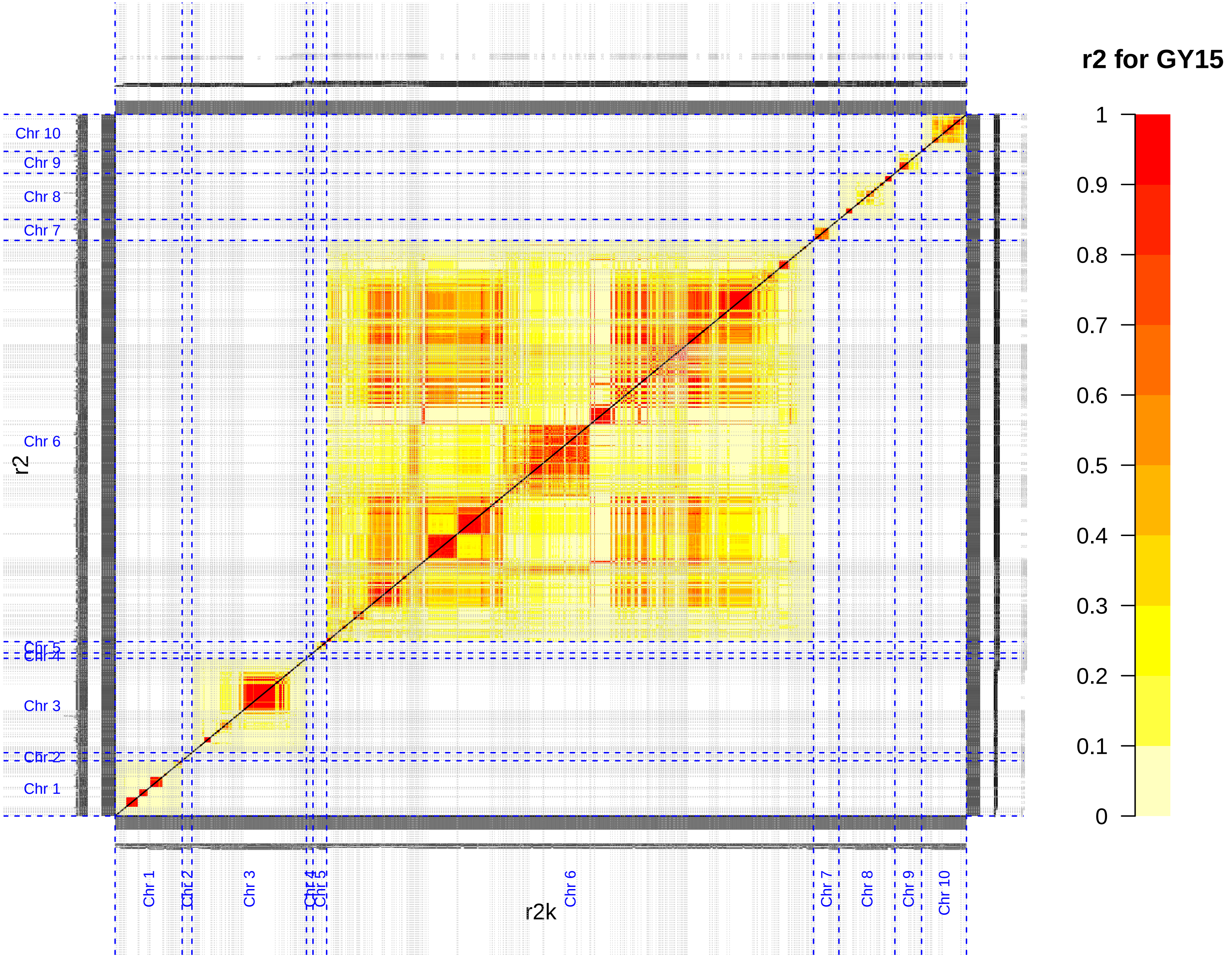
r2 for GY15
1
0.9
0.8
0.7
0.6
0.5
0.4
0.3
0.2
0.1
0
Chr 10
Chr 9
Chr 8
Chr 7
Chr 6
Chr 5
Chr 4
Chr 3
Chr 2
Chr 1
r2
r2k

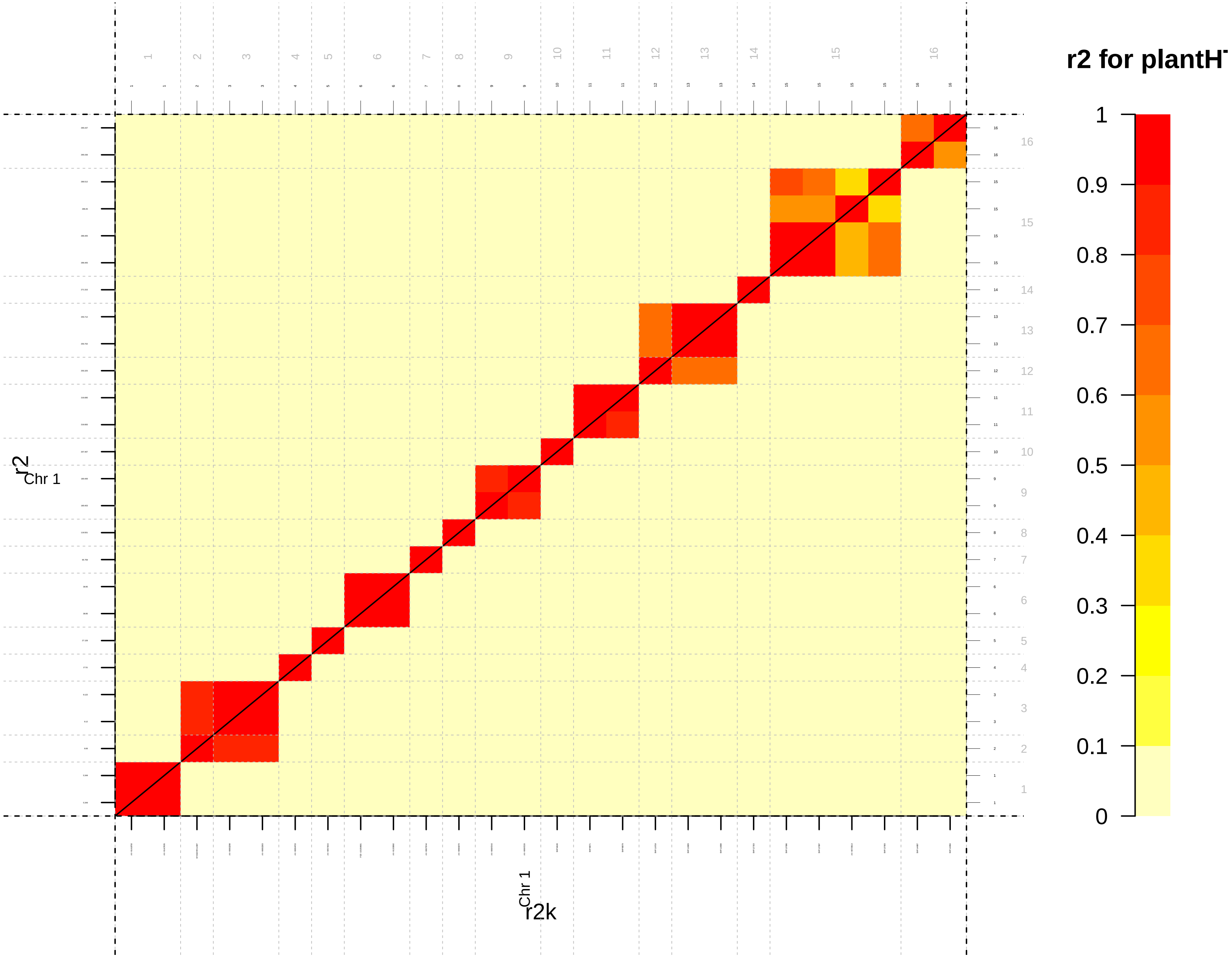
r2 for plantH
1
0.9
0.8
0.7
0.6
0.5
0.4
0.3
0.2
0.1
0
r2
Chr 1
r2k
Chr 1

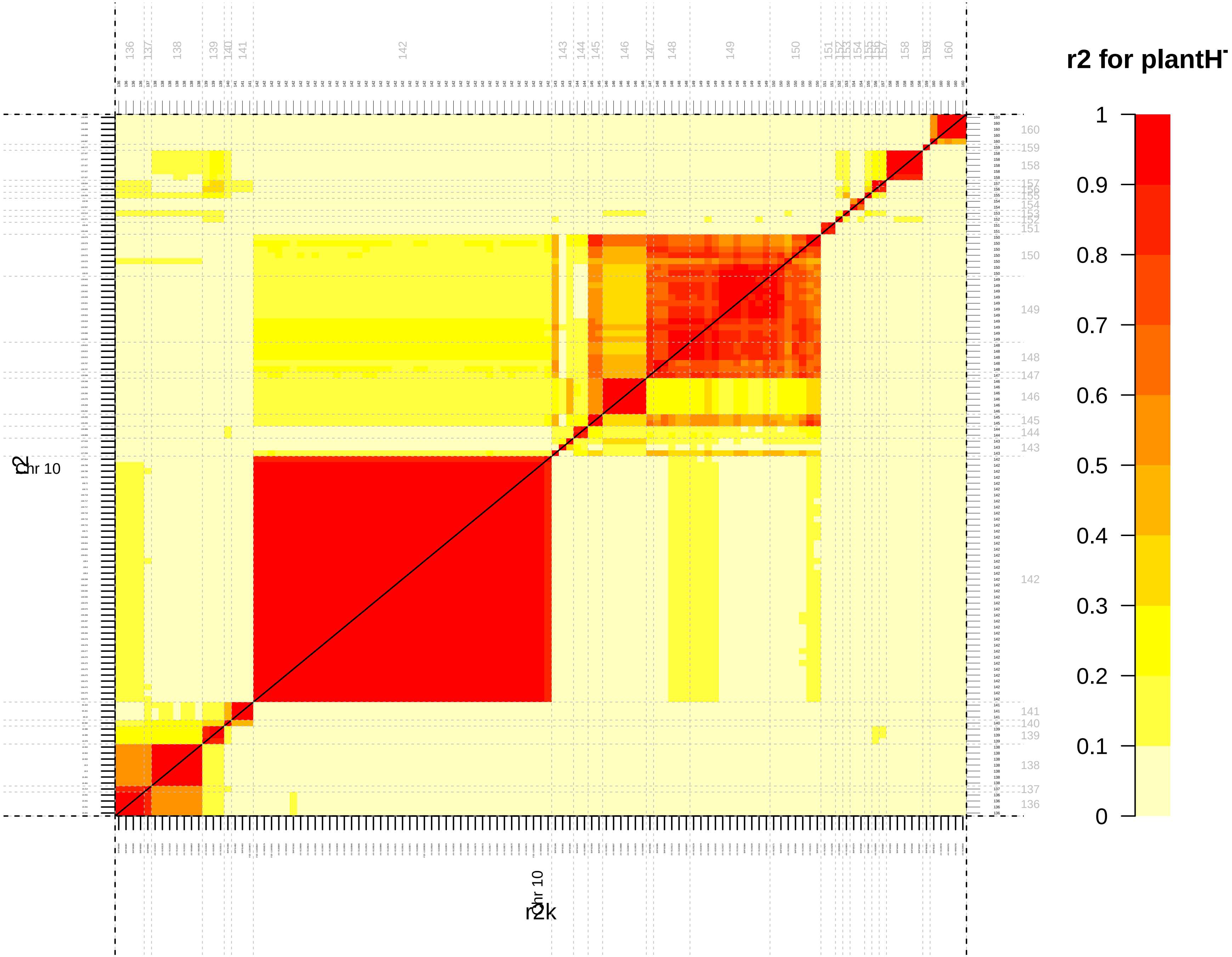
r2 for plantH
1
0.9
0.8
0.7
0.6
0.5
0.4
0.3
0.2
0.1
0
Chr 10
r2
r2k
136
137
138
139
140
141
142
143
144
145
146
147
148
149
150
151
152
153
154
155
156
157
158
159
160

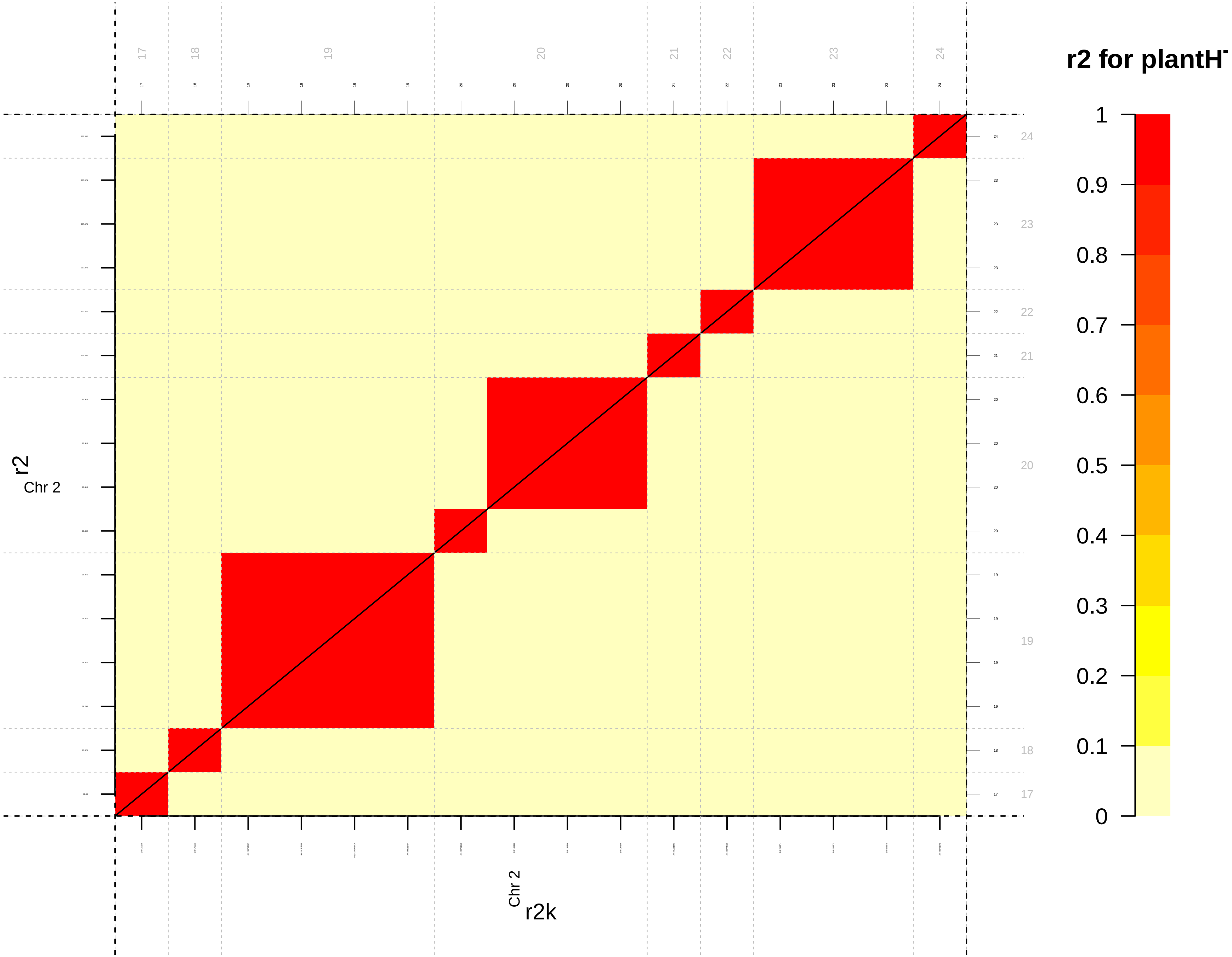
r2 for plantH
1
0.9
0.8
0.7
0.6
0.5
0.4
0.3
0.2
0.1
0
r2
Chr 2
Chr 2
r2k
17
18
19
20
21
22
23
24

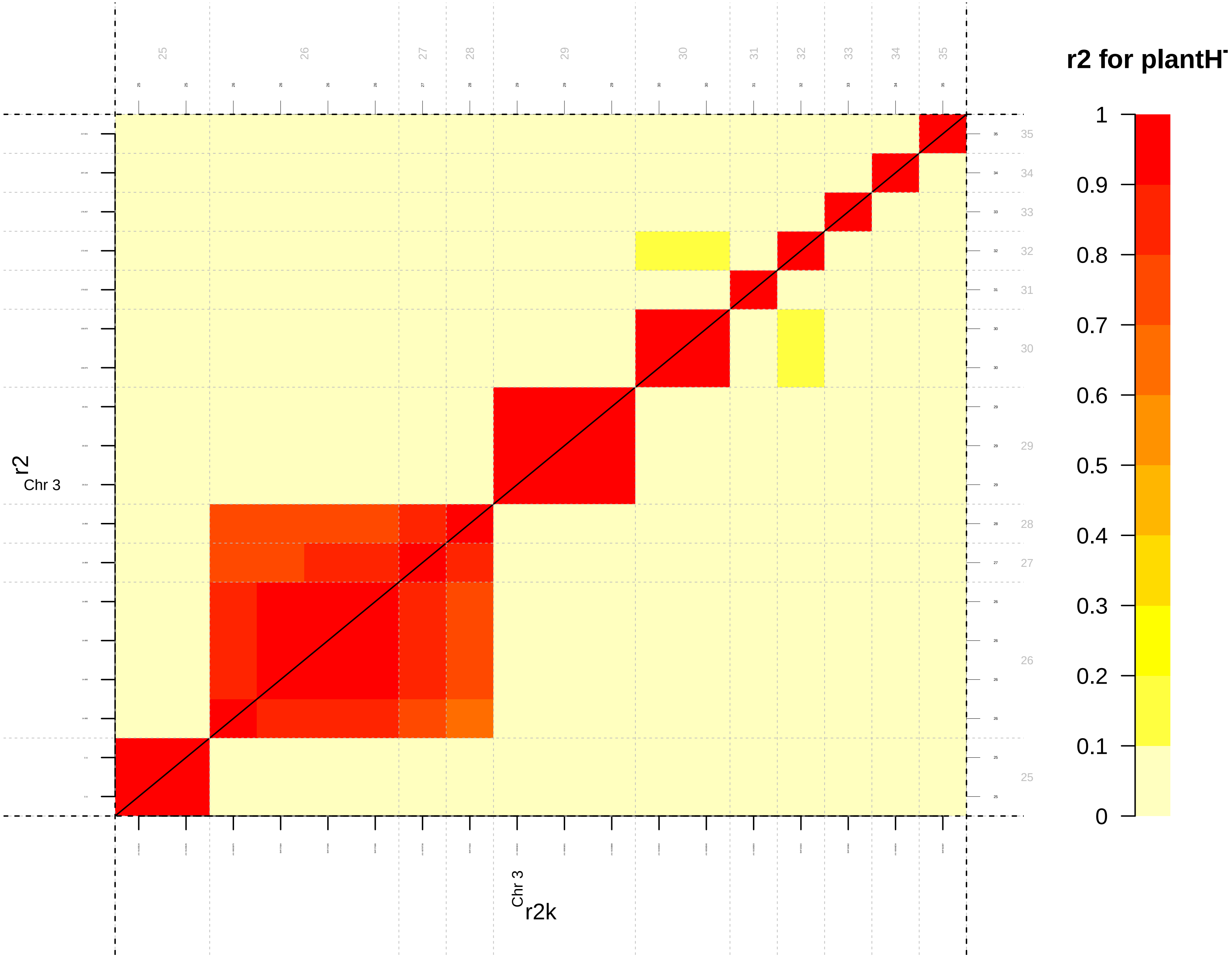

r2 for plantH
1
0.9
0.8
0.7
0.6
0.5
0.4
0.3
0.2
0.1
0
r2
Chr 3
Chr 3
r2k
25
26
27
28
29
30
31
32
33
34
35

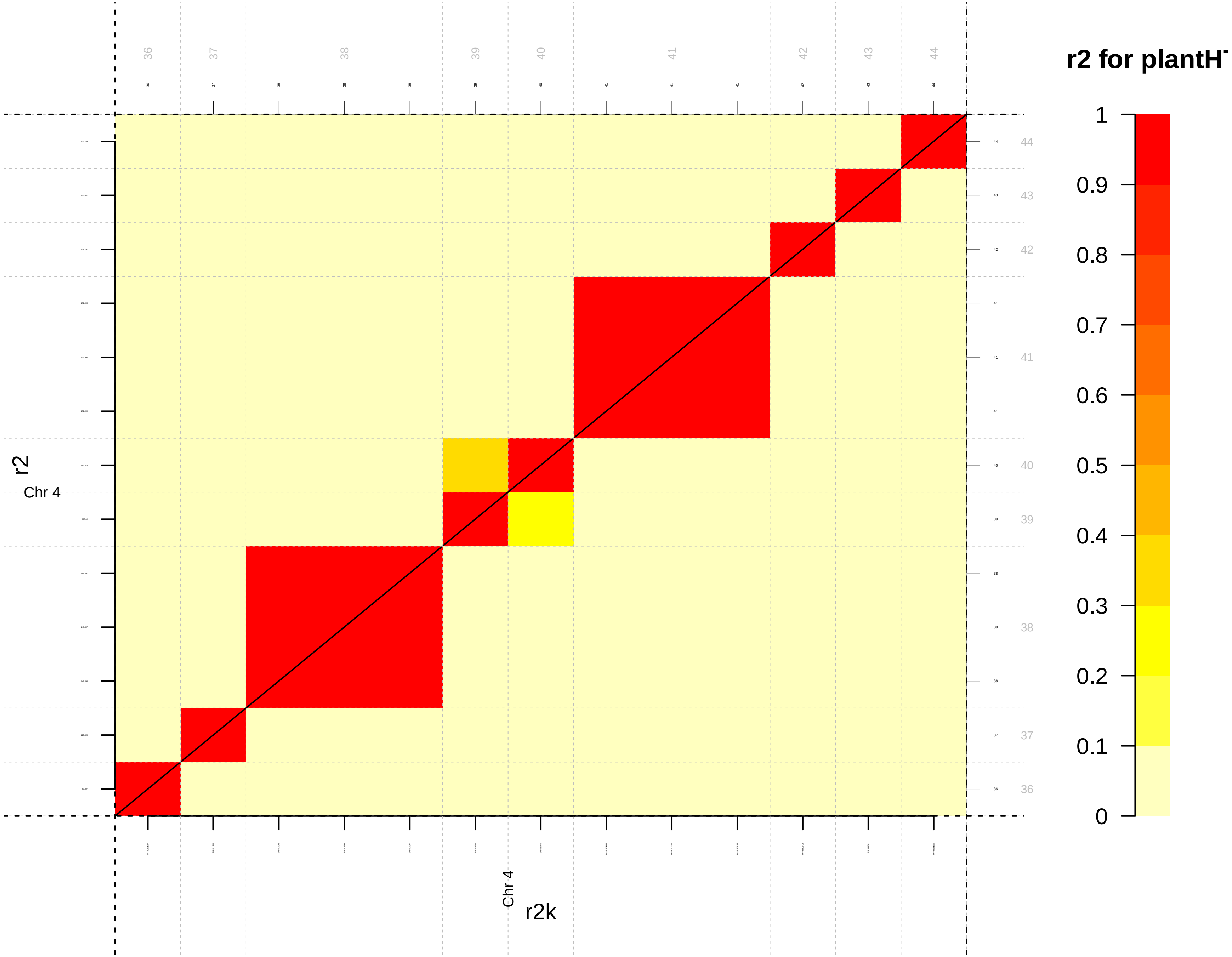

r2 for plantH
1
0.9
0.8
0.7
0.6
0.5
0.4
0.3
0.2
0.1
0
36
37
38
39
40
41
42
43
44
r2
Chr 4
r2k
Chr 4

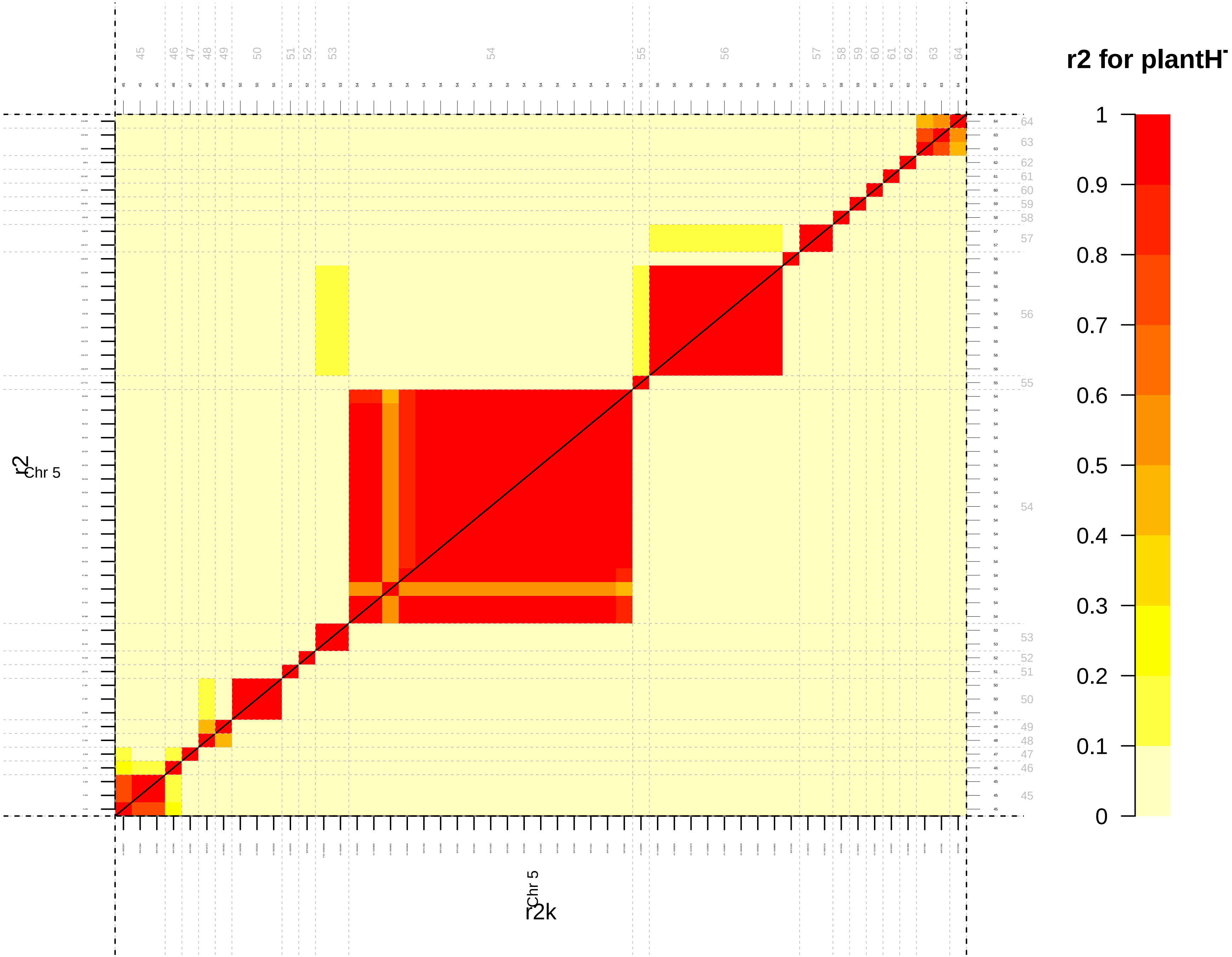
r2 for plantH
1
0.9
0.8
0.7
0.6
0.5
0.4
0.3
0.2
0.1
0
r2
Chr 5
r2k
Chr 5
45
46
47
48
49
50
51
52
53
54
55
56
57
58
59
60
61
62
63
64

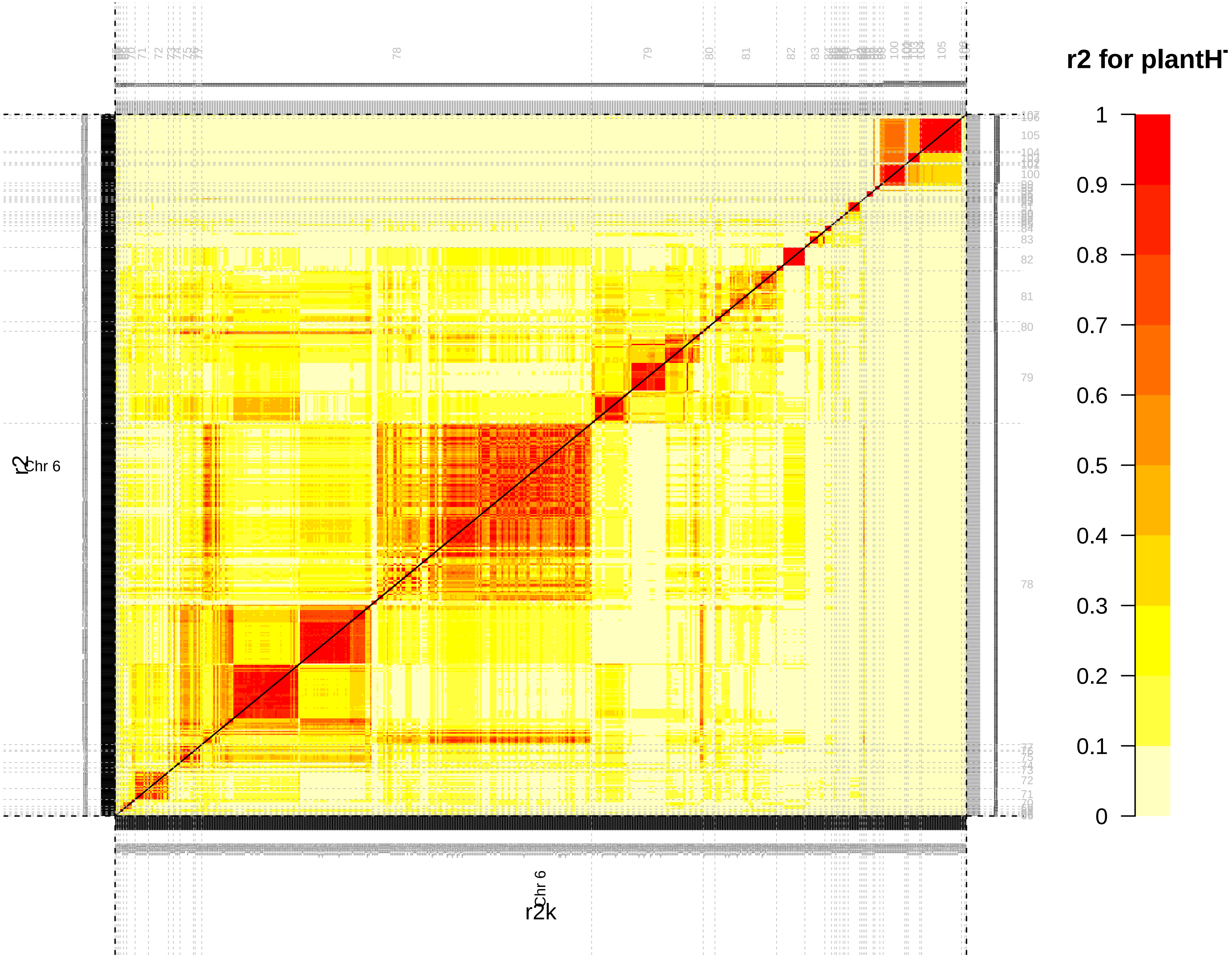
r2 for plantH
1
0.9
0.8
0.7
0.6
0.5
0.4
0.3
0.2
0.1
0
Chr 6
r2
Chr 6
r2k

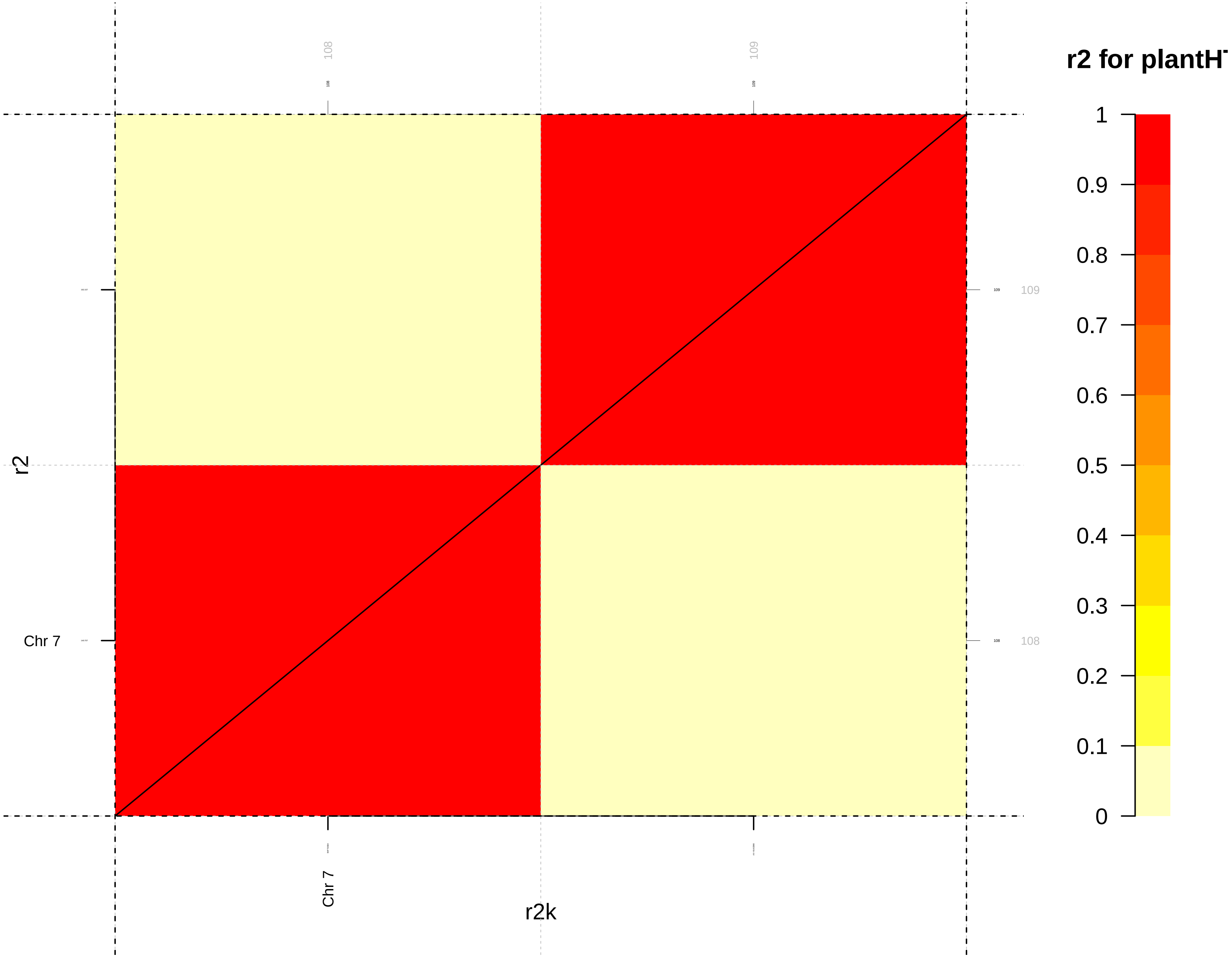

r2 for plantH
1
0.9
0.8
0.7
0.6
0.5
0.4
0.3
0.2
0.1
0
108
109
r2
Chr 7
r2k

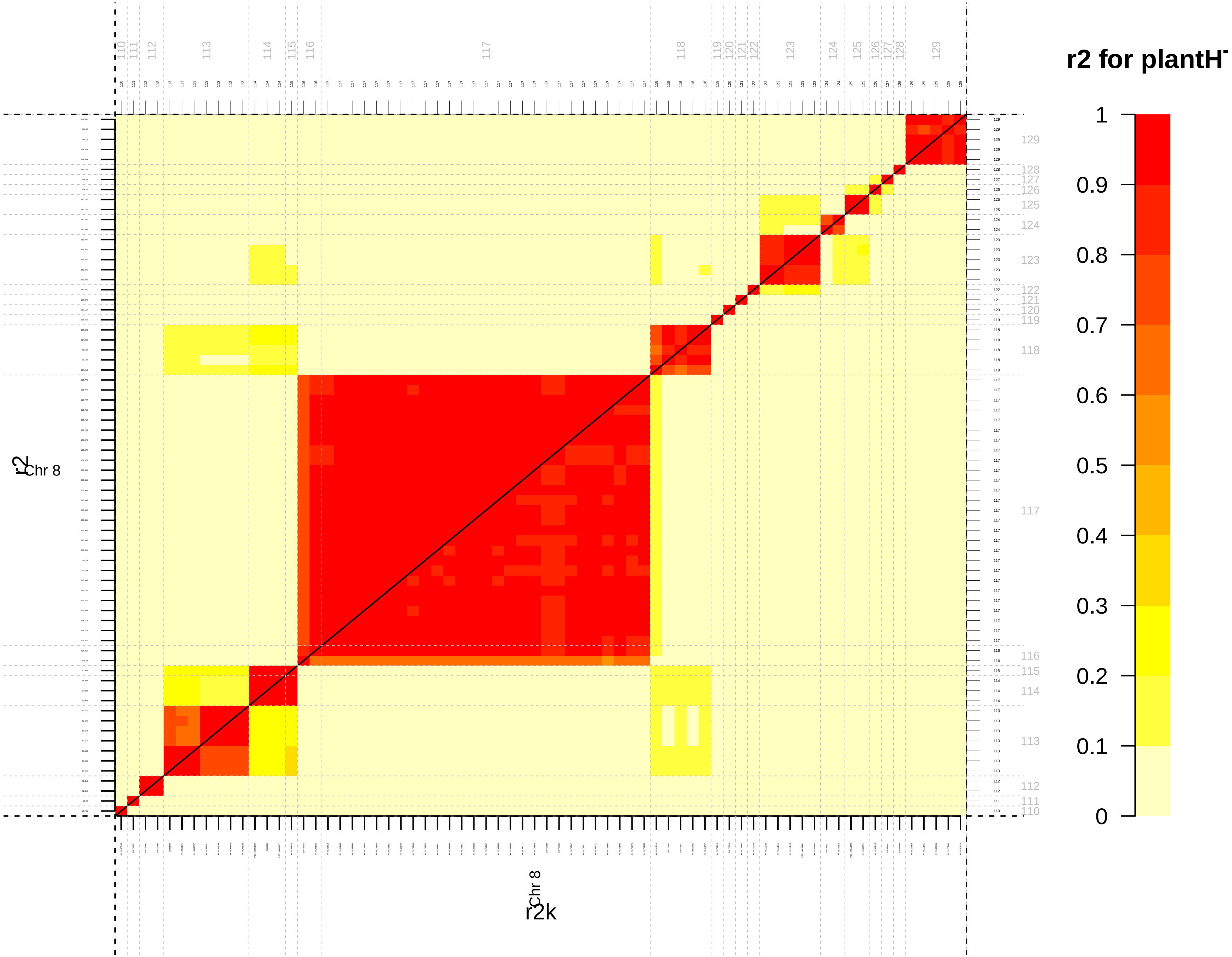
r2 for plantH
1
0.9
0.8
0.7
0.6
0.5
0.4
0.3
0.2
0.1
0
Chr 8
r2
Chr 8
r2k
110
111
112
113
114
115
116
117
118
119
120
121
122
123
124
125
126
127
128
129

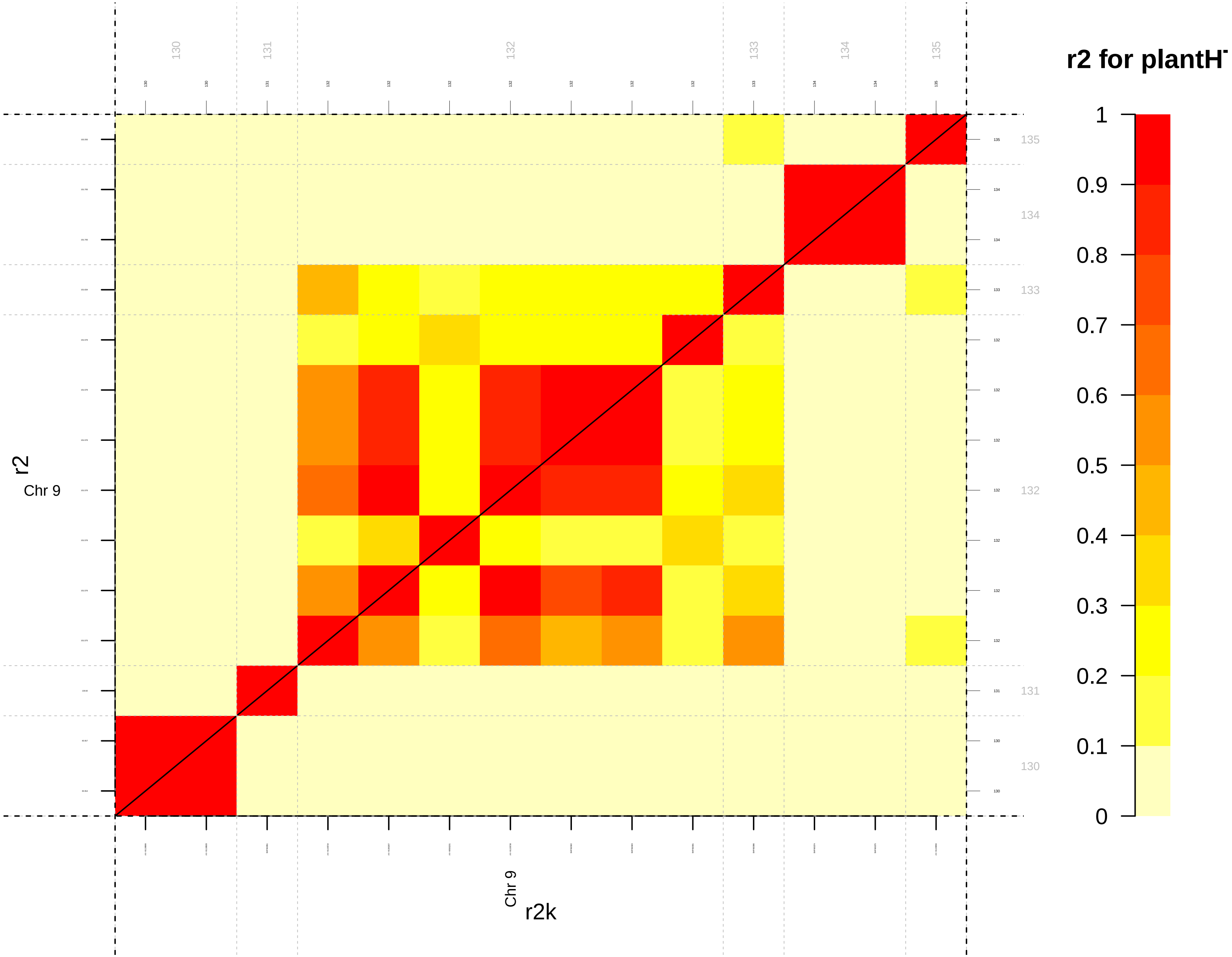
r2 for plantH
1
0.9
0.8
0.7
0.6
0.5
0.4
0.3
0.2
0.1
0
130
131
132
133
134
135
r2
Chr 9
Chr 9
r2k

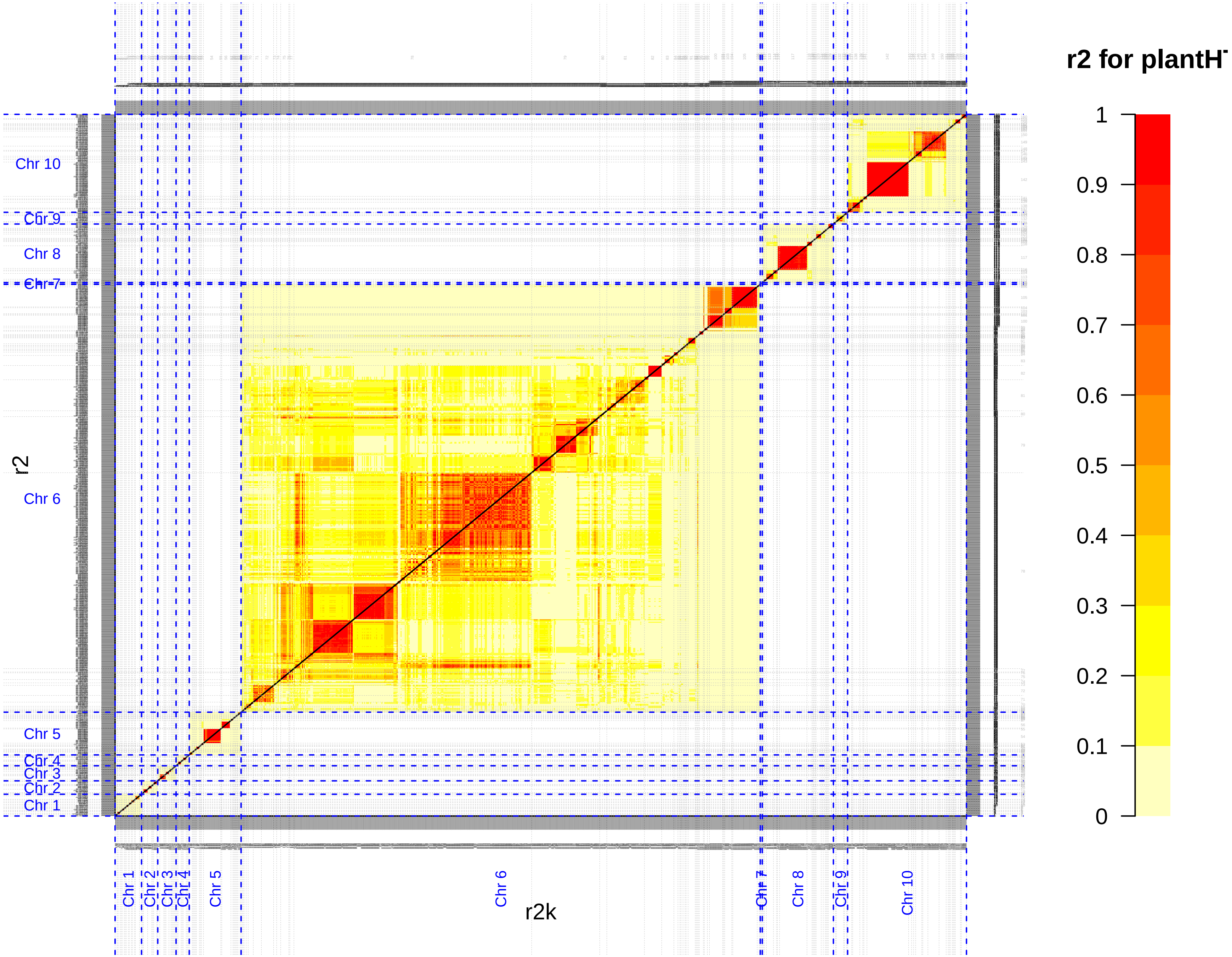
r2 for plantH
1
0.9
0.8
0.7
0.6
0.5
0.4
0.3
0.2
0.1
0
Chr 10
Chr 9
Chr 8
Chr 7
Chr 6
Chr 5
Chr 4
Chr 3
Chr 2
Chr 1
r2
r2k

Additional file 7 (.pdf):

Figure S7: QTL limits obtained by the *LD_Adj* approach projected on heatmaps representing the level of LD between associated SNPs for each trait (DTA: male flowering time, plantHT: plant height and GY: grain yield) for each chromosome. Upper and lower triangles on the heatmaps represent the r^2 and r^2K values between associated SNPs, respectively. Linkage disequilibrium between loci was colored according to values from weak LD (yellow) to high LD (red). The significant markers were ordered according to their physical positions on the chromosome and were represented by ticks on the four sides of the heatmaps. Limits of QTLs were displayed by gray dotted lines. QTL numbers were indicated in gray on the top and the right of each heatmap.
