# Supplementary material for: Genotyping-by-sequencing and SNP-arrays are complementary for detecting quantitative trait loci by tagging different haplotypes in association studies
Source: BMC Plant Biol. 2019 Jul 16;19:318. doi: 10.1186/s12870-019-1926-4 (PMC6636005; doi:10.1186/s12870-019-1926-4)
Supplement: Supplementary file 8 — Figure S8. QTL limits obtained by the LD_win approach projected on heatmaps representing the level of LD between associated SNPs for each trait (DTA: male flowering time, plantHT: plant height and GY: grain yield) and each chromosome. Upper and lower triangles on the heatmaps represented the r2 and r2K values between associated SNPs, respectively. Linkage disequilibrium between loci was colored according to values from weak LD (yellow) to high LD (red). The significant markers were ordered according to their physical positions on the chromosome and were represented by ticks on the four sides of the heatmaps. Limits of QTLs were displayed by gray dotted lines. QTL numbers were indicated in gray on the top and the right of each heatmap. (PDF 7360 kb) [file 12870_2019_1926_MOESM8_ESM.pdf]

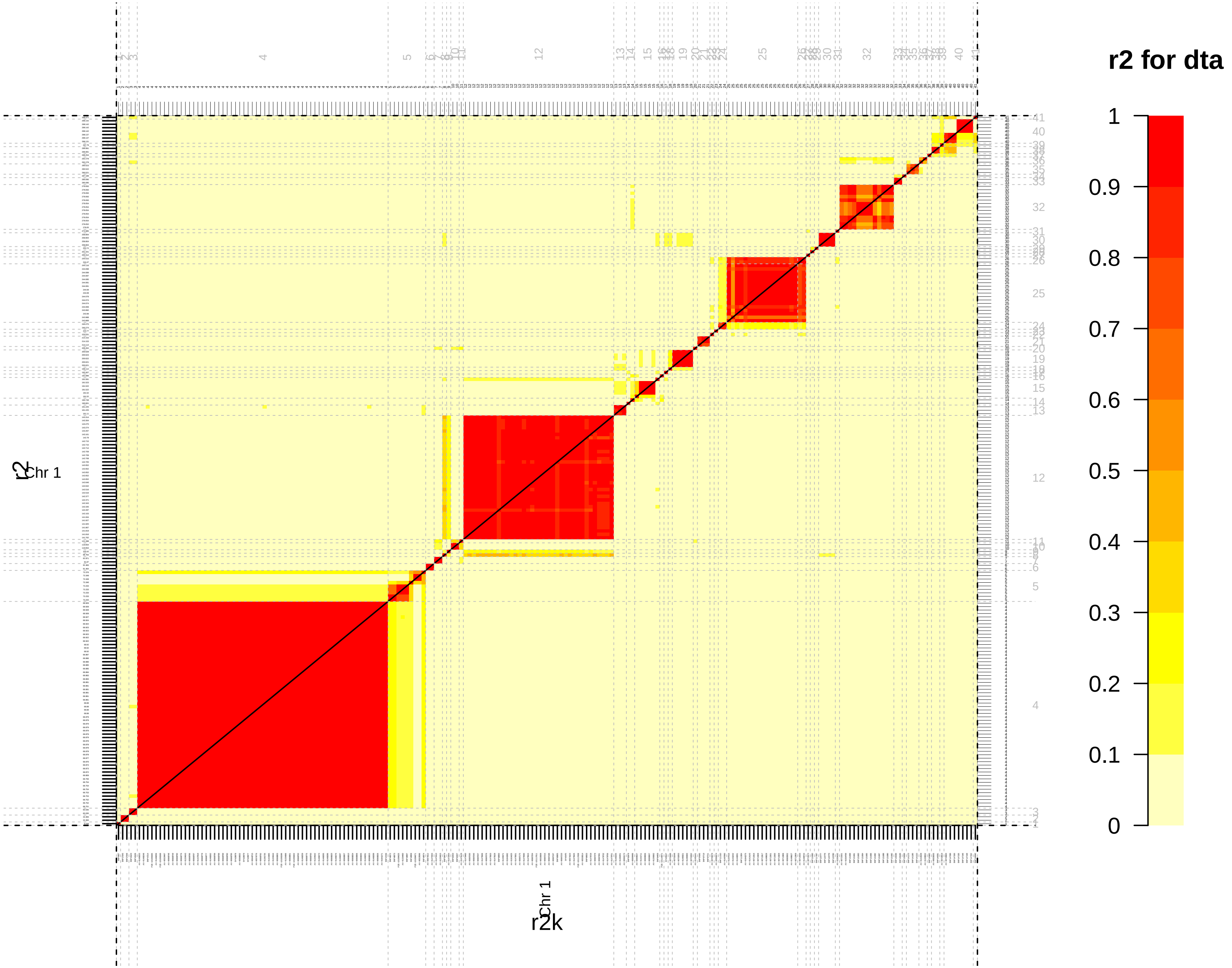

r2 for dta
1
0.9
0.8
0.7
0.6
0.5
0.4
0.3
0.2
0.1
0
Chr 1
r2k
r2

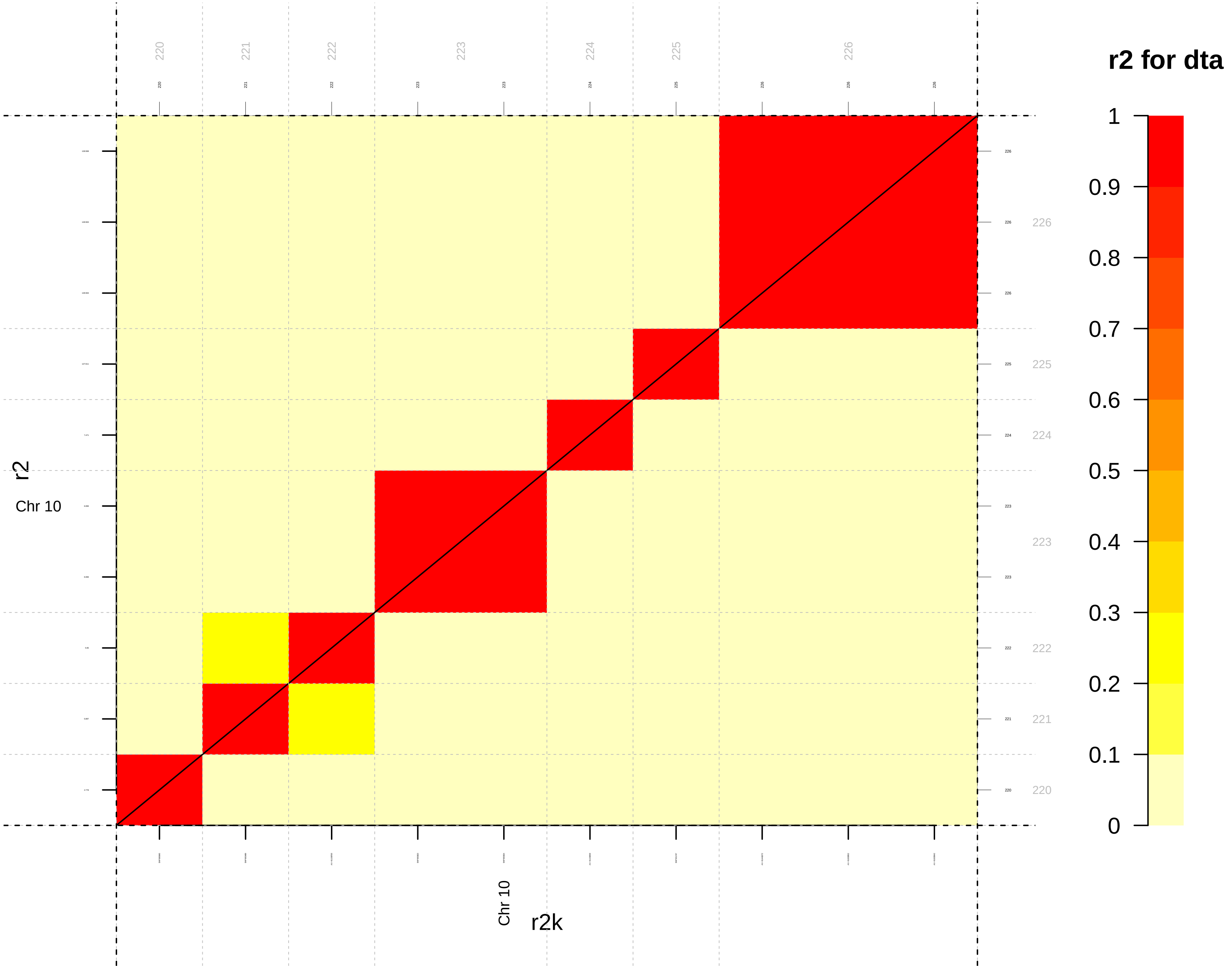
r2 for dta
1
0.9
0.8
0.7
0.6
0.5
0.4
0.3
0.2
0.1
0
220
221
222
223
224
225
226
r2
Chr 10
Chr 10
r2k

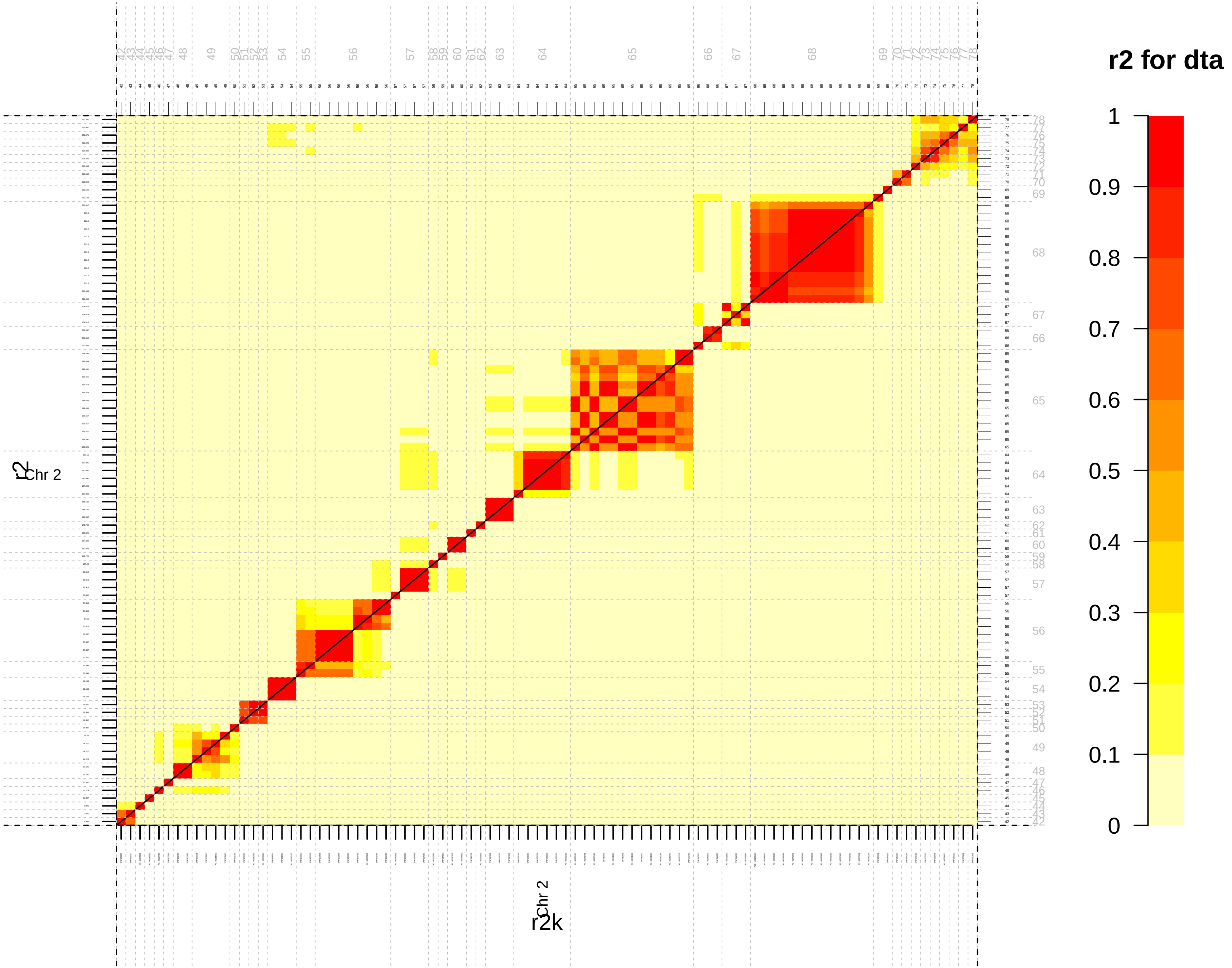
r2 for dta
1
0.9
0.8
0.7
0.6
0.5
0.4
0.3
0.2
0.1
0
Chr 2
r2
Chr 2
r2k

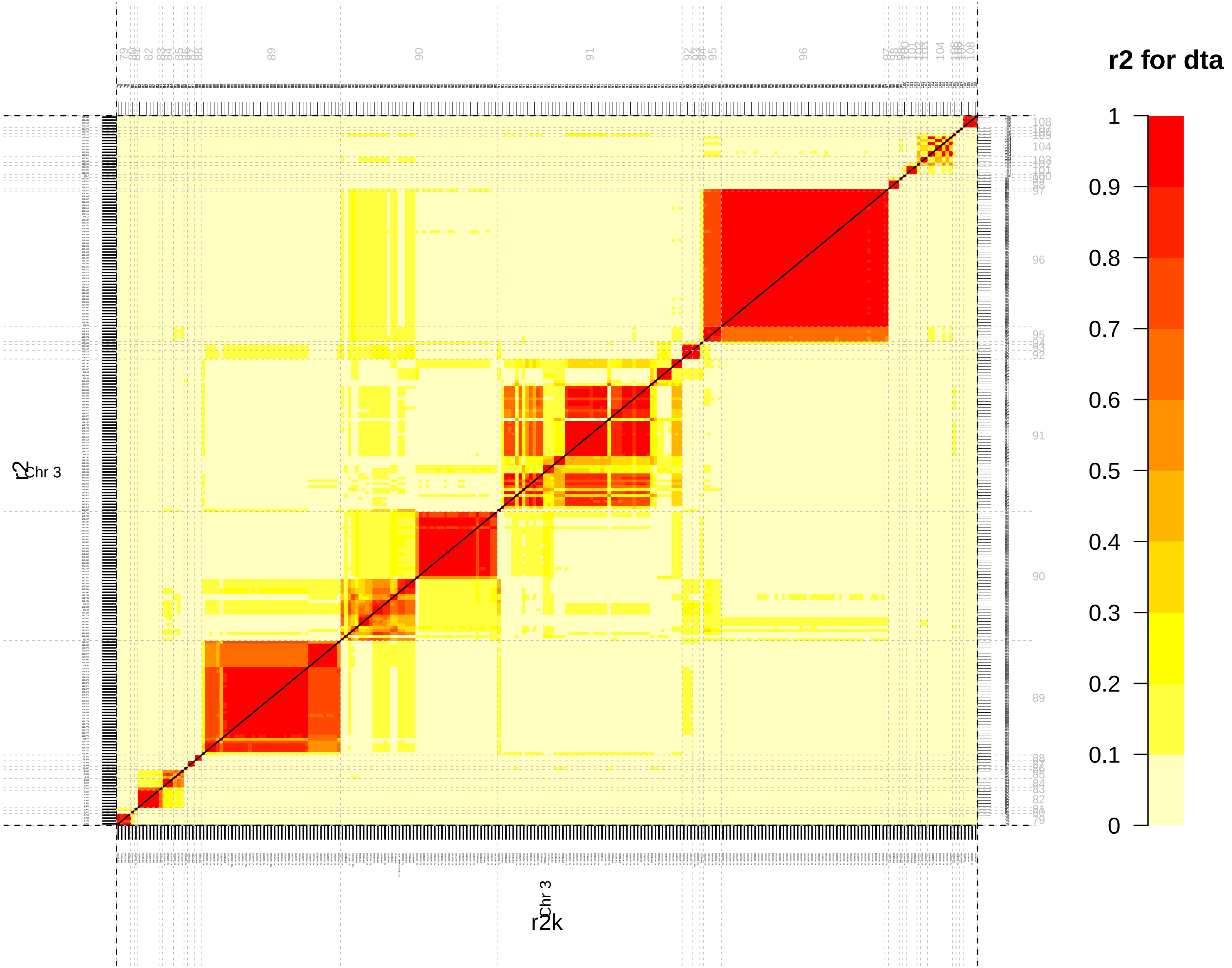
r2 for dta
1
0.9
0.8
0.7
0.6
0.5
0.4
0.3
0.2
0.1
0
Chr 3
r2
Chr 3
r2k

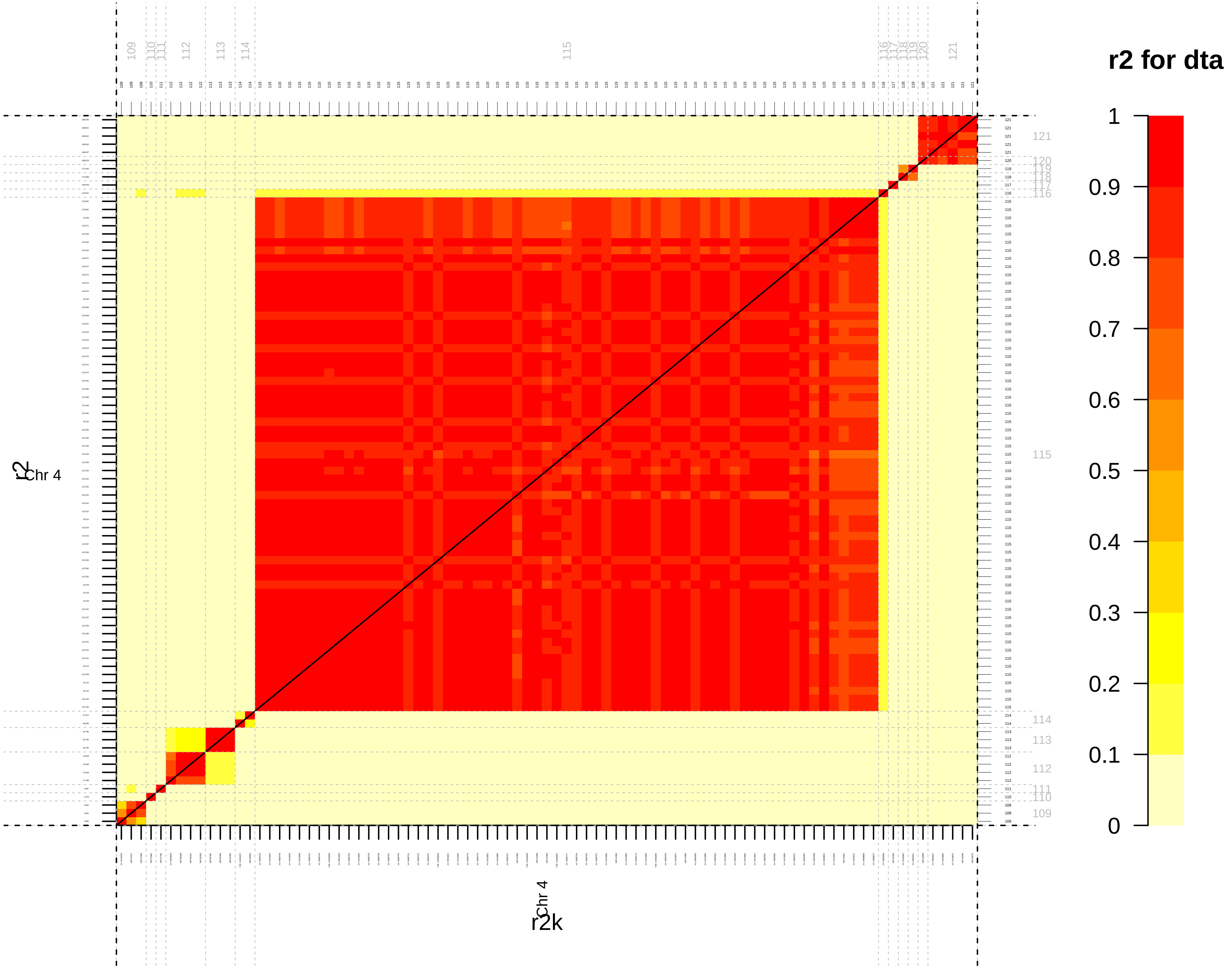

r2 for dta
1
0.9
0.8
0.7
0.6
0.5
0.4
0.3
0.2
0.1
0
r2
Chr 4
r2k
Chr 4
109
110
111
112
113
114
115
116
117
118
119
120
121

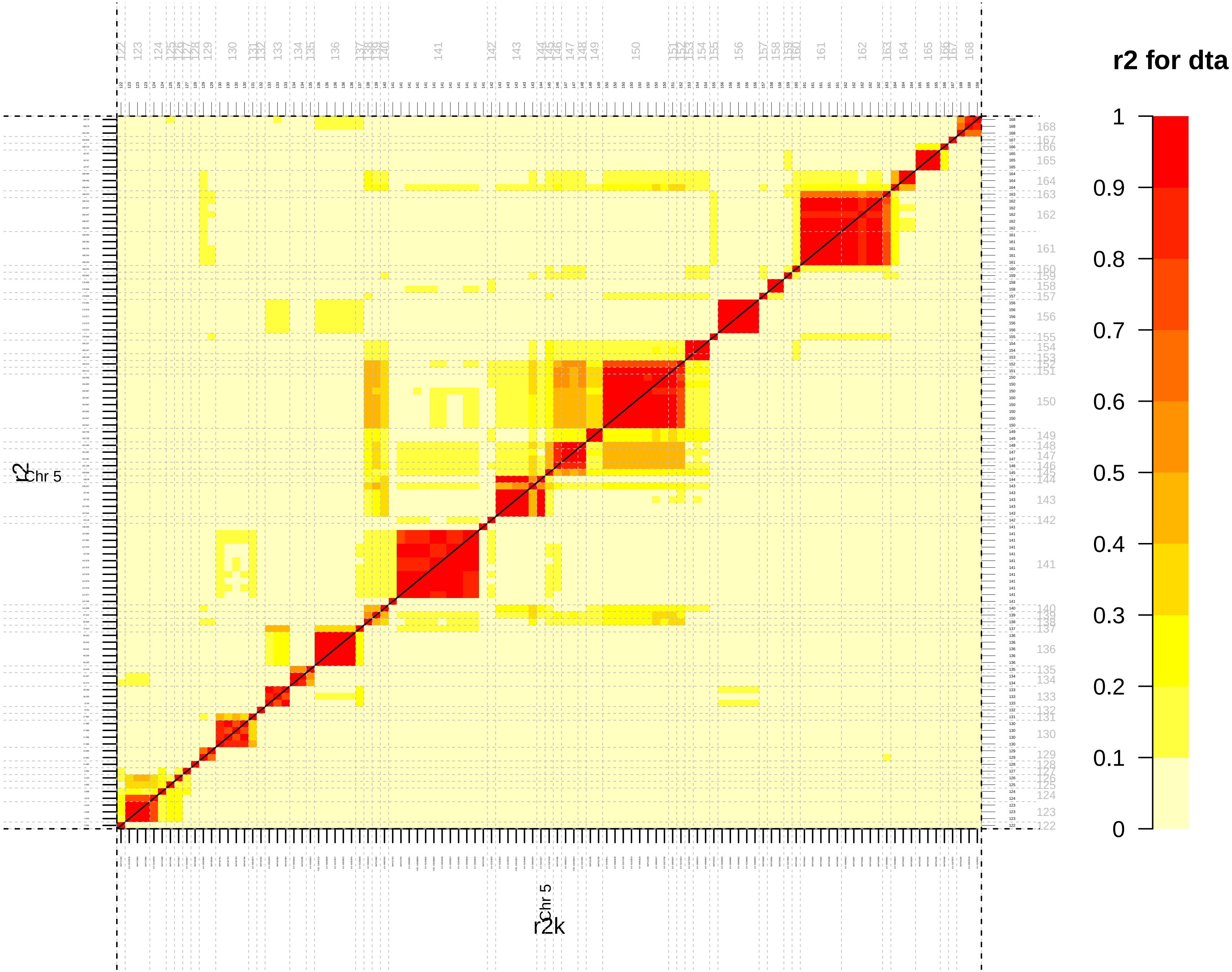
r2 for dta
1
0.9
0.8
0.7
0.6
0.5
0.4
0.3
0.2
0.1
0
Chr 5
r2
Chr 5
r2k

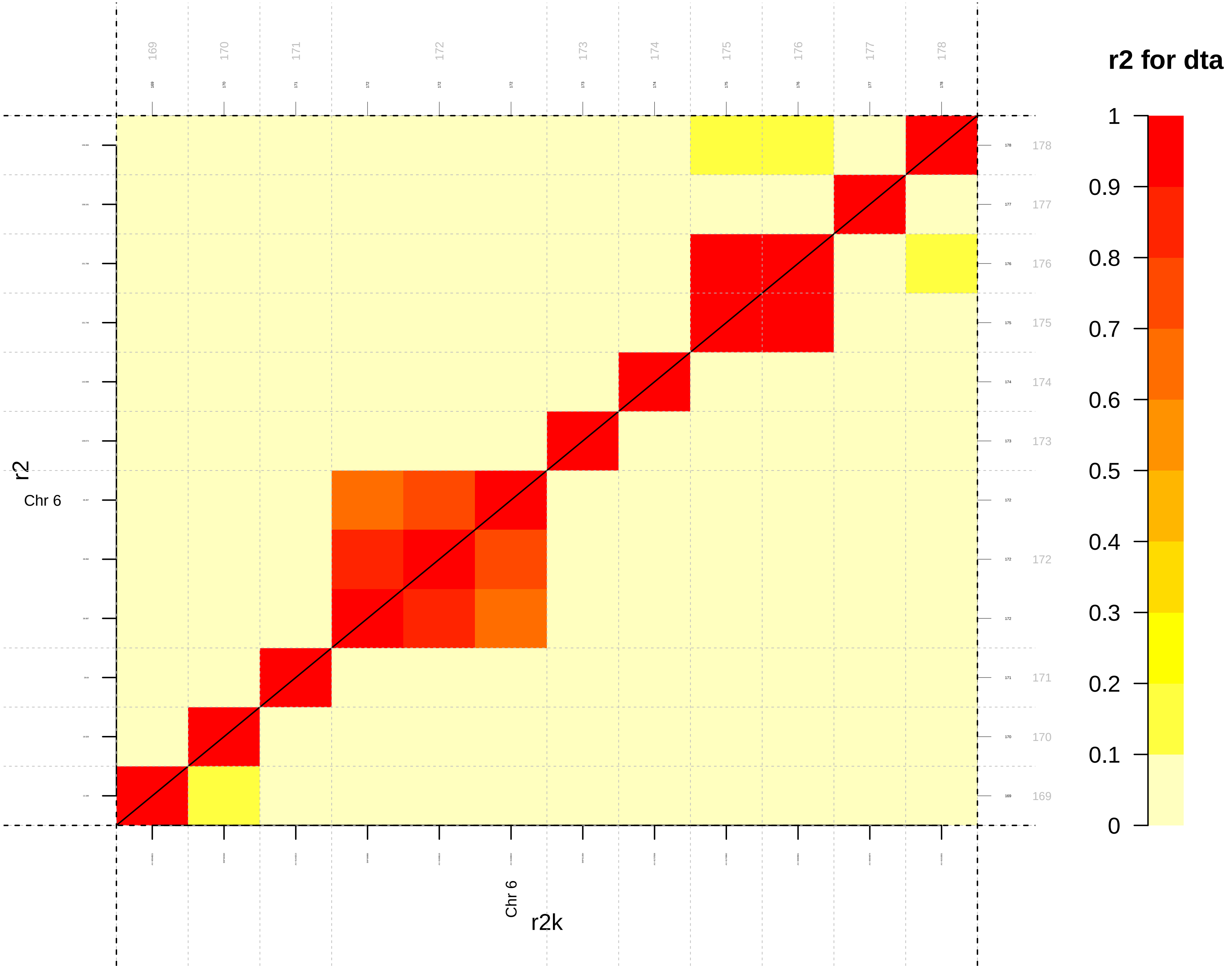
r2 for dta
1
0.9
0.8
0.7
0.6
0.5
0.4
0.3
0.2
0.1
0
169
170
171
172
173
174
175
176
177
178
r2
Chr 6
r2k

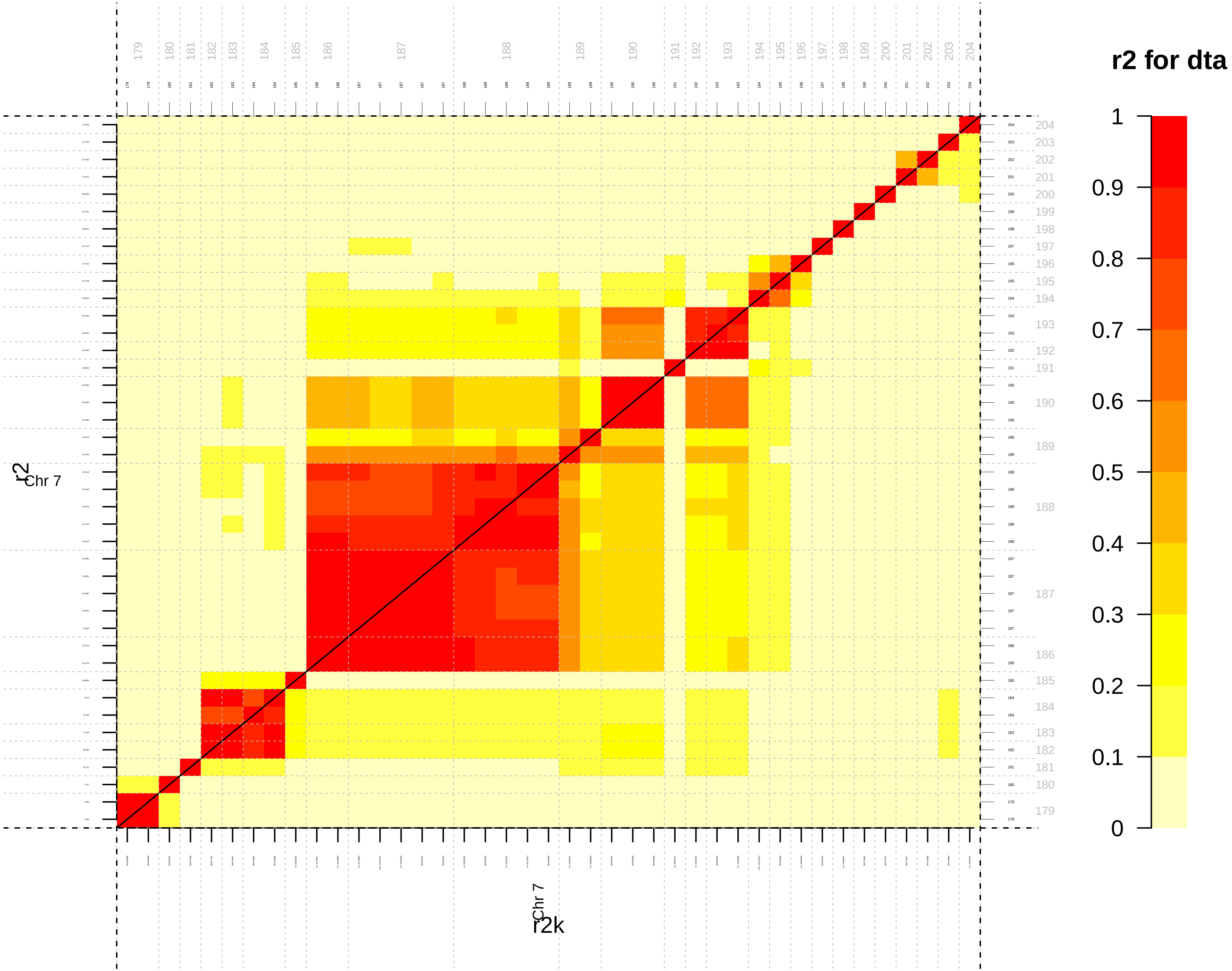
r2 for dta
1
0.9
0.8
0.7
0.6
0.5
0.4
0.3
0.2
0.1
0
179 180 181 182 183 184 185 186 187 188 189 190 191 192 193 194 195 196 197 198 199 200 201 202 203 204
204 203 202 201 200 199 198 197 196 195 194 193 192 191 190 189 188 187 186 185 184 183 182 181 180 179
r2
Chr 7
Chr 7
r2k

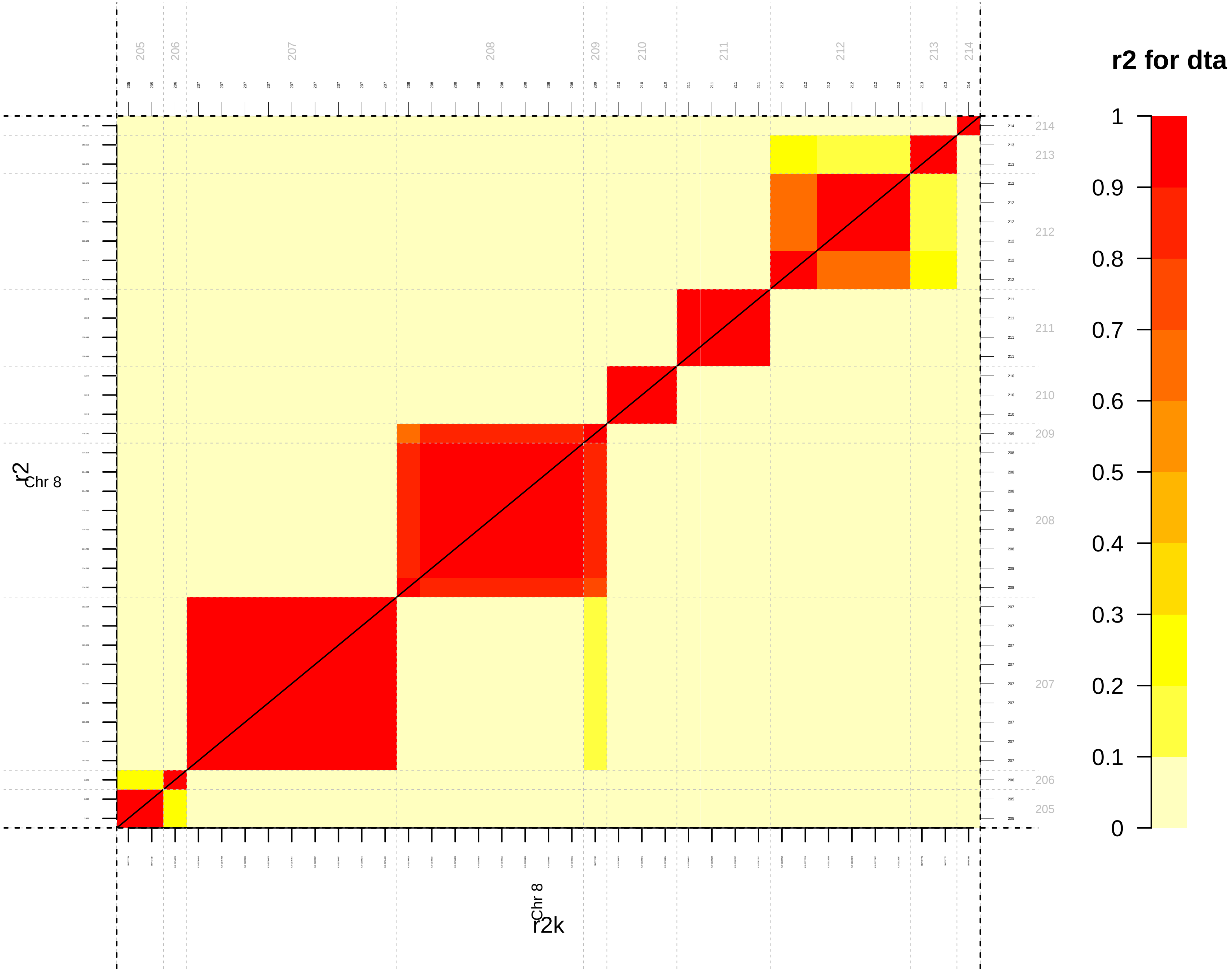
r2 for dta
1
0.9
0.8
0.7
0.6
0.5
0.4
0.3
0.2
0.1
0
205
206
207
208
209
210
211
212
213
214
r2
Chr 8
r2k
Chr 8

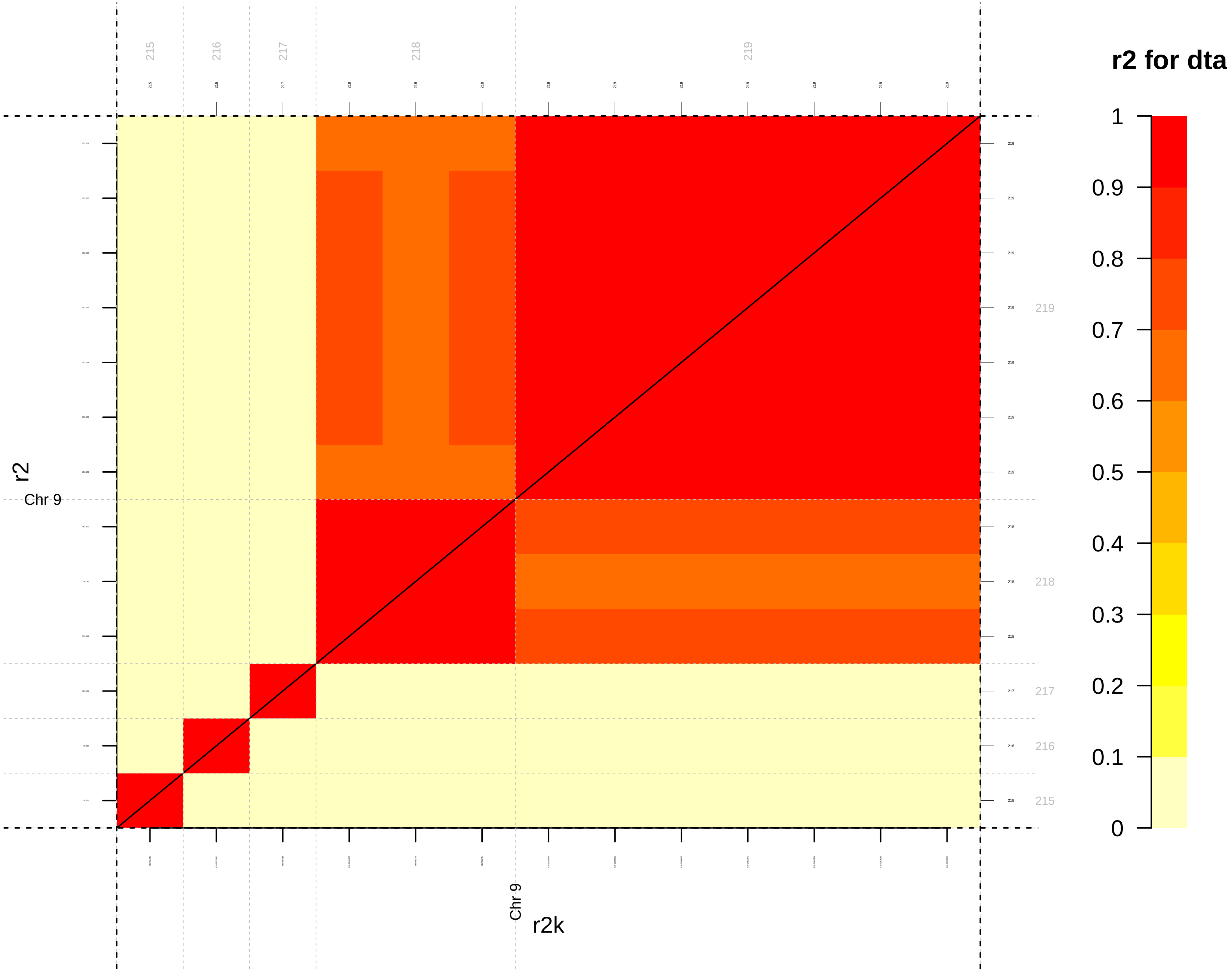
r2 for dta
1
0.9
0.8
0.7
0.6
0.5
0.4
0.3
0.2
0.1
0
215
216
217
218
219
r2
Chr 9
r2k

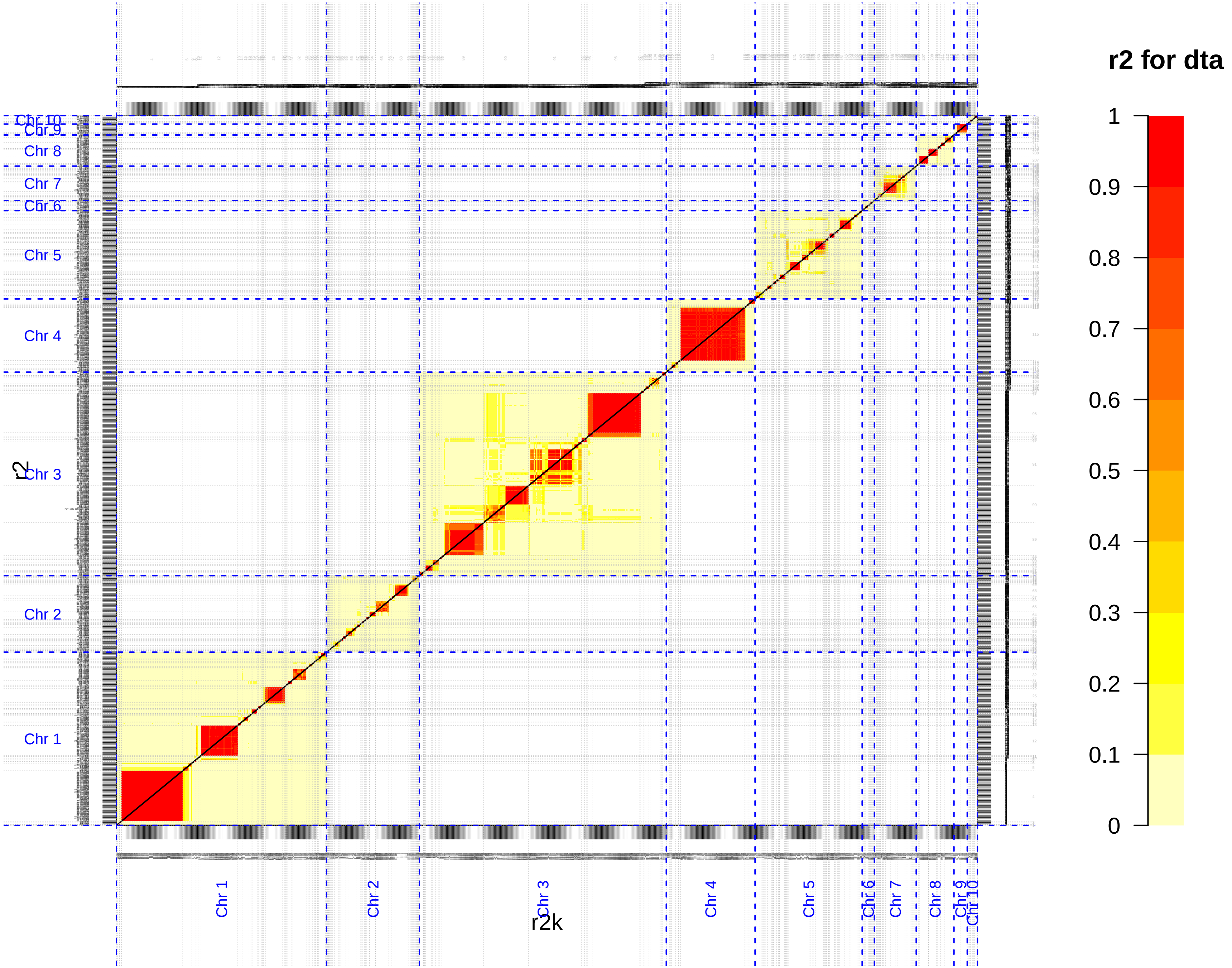

r2 for dta
1
0.9
0.8
0.7
0.6
0.5
0.4
0.3
0.2
0.1
0
Chr 10
Chr 9
Chr 8
Chr 7
Chr 6
Chr 5
Chr 4
Chr 3
Chr 2
Chr 1
r2
r2k

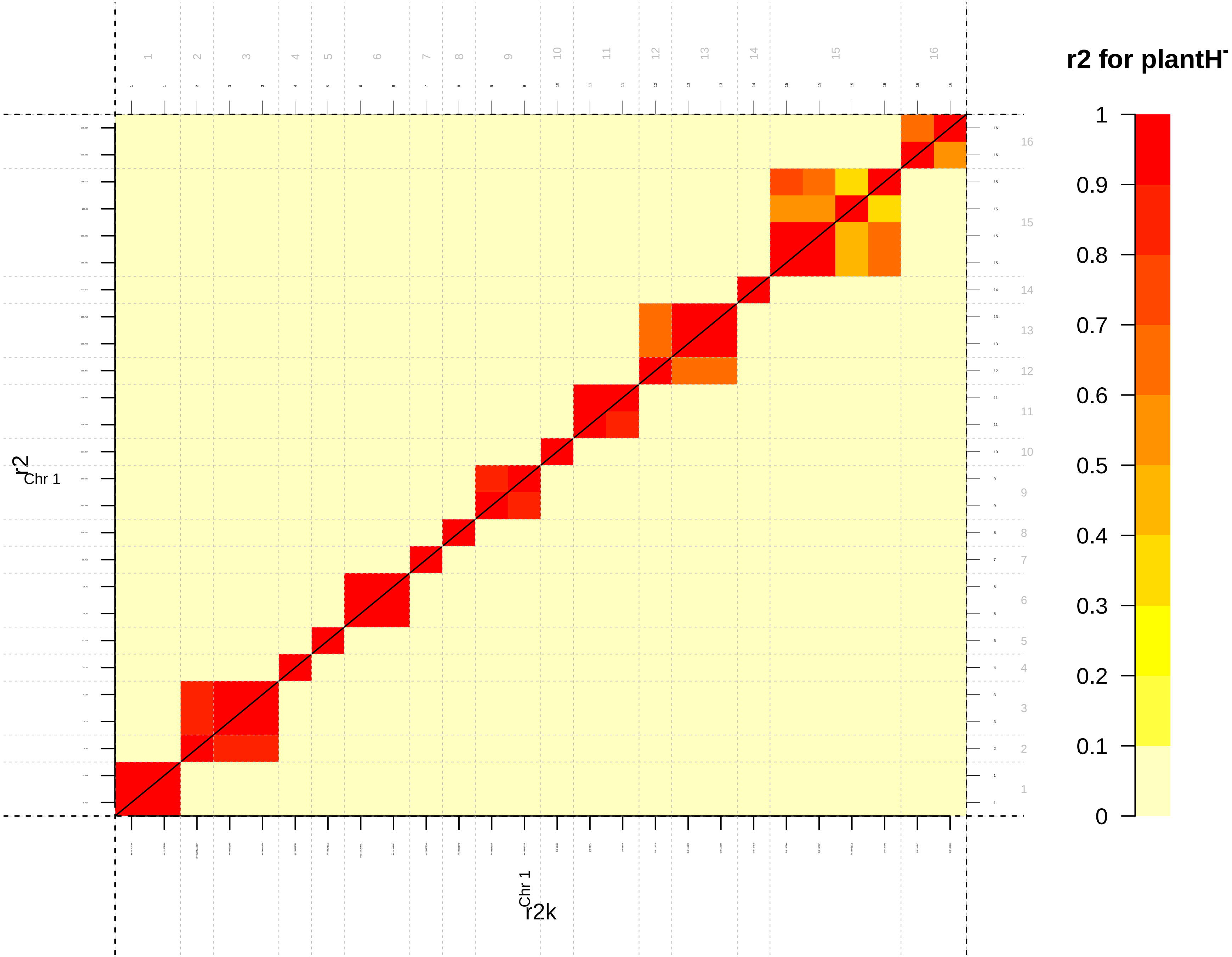

r2 for plantH
1
0.9
0.8
0.7
0.6
0.5
0.4
0.3
0.2
0.1
0
r2
Chr 1
r2k
Chr 1

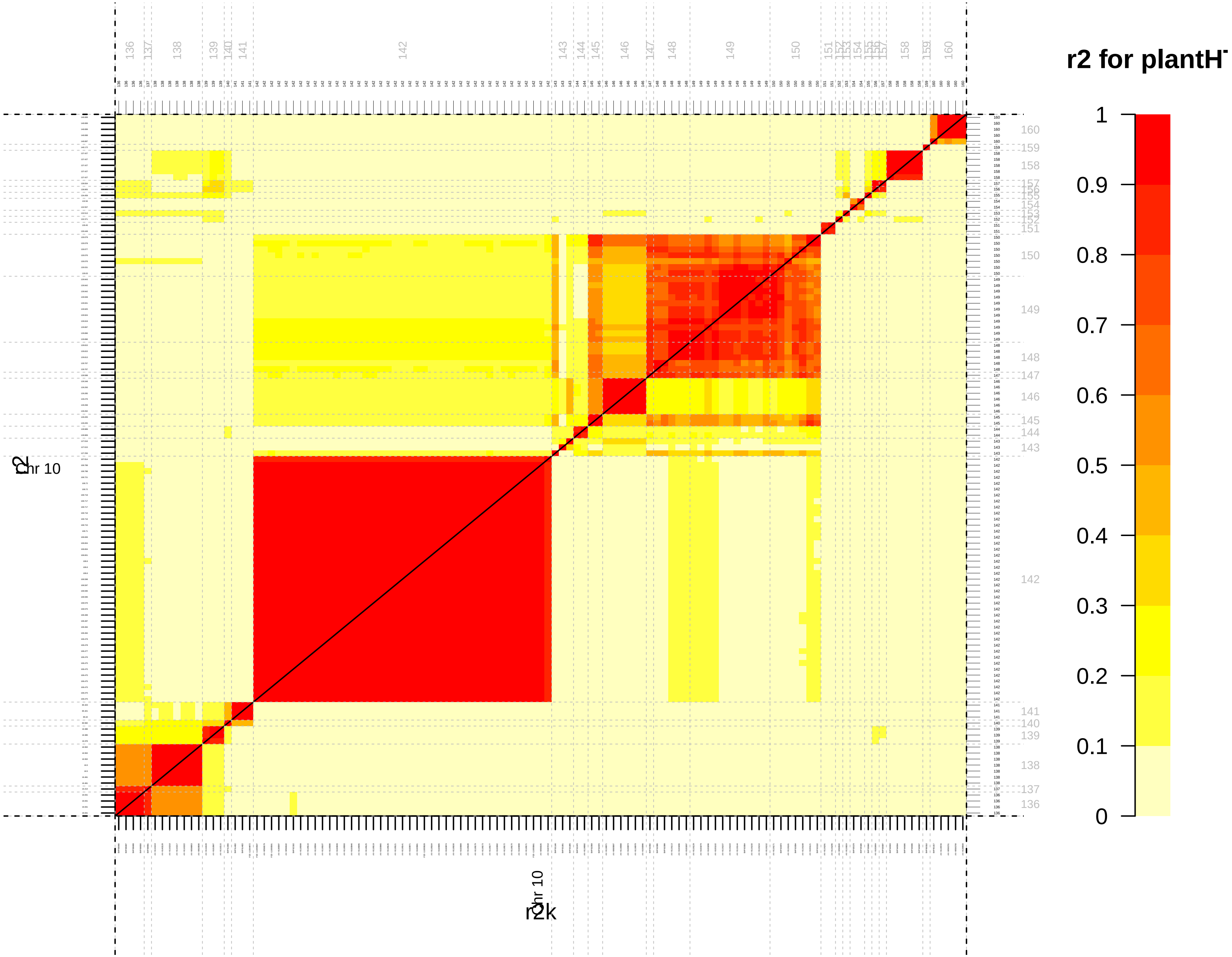
r2 for plantH
1
0.9
0.8
0.7
0.6
0.5
0.4
0.3
0.2
0.1
0
Chr 10
r2
r2k

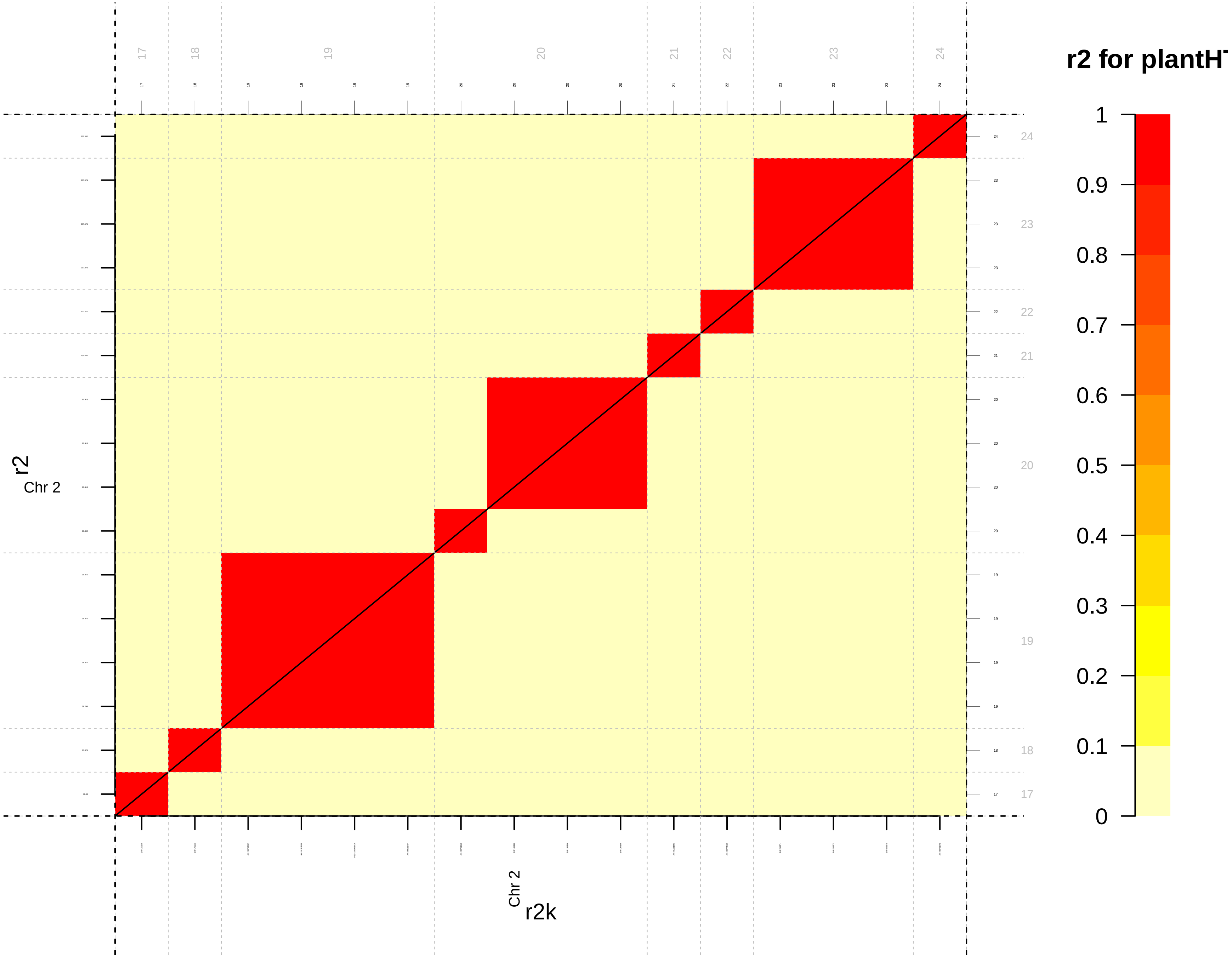
r2 for plantH
1
0.9
0.8
0.7
0.6
0.5
0.4
0.3
0.2
0.1
0
r2
Chr 2
r2k
Chr 2
17
18
19
20
21
22
23
24

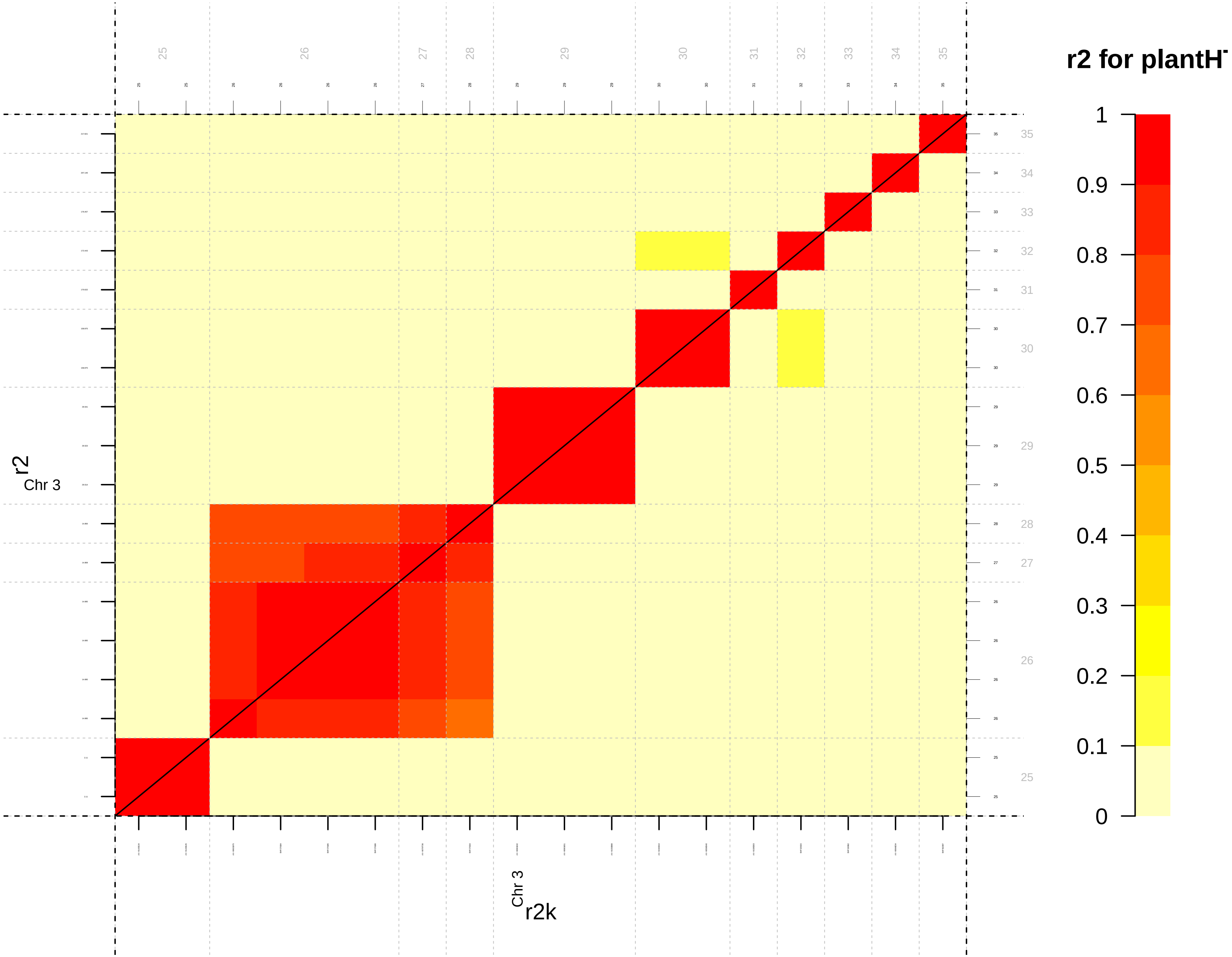

r2 for plantH
1
0.9
0.8
0.7
0.6
0.5
0.4
0.3
0.2
0.1
0
r2
Chr 3
r2k
Chr 3
25
26
27
28
29
30
31
32
33
34
35

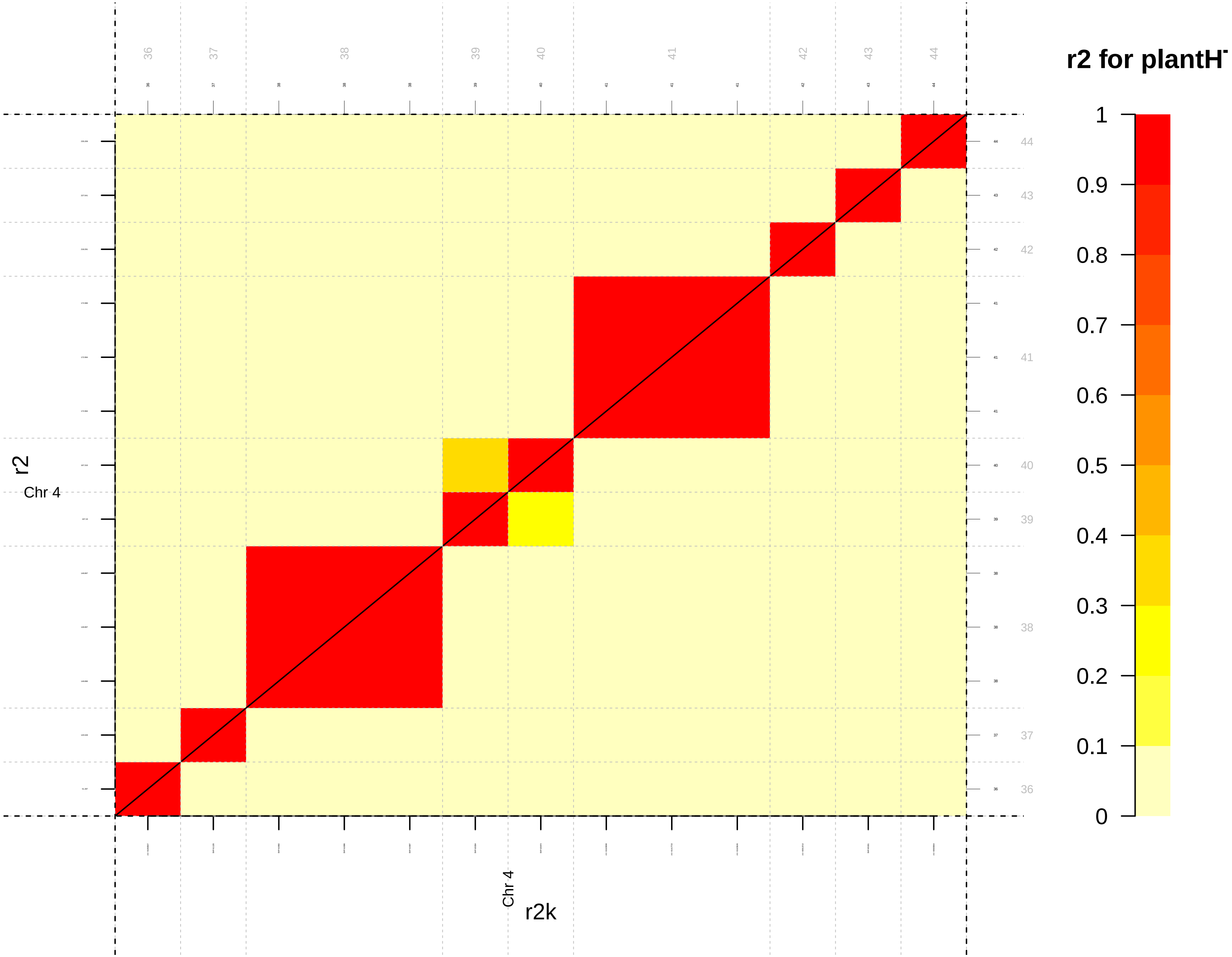
r2 for plantH
1
0.9
0.8
0.7
0.6
0.5
0.4
0.3
0.2
0.1
0
36
37
38
39
40
41
42
43
44
r2
Chr 4
r2k
Chr 4

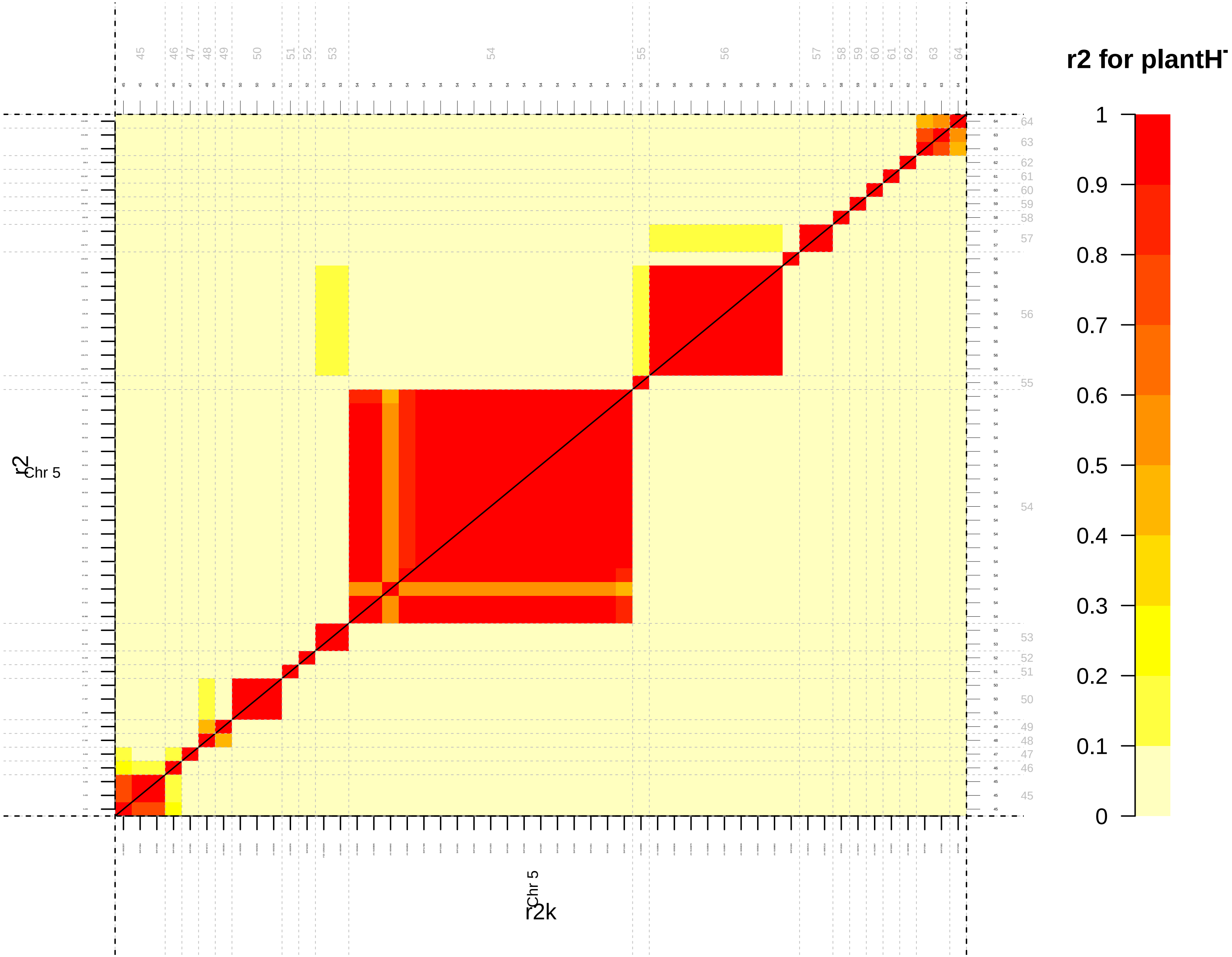

r2 for plantH
45 46 47 48 49 50 51 52 53 54 55 56 57 58 59 60 61 62 63 64
1 0.9 0.8 0.7 0.6 0.5 0.4 0.3 0.2 0.1 0
Chr 5
r2
r2k

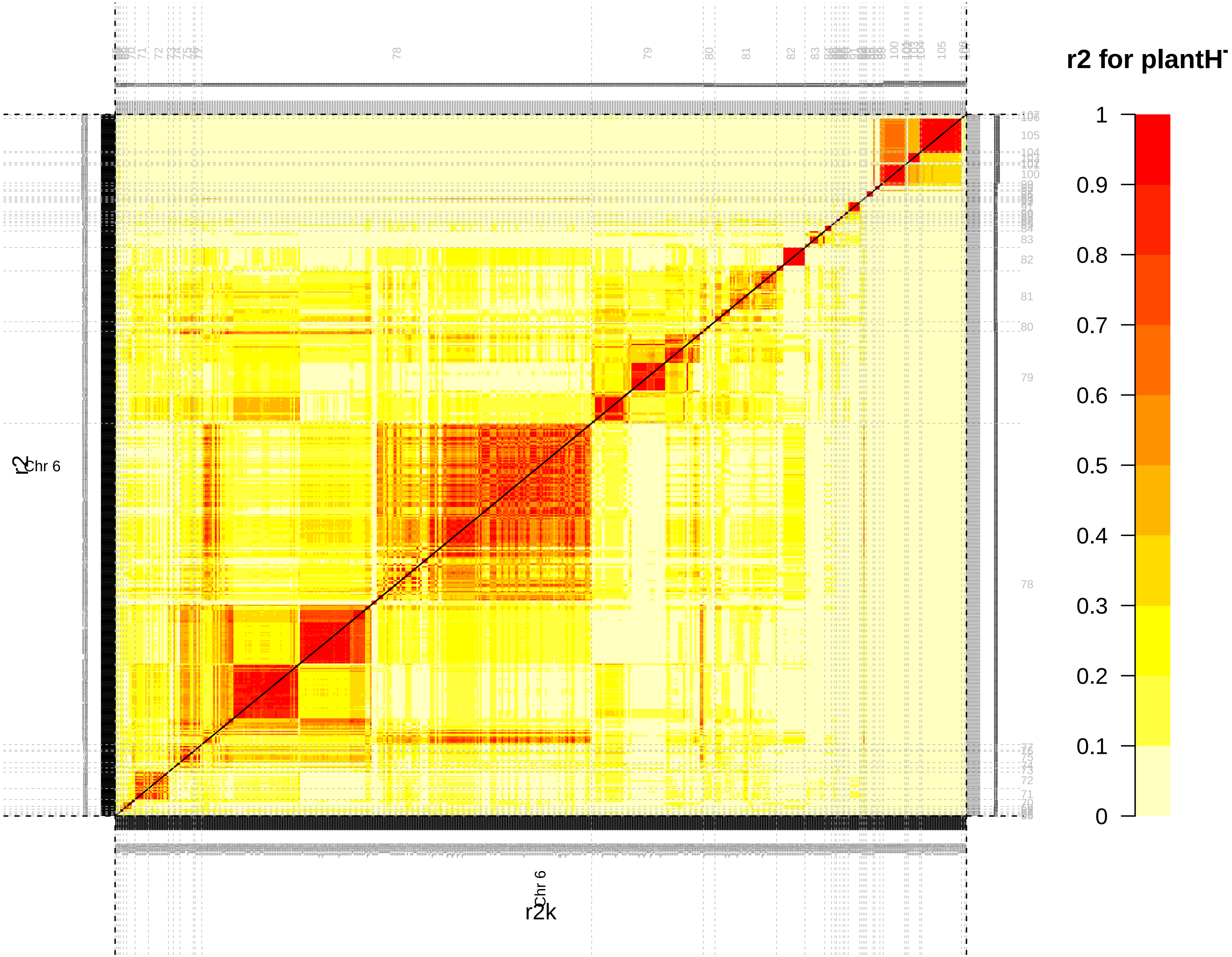
r2 for plantH
1
0.9
0.8
0.7
0.6
0.5
0.4
0.3
0.2
0.1
0
Chr 6
r2
r2k

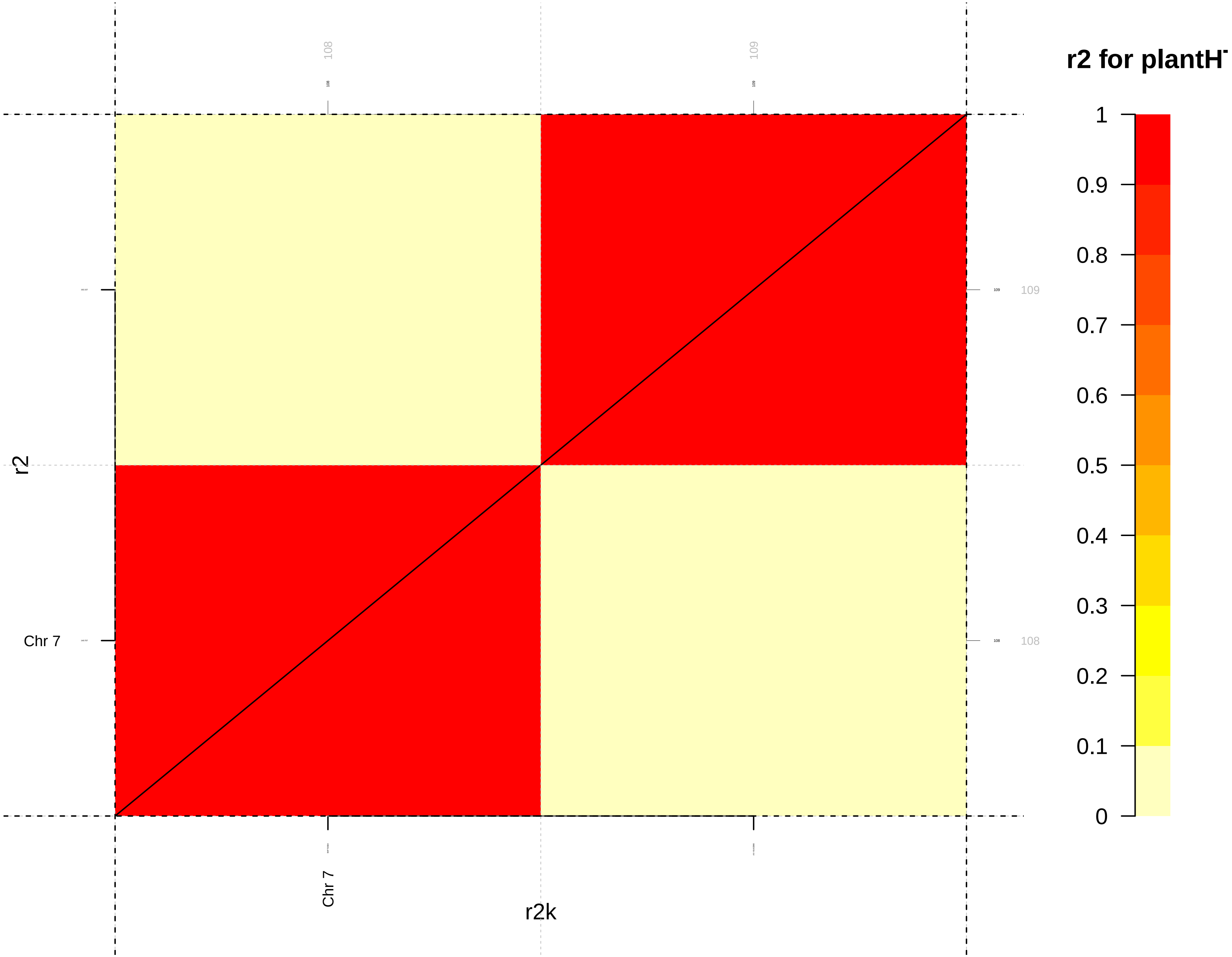

r2 for plantH
1
0.9
0.8
0.7
0.6
0.5
0.4
0.3
0.2
0.1
0
108
109
r2
Chr 7
r2k

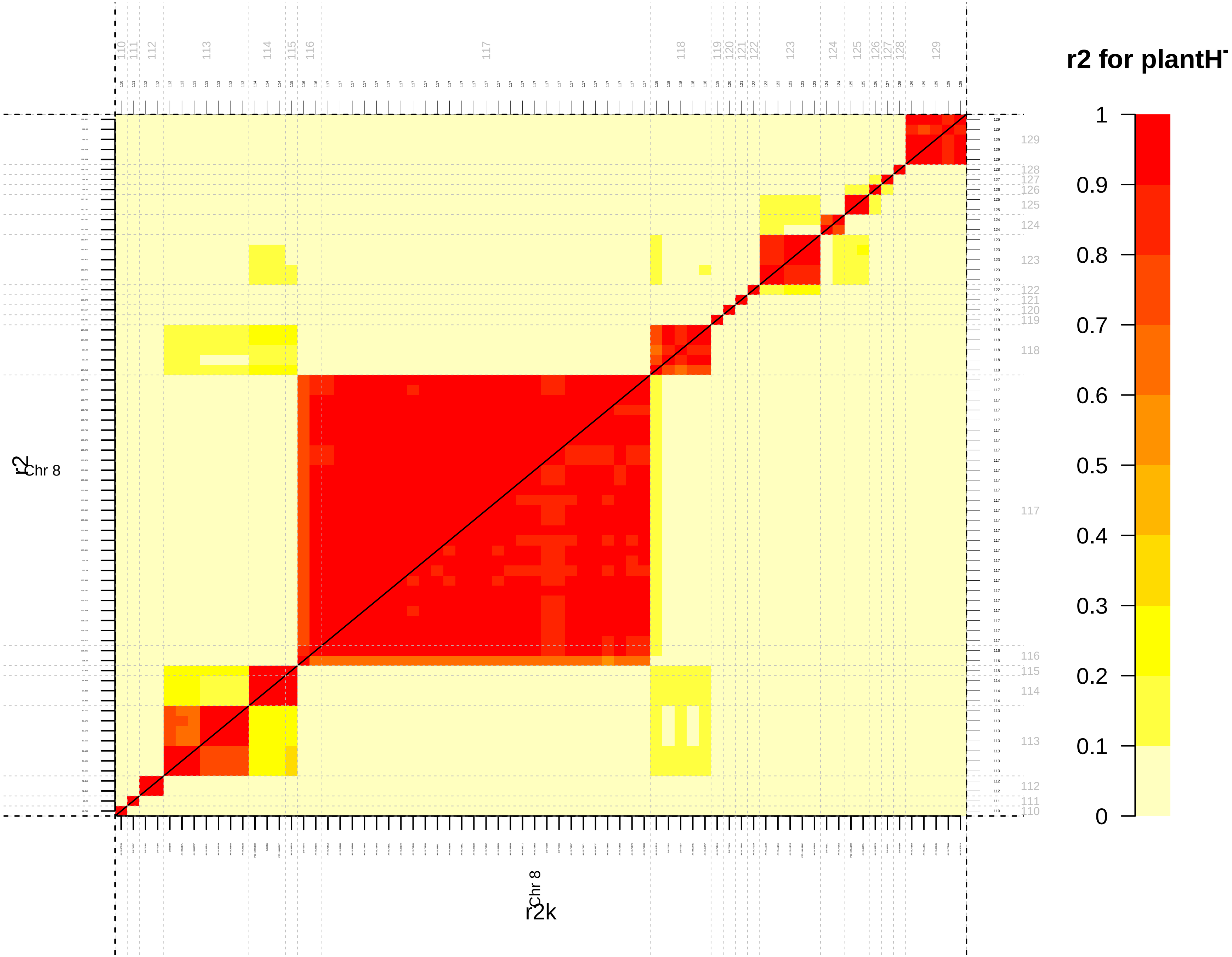
r2 for plantH
1
0.9
0.8
0.7
0.6
0.5
0.4
0.3
0.2
0.1
0
Chr 8
r2
Chr 8
r2k
110
111
112
113
114
115
116
117
118
119
120
121
122
123
124
125
126
127
128
129

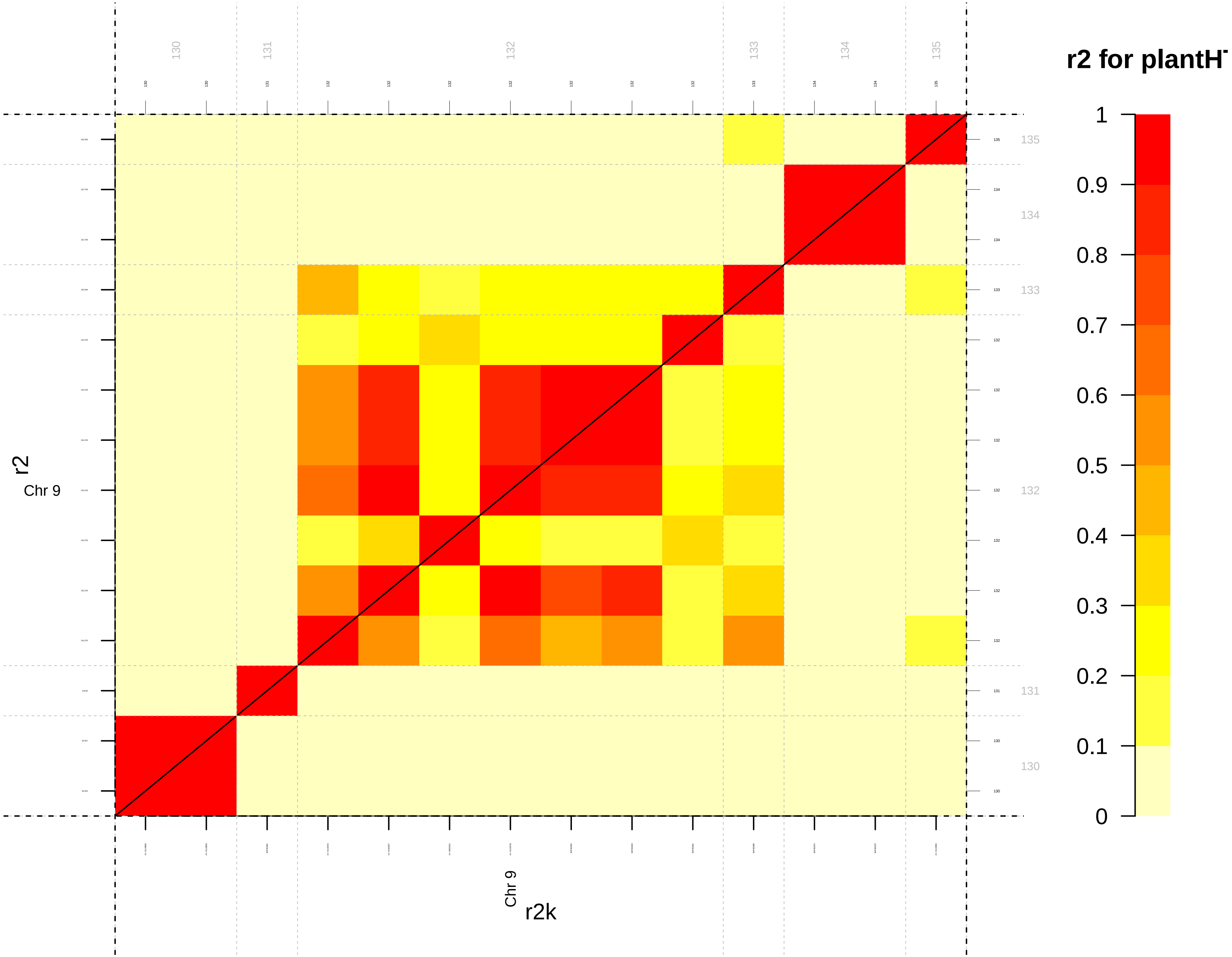
r2 for plantH
Chr 9
r2
r2k
130
131
132
133
134
135
1
0.9
0.8
0.7
0.6
0.5
0.4
0.3
0.2
0.1
0

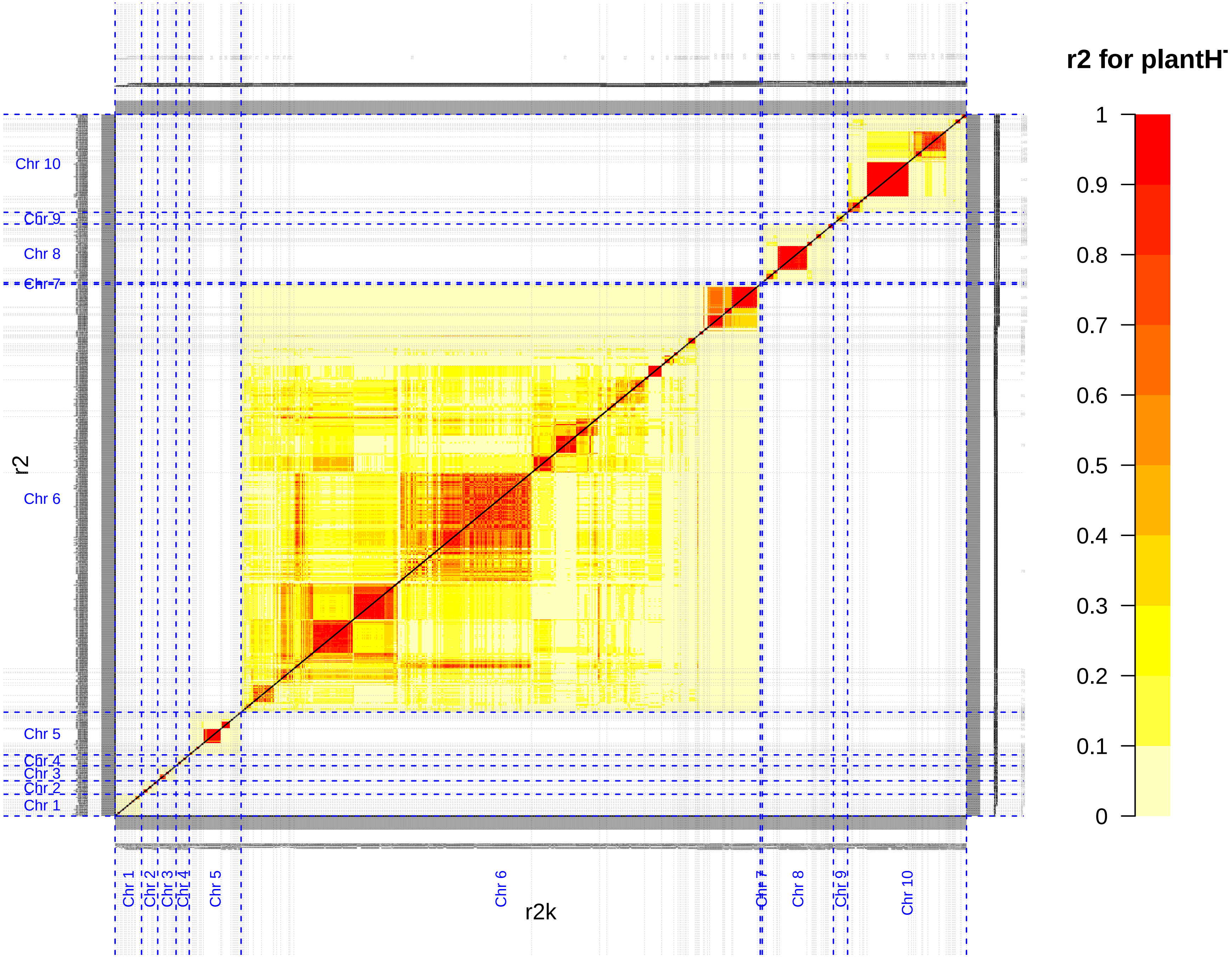
r2 for plantH
1
0.9
0.8
0.7
0.6
0.5
0.4
0.3
0.2
0.1
0
Chr 10
Chr 9
Chr 8
Chr 7
Chr 6
Chr 5
Chr 4
Chr 3
Chr 2
Chr 1
r2
r2k

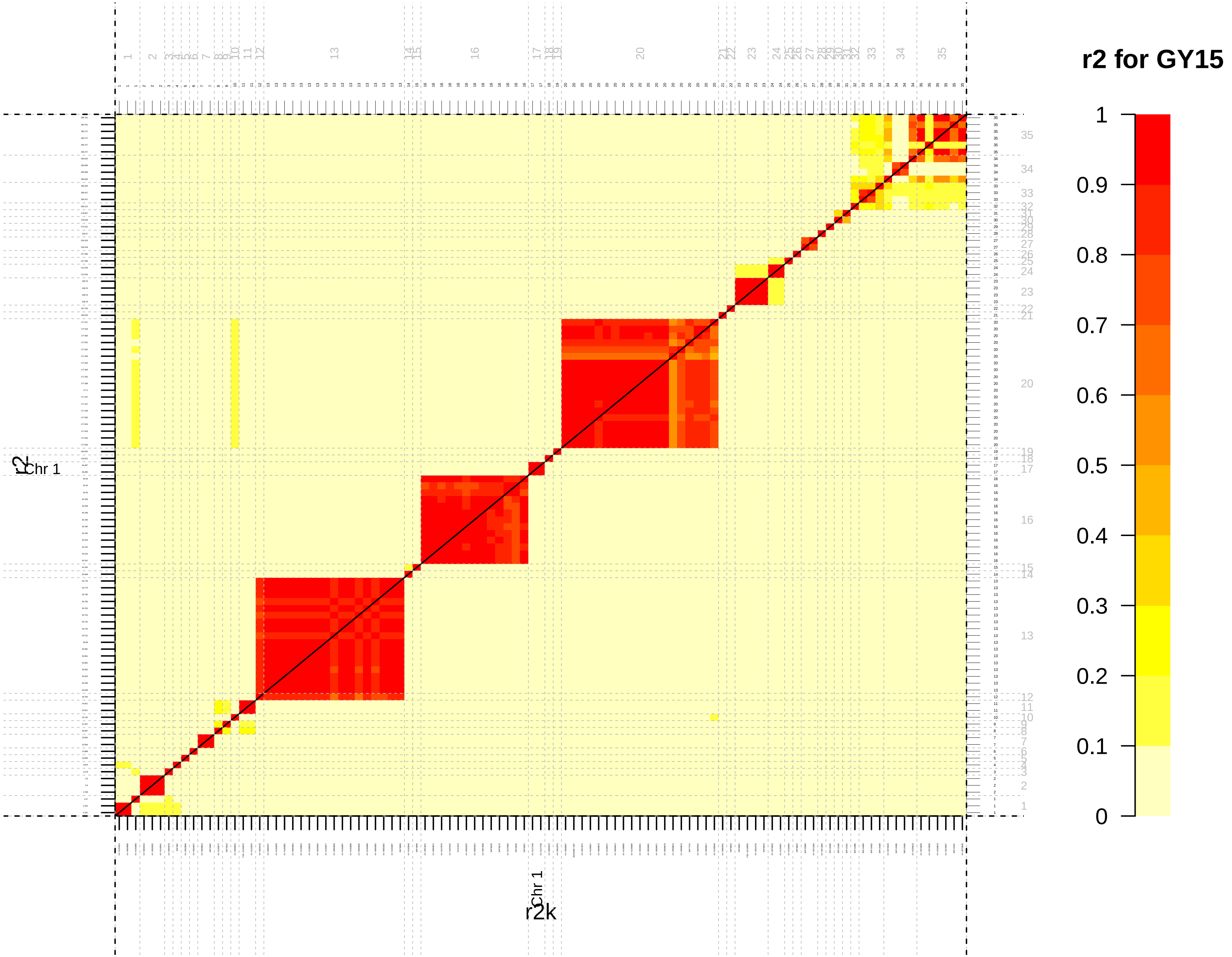

r2 for GY15
1
0.9
0.8
0.7
0.6
0.5
0.4
0.3
0.2
0.1
0
Chr 1
r2
Chr 1
r2k

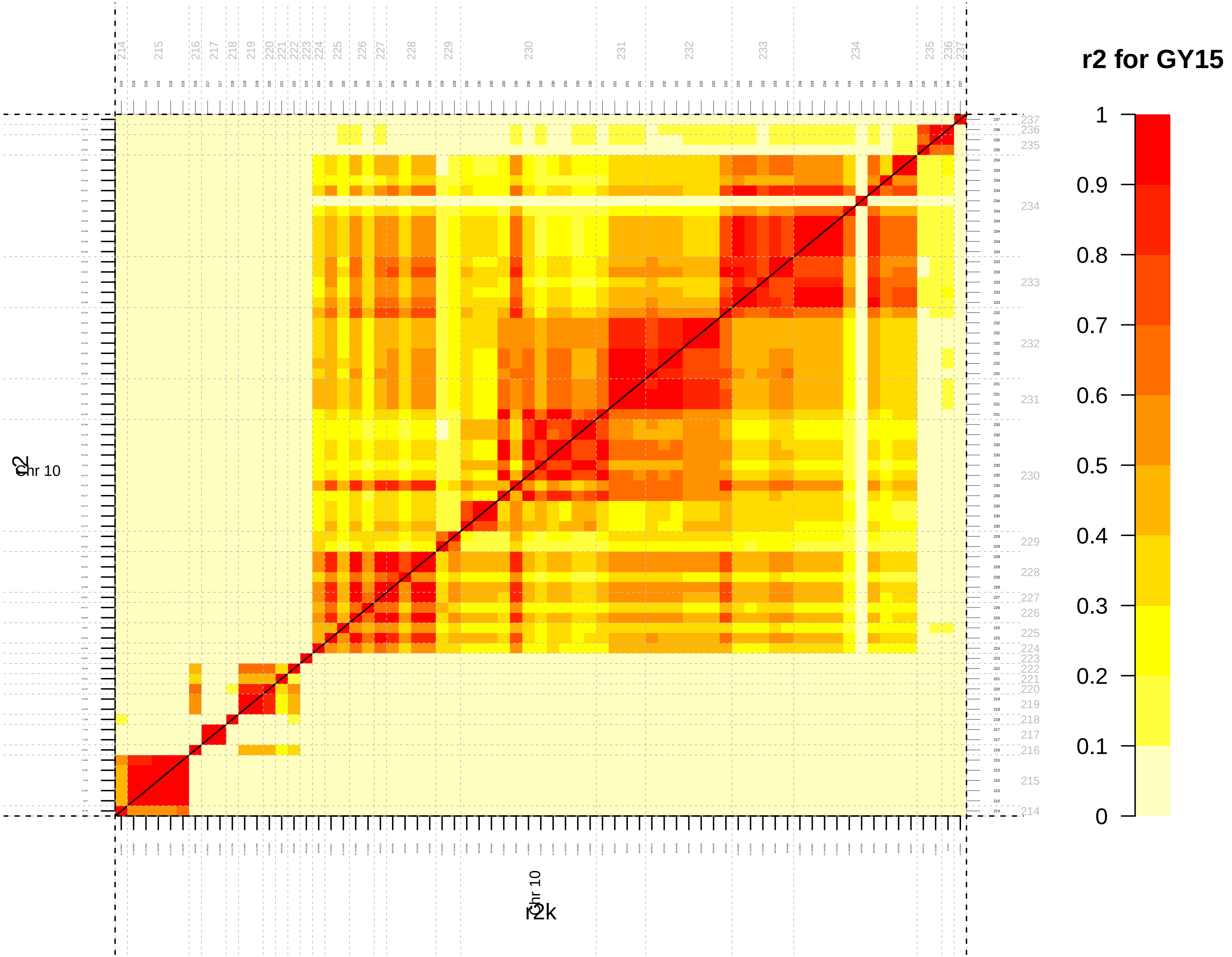

r2 for GY15
1
0.9
0.8
0.7
0.6
0.5
0.4
0.3
0.2
0.1
0
Chr 10
r2
Chr 10
r2k

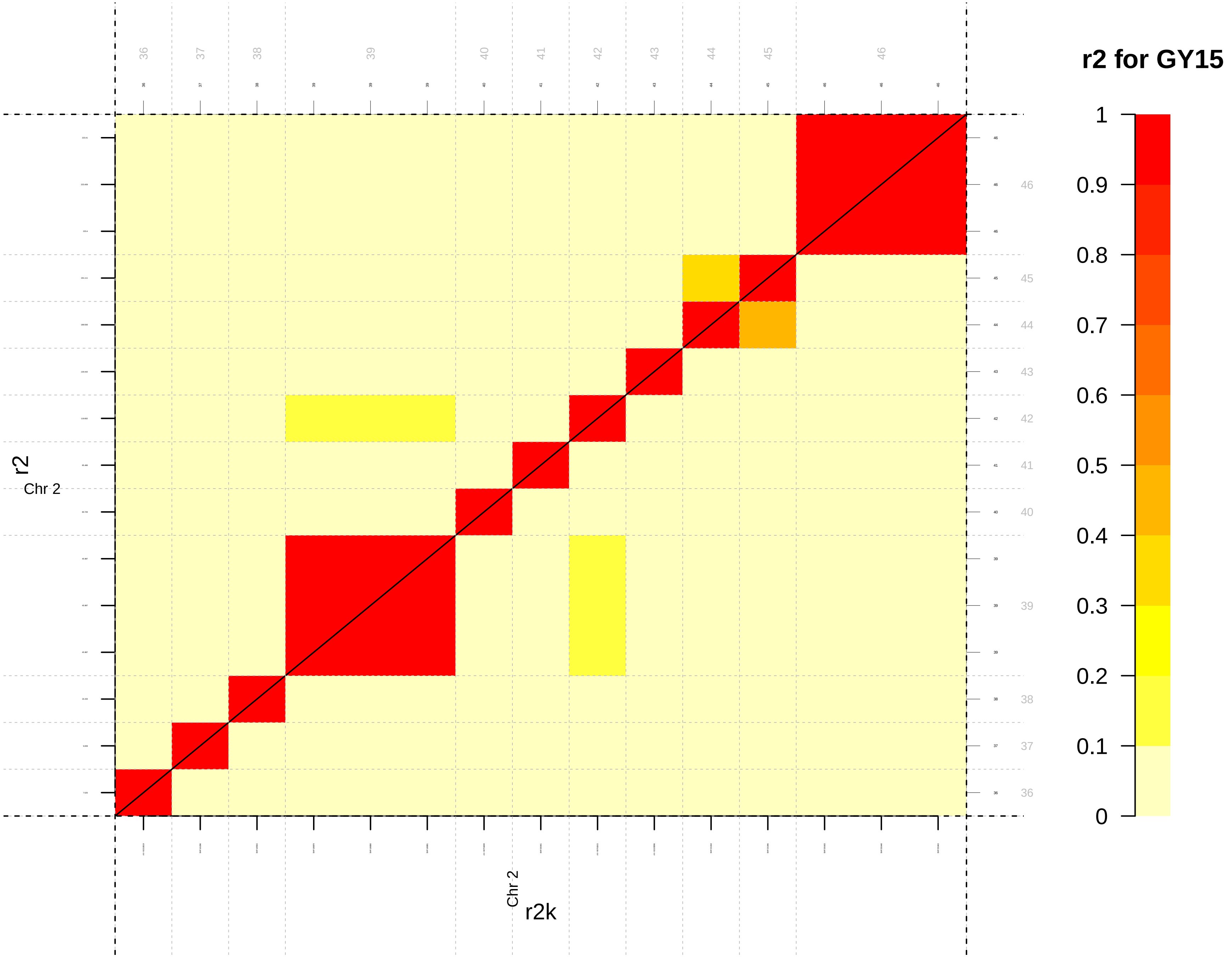
r2 for GY15
r2
Chr 2
r2k
Chr 2
36
37
38
39
40
41
42
43
44
45
46
1
0.9
0.8
0.7
0.6
0.5
0.4
0.3
0.2
0.1
0

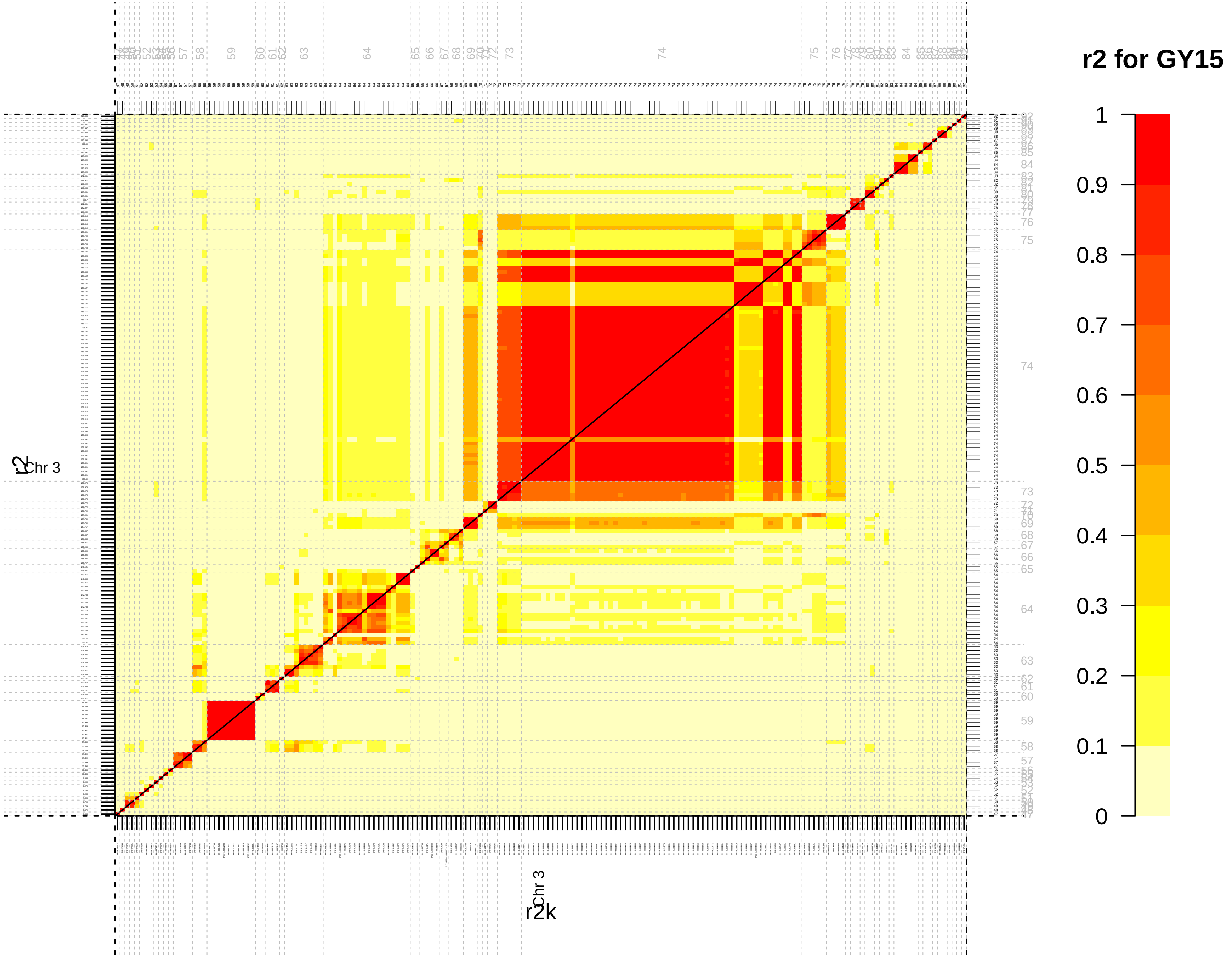
r2 for GY15
1
0.9
0.8
0.7
0.6
0.5
0.4
0.3
0.2
0.1
0
r2
Chr 3
Chr 3
r2k

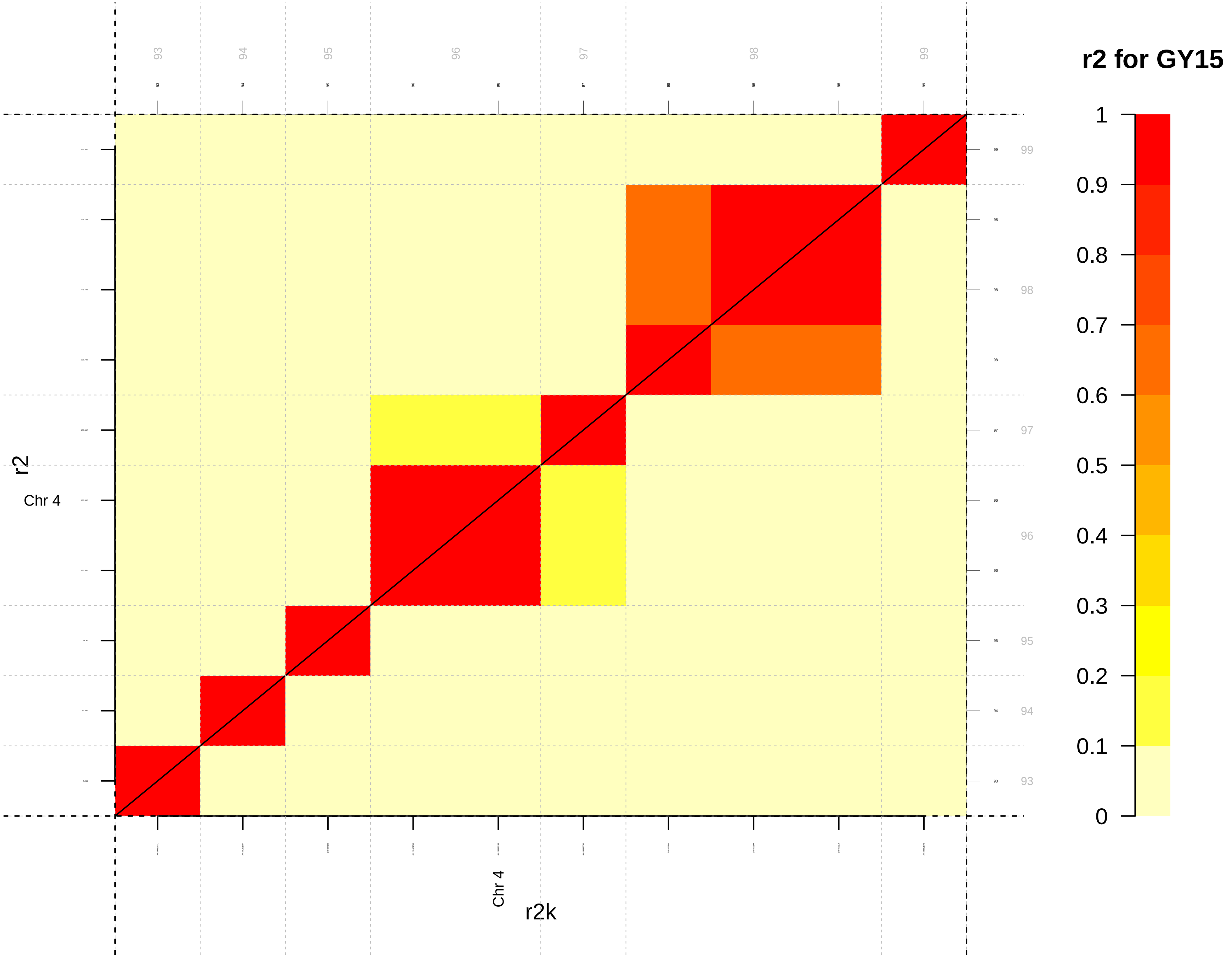
r2 for GY15
1
0.9
0.8
0.7
0.6
0.5
0.4
0.3
0.2
0.1
0
93
94
95
96
97
98
99
r2
Chr 4
r2k

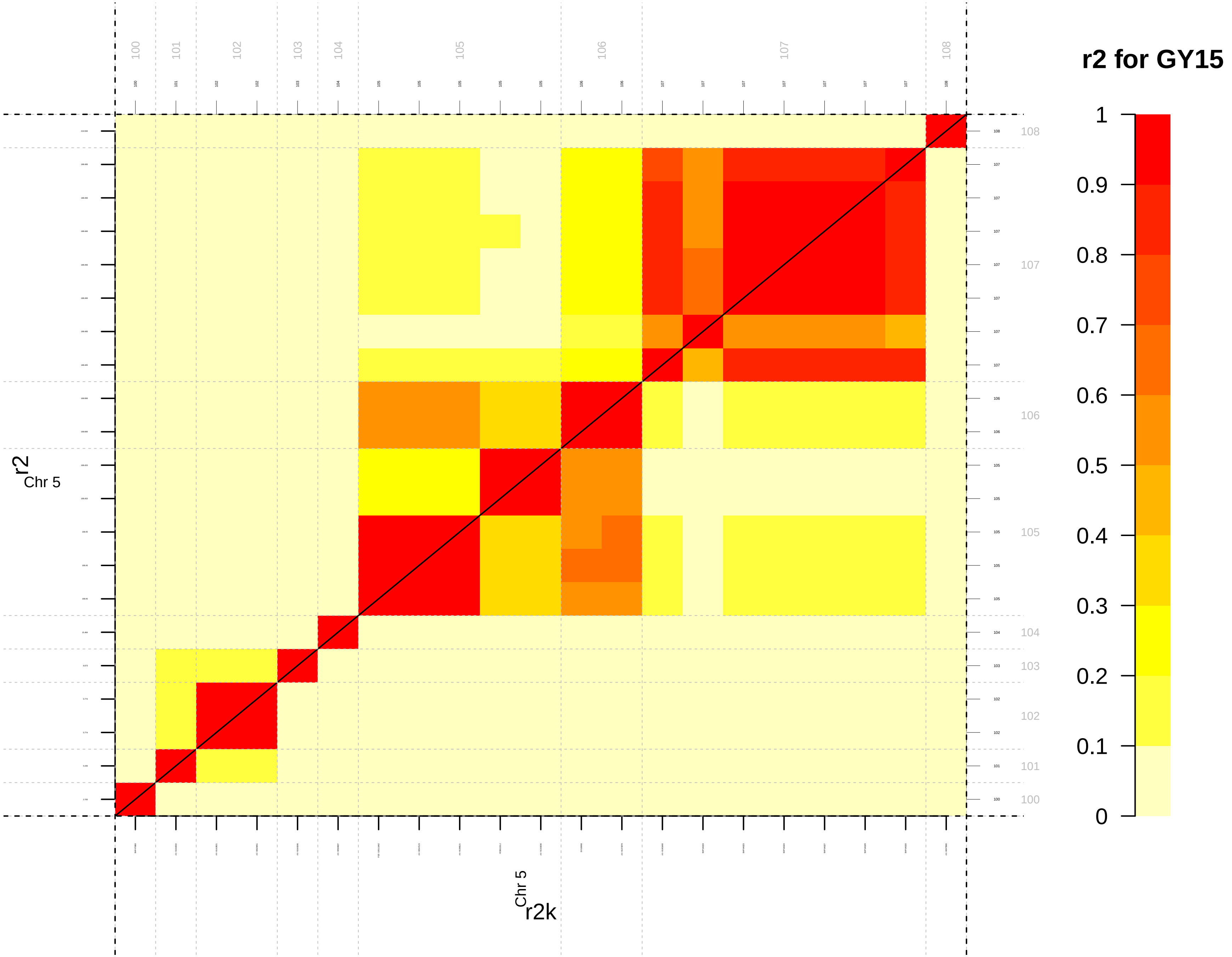

r2 for GY15
1
0.9
0.8
0.7
0.6
0.5
0.4
0.3
0.2
0.1
0
100
101
102
103
104
105
106
107
108
r2
Chr 5
r2k
Chr 5

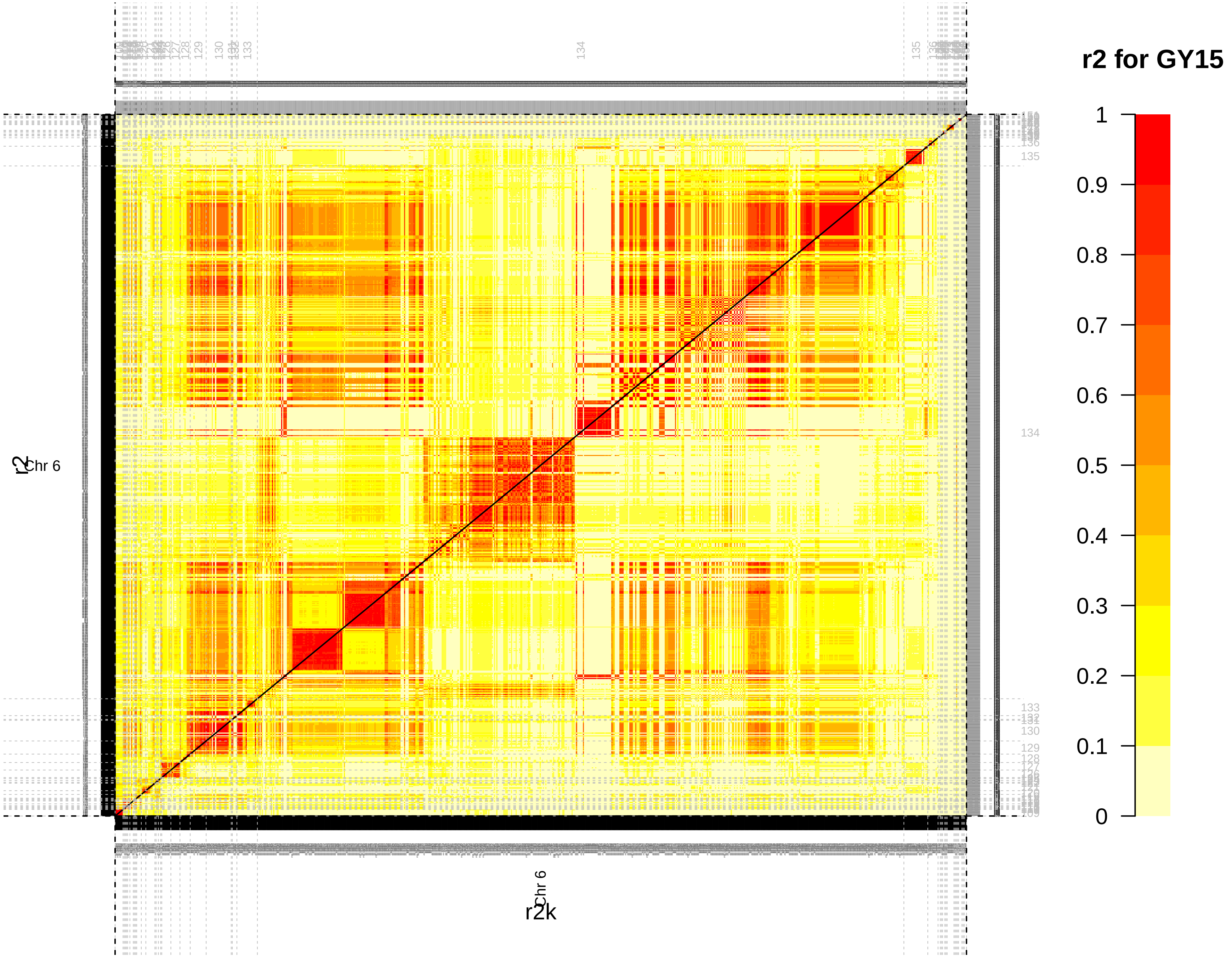
r2 for GY15
1
0.9
0.8
0.7
0.6
0.5
0.4
0.3
0.2
0.1
0
r2
Chr 6
r2k
Chr 6
134
135
133
130
129
128

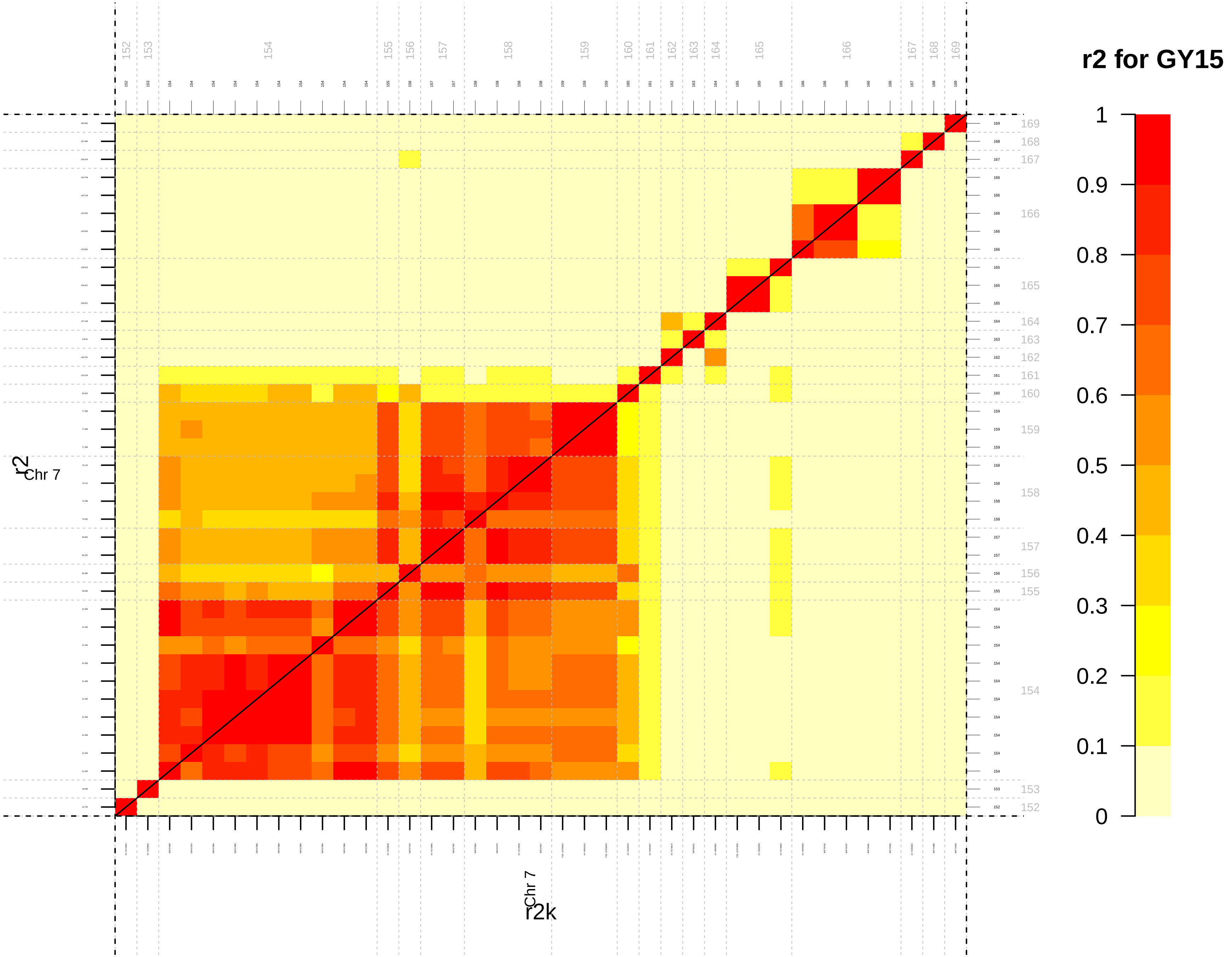

r2 for GY15
1
0.9
0.8
0.7
0.6
0.5
0.4
0.3
0.2
0.1
0
r2
Chr 7
r2k
Chr 7
152
153
154
155
156
157
158
159
160
161
162
163
164
165
166
167
168
169

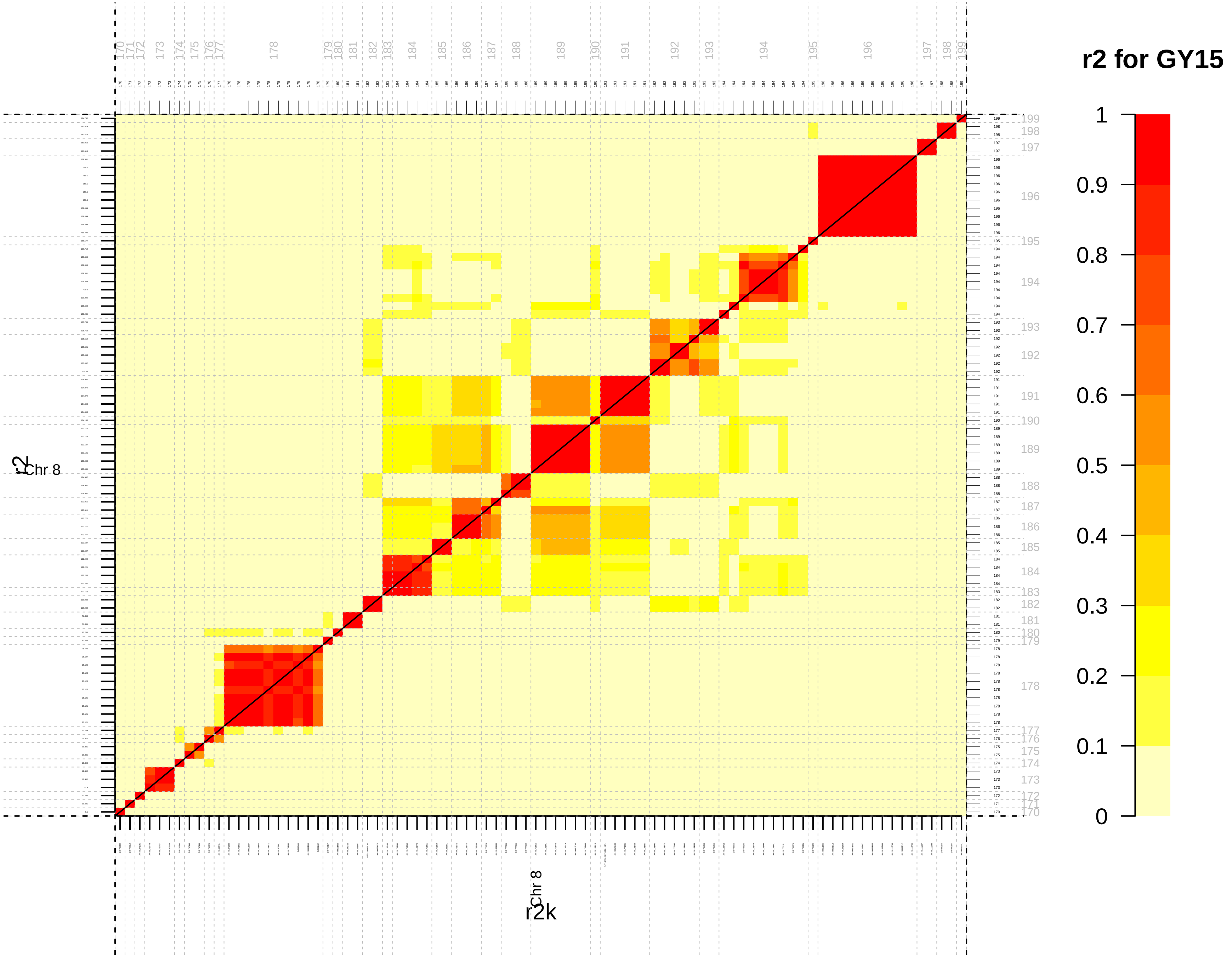
r2 for GY15
1
0.9
0.8
0.7
0.6
0.5
0.4
0.3
0.2
0.1
0
Chr 8
r2
Chr 8
r2k

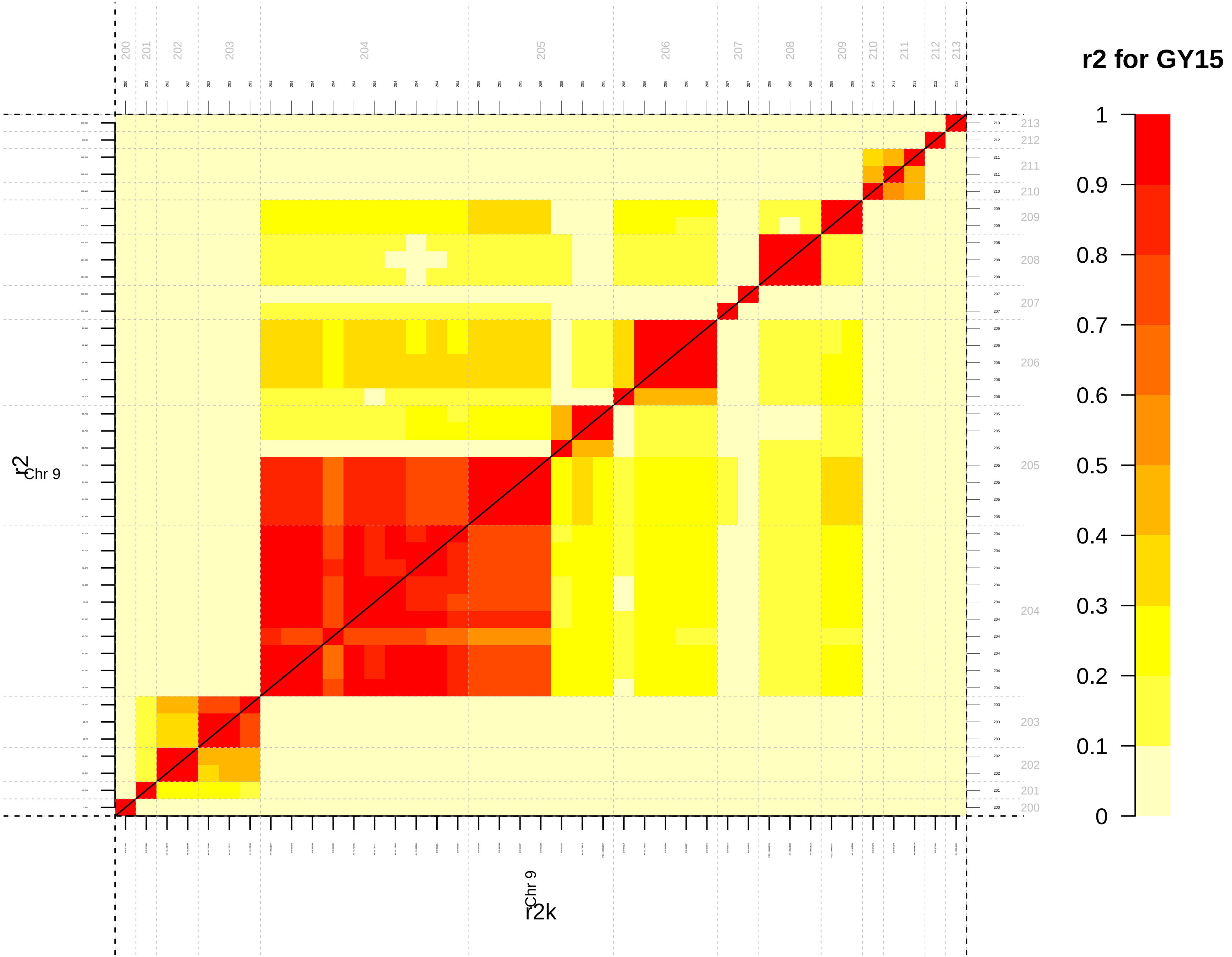
r2 for GY15
1
0.9
0.8
0.7
0.6
0.5
0.4
0.3
0.2
0.1
0
200
201
202
203
204
205
206
207
208
209
210
211
212
213
r2
Chr 9
Chr 9
r2k

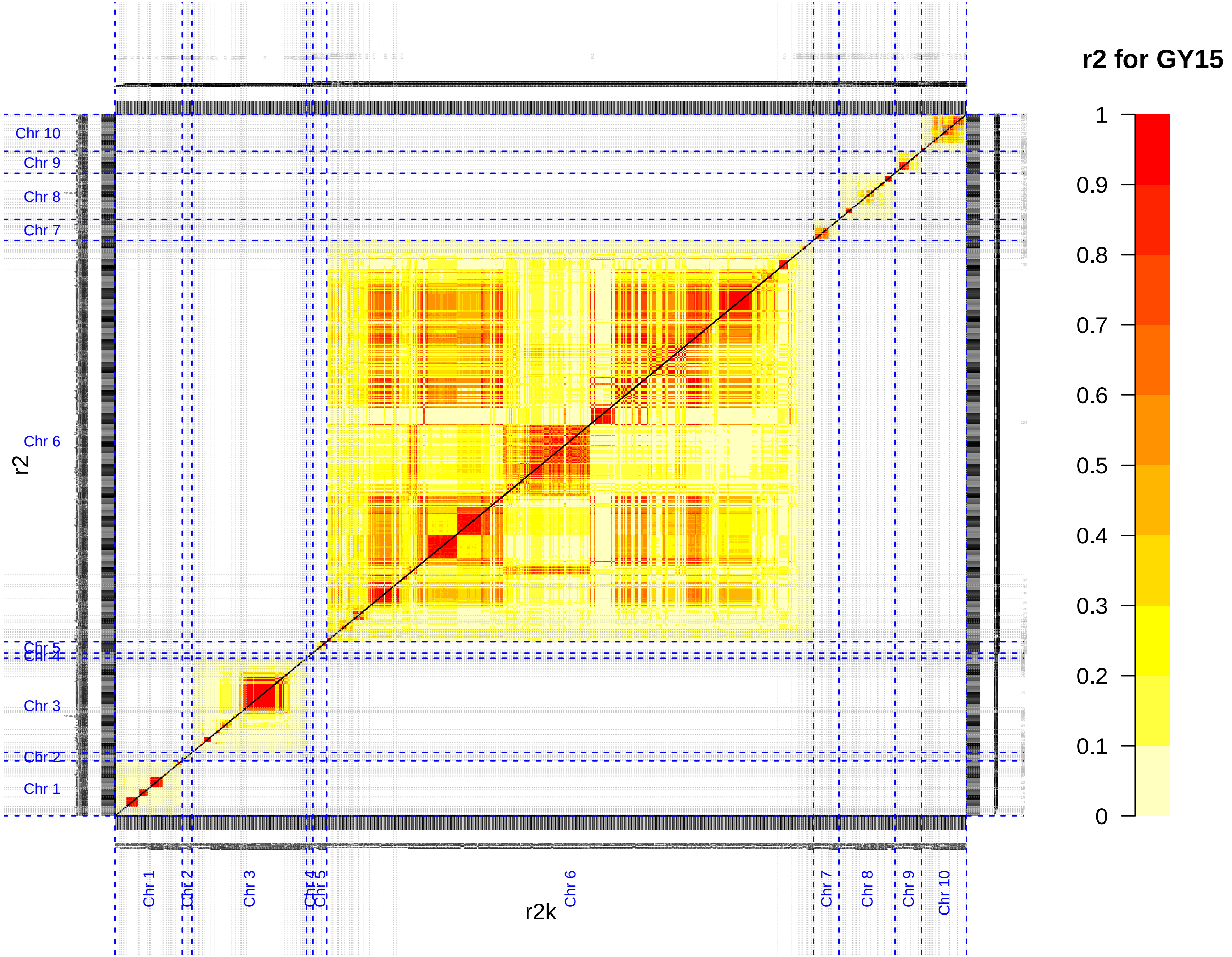
r2 for GY15
1
0.9
0.8
0.7
0.6
0.5
0.4
0.3
0.2
0.1
0
Chr 10
Chr 9
Chr 8
Chr 7
Chr 6
Chr 5
Chr 4
Chr 3
Chr 2
Chr 1
r2
r2k

Additional file 8 (.pdf):

Figure S8-LD_Windows: QTL limits obtained by the *LD_win* approach projected on heatmaps representing the level of LD between associated SNPs for each trait (DTA: male flowering time, plantHT: plant height and GY: grain yield) and each chromosome. Upper and lower triangles on the heatmaps represented the r^2 and r^2K values between associated SNPs, respectively. Linkage disequilibrium between loci was colored according to values from weak LD (yellow) to high LD (red). The significant markers were ordered according to their physical positions on the chromosome and were represented by ticks on the four sides of the heatmaps. Limits of QTLs were displayed by gray dotted lines. QTL numbers were indicated in gray on the top and the right of each heatmap.
