# Supplementary material for: Genotyping-by-sequencing and SNP-arrays are complementary for detecting quantitative trait loci by tagging different haplotypes in association studies
Source: BMC Plant Biol. 2019 Jul 16;19:318. doi: 10.1186/s12870-019-1926-4 (PMC6636005; doi:10.1186/s12870-019-1926-4)
Supplement: Supplementary file 9 — Figure S9. Number of significant SNPs (blue line) and QTLs (red line) identified as a function of SNP density (x-axis) for the male flowering time (DTA), plant height (PlantHT) and grain yield (GY). (DOCX 125 kb) [file 12870_2019_1926_MOESM9_ESM.docx]

**Figure S9: Number of significant SNPs (blue line) and QTLs (red line) identified as a function of SNP density (x-axis) for the male flowering time (DTA), plant height (PlantHT) and grain yield (GY).**
