# Supplementary material for: Genotyping-by-sequencing and SNP-arrays are complementary for detecting quantitative trait loci by tagging different haplotypes in association studies
Source: BMC Plant Biol. 2019 Jul 16;19:318. doi: 10.1186/s12870-019-1926-4 (PMC6636005; doi:10.1186/s12870-019-1926-4)
Supplement: Supplementary file 11 — Figure S10. Examples of QTL detection on Chromosome 3, 6 and 8 for the different traits. The top panel represents the distribution of the QTLs along the chromosome of interest, for the different technologies. The vertical red line in this panel localizes the SNP chosen as reference for the QTL (marker with the strongest association). The middle panel is a zoom in the vicinity of the reference SNP, showing the Local distribution of the -log10(p-value). The bottom panel is the same zoom as the middle panel and shows the local linkage disequilibrium corrected by the kinship (r2k) of all SNPs, within this region, within the reference SNP. Ticks on different x-axes show the marker density of the three technologies (red for the 50K, blue for the 600K and green for the GBS, black for all markers). (DOCX 1632 kb) [file 12870_2019_1926_MOESM11_ESM.docx]

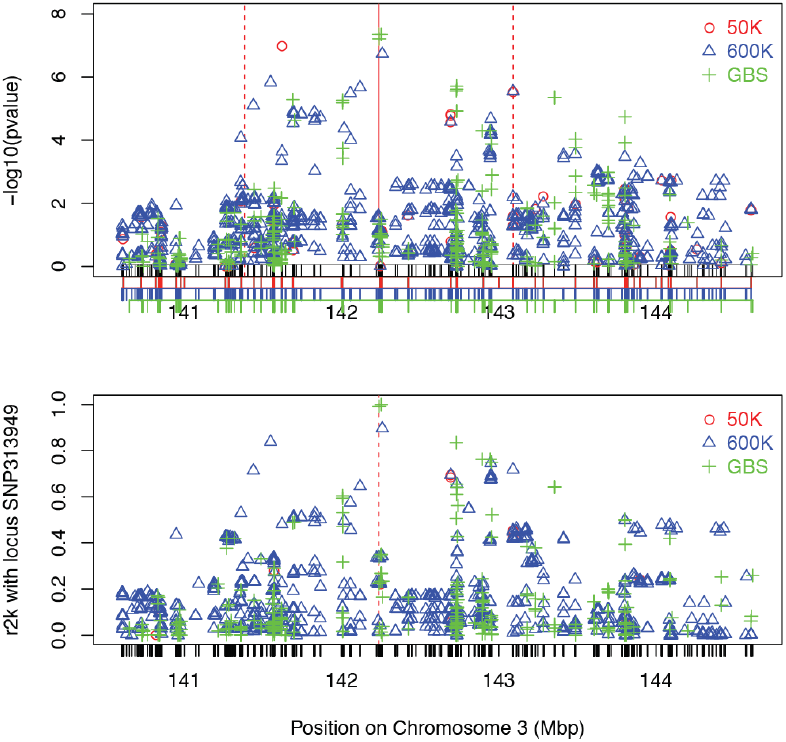

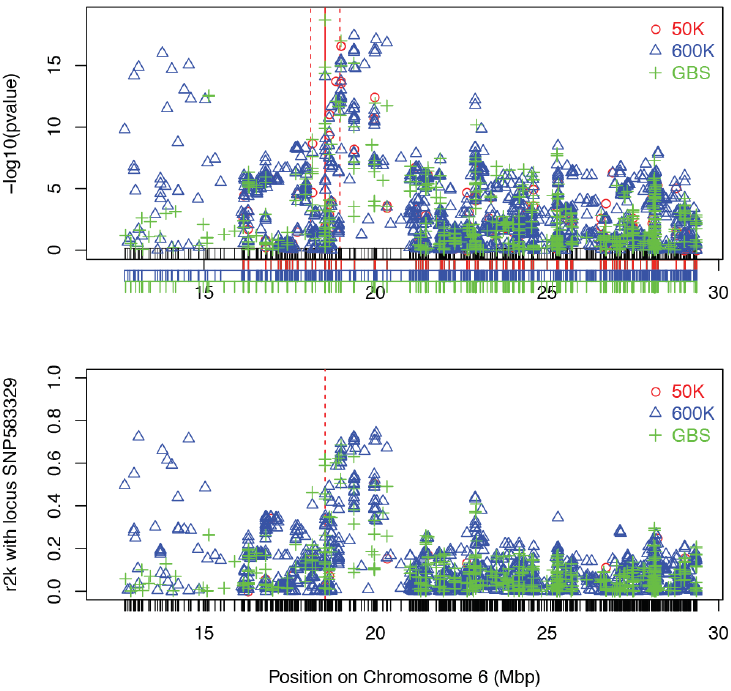

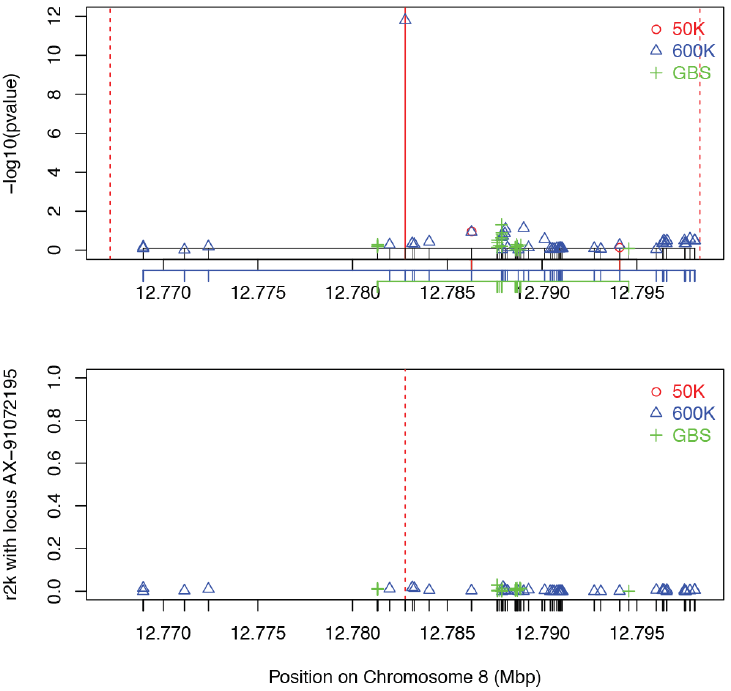


**Figure S10: Examples of comparison of QTLs detection on Chromosome 3, 6 and 8 for the different traits**.

The top panel represents the distribution of the QTLs along the chromosome of interest, for the different technologies. The vertical red line in this panel localizes the SNP chosen as reference for the QTL (marker with the strongest association). The middle panel is a zoom in the vicinity of the reference SNP, showing the Local distribution of the -*log_10_*(*p-value*). The bottom panel is the same zoom as the middle panel and shows the local linkage disequilibrium corrected by the kinship (*r^2^k*) of all SNPs, within this region, within the reference SNP. Ticks on different x-axes show the marker density of the three technologies (red for the 50K, blue for the 600K and green for the GBS, black for all markers).
