# Supplementary material for: Genotyping-by-sequencing and SNP-arrays are complementary for detecting quantitative trait loci by tagging different haplotypes in association studies
Source: BMC Plant Biol. 2019 Jul 16;19:318. doi: 10.1186/s12870-019-1926-4 (PMC6636005; doi:10.1186/s12870-019-1926-4)
Supplement: Supplementary file 13 — Figure S12. Distribution of markers, associations and QTLs according to the MAF classes for 50K, 600K GBS, and ALL technologies. A) Number of markers, B) Proportion of markers, C) Proportion of Association, D) Proportion of QTLs. (PDF 32 kb) [file 12870_2019_1926_MOESM13_ESM.pdf]

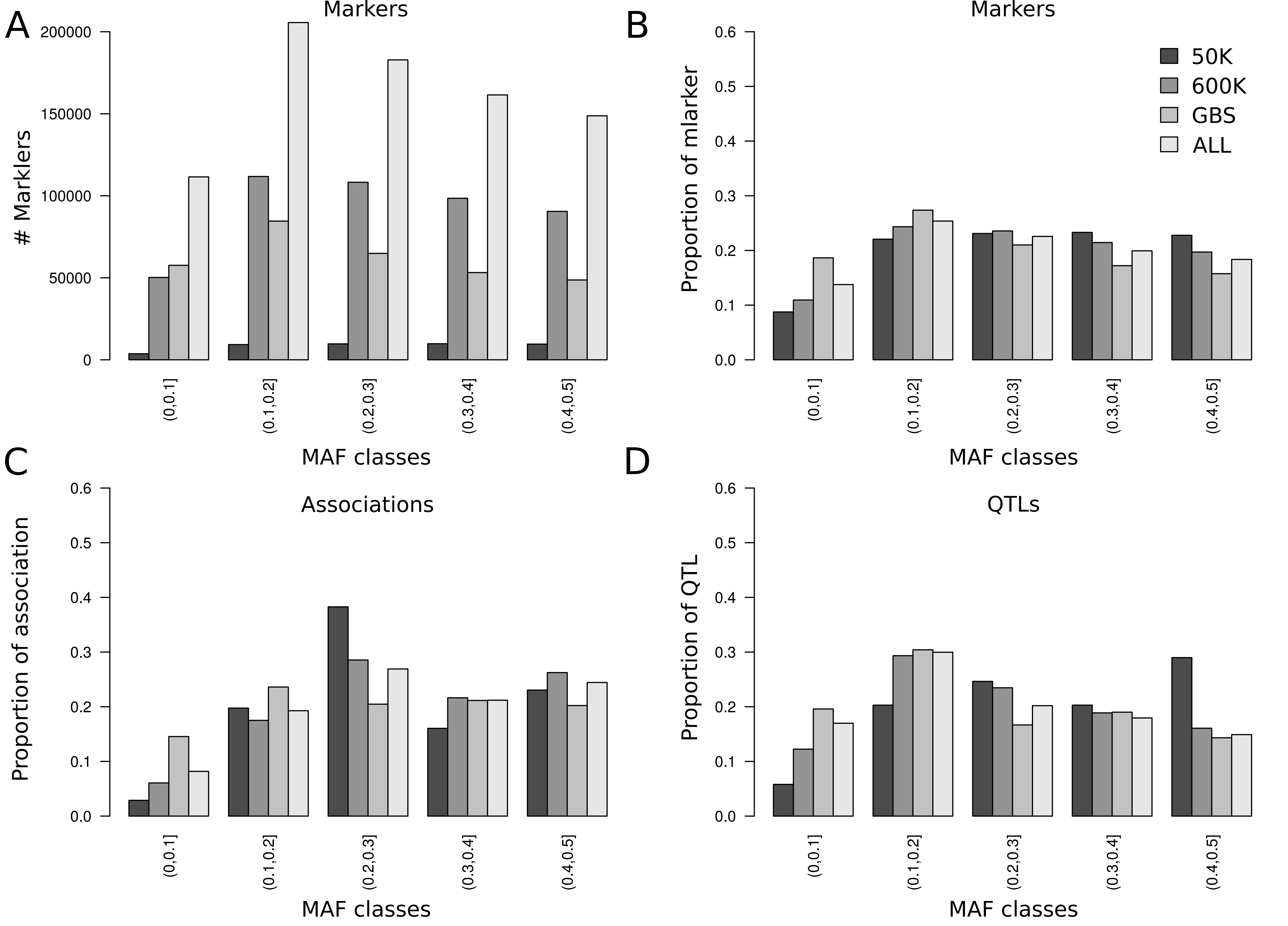
A
Markers
Marklers
200000
150000
100000
50000
0
(0,0.1]
(0.1,0.2]
(0.2,0.3]
(0.3,0.4]
(0.4,0.5]
MAF classes
B
Markers
Proportion of mlarker
50K
600K
GBS
ALL
0.6
0.5
0.4
0.3
0.2
0.1
0.0
MAF classes
C
Associations
Proportion of association
MAF classes
D
QTLs
Proportion of QTL
MAF classes

Additional file 13 (.pdf)

Figure S12: Distribution of markers, associations and QTLs according to MAF for 50K, 600K GBS, and ALL technologies. A) Number of markers, B) Proportion of markers, C) Proportion of Association, D) Proportion of QTLs
