# Supplementary material for: Genotyping-by-sequencing and SNP-arrays are complementary for detecting quantitative trait loci by tagging different haplotypes in association studies
Source: BMC Plant Biol. 2019 Jul 16;19:318. doi: 10.1186/s12870-019-1926-4 (PMC6636005; doi:10.1186/s12870-019-1926-4)
Supplement: Supplementary file 14 — Figure S13. Colocalization of QTLs between the traits. Number of QTLs specific and shared by the three traits across all environments. Note that several QTLs from one trait were sometimes included in a single QTL of another trait. (DOCX 48 kb) [file 12870_2019_1926_MOESM14_ESM.docx]

**Figure S13: Colocalization of QTLs between the traits.**

Number of QTLs specific and shared by the three traits across all environments. Note that several QTLs from one trait were sometimes included in a single QTL of another trait.
