# Supplementary material for: Genotyping-by-sequencing and SNP-arrays are complementary for detecting quantitative trait loci by tagging different haplotypes in association studies
Source: BMC Plant Biol. 2019 Jul 16;19:318. doi: 10.1186/s12870-019-1926-4 (PMC6636005; doi:10.1186/s12870-019-1926-4)
Supplement: Supplementary file 15 — Table S2. Stability of QTLs across environments for the male flowering time (DTA), Plant Height (PlantHT), Grain Yield (GY) and all traits. “Env. Nb” indicates the number of environment in which a QTL was detected. Next four columns indicate the number of QTL corresponding to each category. (DOCX 14 kb) [file 12870_2019_1926_MOESM15_ESM.docx]

**Table S2: Stability of QTLs across environments for the male flowering time (DTA), Plant Height (PlantHT), Grain Yield (GY) and all traits.**

“Env. Nb” indicates the number of environment in which a QTL was detected. Next four columns indicate the number of QTL corresponding to each category.
