# Supplementary material for: Genotyping-by-sequencing and SNP-arrays are complementary for detecting quantitative trait loci by tagging different haplotypes in association studies
Source: BMC Plant Biol. 2019 Jul 16;19:318. doi: 10.1186/s12870-019-1926-4 (PMC6636005; doi:10.1186/s12870-019-1926-4)
Supplement: Supplementary file 18 — Table S5. Narrow sense heritability (h2) and variance components (Vg, genetic variance; Ve, residual variance). The heritability and variance components were estimated for all traits (grain yield, male flowering time and plant height) using the R package Heritability [1]. (DOCX 18 kb) [file 12870_2019_1926_MOESM18_ESM.docx]

**Table S5:** Narrow sense heritability (h²) and variance components (V_g_, genetic variance; V_e_, residual variance).

The heritability and variance components were estimated for all traits (grain yield, male flowering time and plant height) using the R package Heritability [1].

Reference

1. Willem Kruijer, Martin P. Boer, Marcos Malosetti, Pádraic J. Flood, Bas Engel, Rik Kooke, Joost J. B. Keurentjes and Fred A. van Eeuwijk: **Marker-Based Estimation of Heritability in Immortal Populations.** Genetics 2015, **199** (2): 379-398.
